# Indirect costs of adult pneumococcal disease and the productivity-based rate of return to the 13-valent pneumococcal conjugate vaccine for adults in Turkey

**DOI:** 10.1080/21645515.2019.1708668

**Published:** 2020-01-29

**Authors:** J. P. Sevilla, Andrew Stawasz, Daria Burnes, Anubhav Agarwal, Basak Hacibedel, Kerem Helvacioglu, Reiko Sato, David E. Bloom

**Affiliations:** a Life Sciences Group, Data for Decisions, LLC, Waltham, MA, USA; b Health Economics and Outcomes Research, Pfizer Turkey, Istanbul, Turkey; c Health Economics and Outcomes Research, Pfizer Inc, Collegeville, PA, USA

**Keywords:** PCV13 Adult, Turkey, indirect costs, social rate of return, economic evaluation, vaccines, cost-benefit analysis

## Abstract

Productivity benefits of health technologies are ignored in typical economic evaluations from a health payer’s perspective, risking undervaluation. We conduct a productivity-based cost-benefit analysis from a societal perspective and estimate indirect costs of adult pneumococcal disease, vaccination benefits from the adult 13-valent pneumococcal conjugate vaccine (PCV13 Adult), and rates of return to PCV13 Adult for a range of hypothetical vaccination costs. Our context is Turkey’s funding PCV13 for the elderly and for non-elderly adults with select comorbidities within the Ministry of Health’s National Immunization Program. We use a Markov model with one-year cycles. Indirect costs from death or disability equal the expected present discounted value of lifetime losses in the infected individual’s paid and unpaid work and in caregivers’ paid work. Vaccination benefits comprise averted indirect costs. Rates of return equal vaccination benefits divided by vaccination costs, minus one. Input parameters are from public data sources. We model comorbidities’ effects by scalar multiplication of the parameters of the general population. Indirect costs per treatment episode of inpatient community-acquired pneumonia (CAP), bacteremia, and meningitis – but not for outpatient CAP – approach or exceed Turkish per capita gross domestic product. Vaccination benefits equal $207.02 per vaccination in 2017 US dollars. The rate of return is positive for all hypothetical costs below this. Results are sensitive to herd effects from pediatric vaccination and vaccine efficacy rates. For a wide range of hypothetical vaccination costs, the rate of return compares favorably with those of other global development interventions with well-established strong investment cases.

## 1. Introduction

Vaccination is among the most impactful public health interventions of our age.^1^ However, innovation has multiplied the number of health technologies competing for reimbursement from a health payer’s (henceforth “payer”) budget. Payers and finance ministries therefore increasingly scrutinize vaccines’ and other technologies’ value for money. Lively debate centers on the scope of value and the proper analytical framework for quantifying that value.^2–4^ We contribute to this debate by addressing productivity’s role in value assessments in the context of vaccinating Turkish adults with the 13-valent pneumococcal conjugate vaccine (PCV13) 70.

### 1.1. Choice of perspective and analysis

Two specification choices are central to value-for-money calculations. First is the relevant perspective, where the most common and important choices are the health payer’s and societal perspectives. According to the health payer’s perspective, the core elements of value are health gains–typically denominated in quality-adjusted life years (QALYs) and valued at the payer’s willingness-to-pay (WTP) per QALY–and savings to the payer’s budget (typically averted direct medical costs). According to the societal perspective, health gains should be valued at individuals’ WTP, and the broader socio-economic benefits of those health gains such as economic productivity matter as well. (QALYs can in theory reflect the productivity effects of health but in practice fail to systematically do so.^5–7^)

The second choice is whether, within the societal perspective, the broader socio-economic benefits of health should be quantified using cost-utility analysis (CUA) or cost-benefit analysis (CBA). CUA assumes a constant willingness-to-pay (WTP) per QALY regardless of, say, differential productivity consequences across QALYs (an aspect of the so-called “a QALY is a QALY is a QALY” assumption).^4^ CBA allows different units of health to have different values, reflecting among other things their differential productivity impacts. Thus, QALYs are typically not meaningful in CBA, and health improvements that are equivalent QALY gains in CUA may have different and typically age-varying values within CBA 71.

### 1.2. Productivity

An important category of value within the societal perspective is productivity because it enables consumption, which along with leisure are the two direct sources of utility within standard economics. The value of health is at least as large as that of its productivity effects since there’s more to life than consumption. Thus, productivity benefits are a lower bound measure of individuals’ WTP for health.

A first issue here is whether to adopt the human capital approach (which counts lifetime productivity losses to the individual from mortality and morbidity) or the friction approach (which counts only aggregate market production losses, which can be mitigated by replacing sick or deceased workers). We adopt the human capital approach for the following reasons. First, the societal perspective is of policy interest in part because it encompasses (among many perspectives) the perspective of the vaccinated individual. The vaccinated individual is indifferent to replacement, so the productivity that reflects the vaccinated individual’s WTP for health is given by the human capital rather than the friction cost approach. Second, the Second Panel recommends the human capital approach.^2^ It argues that even in developing countries with high unemployment, the formally unemployed are likely to be productive in informal labor markets and unpaid work, causing friction cost analyses to understate productivity losses from replacing disabled or deceased workers. Third, in our analysis we adjust paid work by employment rates to reflect the impact of high unemployment.

Traditional productivity analysis relies on market transactions to reveal value and so focuses on paid work. But unpaid work produces valuable consumable goods and services that contribute to utility so ignoring it produces downward-biased estimates of well-being, disease burdens, and vaccination benefits. Omitting unpaid work inequitably undervalues the contributions of women and the elderly, whose work is more likely to be unpaid than that of working-age men.^8^ Such omission underappreciates the harm (including to children, the sick, and the elderly) and the financial and economic costs (from having to pay a helper or impose time burdens on friends and family) that come from a loss of unpaid production. Unpaid work has grown in importance in economic research,^9^ Systems of National Accounts,^10^ economic evaluation reference cases,^2^ and well-being measurement.^11^

### 1.3. Pneumococcal vaccination policy in Turkey

Two pneumococcal vaccines exist for adults. The 23-valent pneumococcal polysaccharide vaccine (PPV23) is effective against invasive pneumococcal disease (IPD), but has questionable effectiveness against community-acquired pneumonia (CAP).^12^ PCV13 is effective against both: its efficacy against IPD as well as CAP was first demonstrated in a randomized controlled trial conducted in Dutch elderly.^13^

In Turkey, the Ministry of Health (MoH), in consultation with the Advisory Board of Immunization (ABI), decides whether to recommend vaccines for reimbursement as part of the National Immunization Program. PCV13 was first approved in Europe in 2009,^14^ and the ABI has recommended pneumococcal vaccines in children since 2008.^15^ PPV23 has been recommended and reimbursed for at-risk and elderly adults since 2007.^16^ In 2016, the ABI and MoH approved a comprehensive adult immunization program, as part of which PCV13 is now recommended and reimbursed for all adults aged 65 years and older, as well as for adults aged 18–64 years with certain comorbid diseases.^17^ Our analysis sheds light on the value for money of Turkey’s adding PCV13 Adult reimbursement to the pre-2016 status quo of adult PPV23 reimbursement.

### 1.4. Literature on economic evaluation of PCV13 adult

The literature on the economic evaluation of PCV13 Adult typically adopts the health payer’s perspective,^18^ uses CUA, ignores indirect costs, and where indirect costs are considered uses the friction approach and ignores unpaid work. Dirmesropian et al. (2015)^19^ review pre-2015 studies a majority of which found PCV13 adult to be cost-effective but all of which were hampered by not having access to the important CAPITA trial estimates of vaccine efficacy. A more recent review by Treskova et al. (2019)^18^ stratifies the newer literature according to assumptions about herd effects from pediatric vaccination (i.e., whether effects were ongoing or completed, whether effects were from pediatric PCV13 (PCV13 Pediatric) or from pediatric 10-valent pneumococcal conjugate vaccine (PCV10 Pediatric), or whether there was a switch in pediatric vaccination from PCV10 to PCV13 or vice versa), and according to the policy being evaluated (PPV23 versus no vaccination, PCV13 versus no vaccination, and adding PCV13 to PPV23). Of the four studies that evaluate the addition of PCV13 Adult to adult PPV23 (as was the case in Turkey), van Hoek and Miller (2016),^20^ Blommaert et al. (2016),^21^ and Willem et al. (2018)^22^ found doing so to be cost-ineffective, while Stoecker et al. (2016)^23^ found it to be cost-effective only in the short run before herd effects fully set in. Treskova et al. (2019)^18^ conclude from this evidence that PCV13 Adult is not cost-effective in the presence of herd effects from PCV13 Pediatric.

Economic evaluation of adult pneumococcal vaccination in Turkey exists, but these either focus on adult PPV23^16^ or focus on PCV but do so from a health payer’s perspective and without the benefit of CAPITA vaccine efficacy estimates.^24,25^ Two evaluations of PCV13 Adult consider productivity losses: Mangen et al.(2015)^26^ and Heo et al. (2017).^27^ Both ignore unpaid work, the former uses friction costs to estimate the productivity losses from mortality, and the latter does not consider productivity losses from mortality.

## 2. Methods and data

This section describes our methods and data. The Methodological Appendix provides details.

### 2.1. Objectives

We conduct a productivity-based CBA from a societal perspective. Our societal perspective reflects multiple interests, including those of the individual, household, employer, public sector, and larger society. Our societal perspective is a limited rather than full version because we include only productivity benefits and ignore the intrinsic value of health (this is the value captured by QALYs in CUA), direct costs, and non-health-sector benefits other than productivity. Though direct costs are typically included in economic evaluations, we omit them to focus on the importance of indirect costs to vaccine evaluation and to assess whether PCV13 represents strong value-for-money on indirect cost grounds alone.

Our CBA allows for age-varying productivity benefits across Turkish adults of all ages. We take productivity gains to be a lower-bound measure of patient- and societal WTP for health gains. For reasons given above, we adopt a human capital approach to productivity loss and incorporate three central categories of unpaid work: housework, caregiving, and volunteering.^28^ Indirect costs from disease equal the loss in expected present discounted value of lifetime pre-tax paid work and unpaid work from disease. Vaccination benefits consist of averted indirect costs from vaccination.

We calculate (i) the per treatment episode indirect costs of adult pneumococcal disease (PD), (ii) the per person vaccination benefits from PCV13 Adult, and (iii) hypothetical rates of return to PCV13 Adult for a range of hypothetical vaccination costs. We perform these calculations in the context of Turkey’s funding PCV13 for both all elderly adults in the general population (aged 65 and up, hereafter the “elderly subpopulation”) and non-elderly adults with select comorbidities (aged 18–64, hereafter the “comorbid subpopulation;” we refer to the elderly and comorbid subpopulations combined as the “program population”) within the Ministry of Health’s National Immunization Program. The comorbid subpopulation is limited to those with the following six comorbidities: chronic obstructive pulmonary disease, asthma, diabetes, chronic kidney failure, chronic heart disease, and cancer.^17^

### 2.2. Approach to calculating societal WTP for PCV13

Different elements of how we define productivity are relevant to different interests, as the following sections describe.

#### 2.2.1. Vaccinated individual

For the vaccinated individual, assuming that no income transfers occur between individuals, lifetime post-tax earnings, along with nonmarket production, constrain how much market and nonmarket goods and services the individual can consume. Such consumption is itself a lower-bound measure of lifetime wellbeing because other factors, such as leisure, also contribute to wellbeing.^29,30^

#### 2.2.2. Transfer beneficiaries

Whatever an individual earns or produces but does not consume can be shared with others, such as household members and friends (the individual’s “transfer beneficiaries”). The individual’s lifetime post-tax earnings and nonmarket productivity therefore capture not only the vaccinated individual’s wellbeing, but also that of his or her transfer beneficiaries.

#### 2.2.3. Employers

Disabled individuals or, in the case of employees with sick leave, employers, bear productivity losses during periods of temporary disability.

#### 2.2.4. Fiscal authorities

We value paid work at pre-tax earnings to partially capture fiscal losses from disease.

### 2.3. Model structure

**Figure 1.**
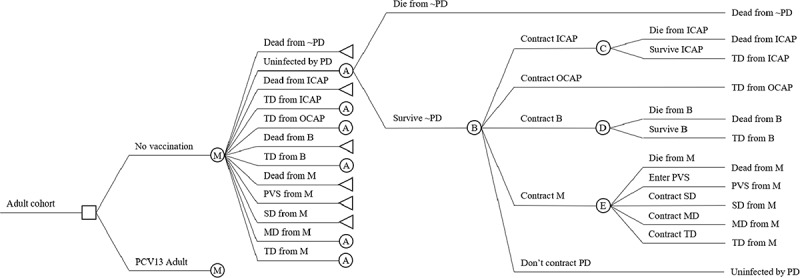
Markov model diagram. Abbreviations: B = bacteremia; ICAP = inpatient community-acquired pneumonia; M = meningitis; MD = moderate disability; OCAP = outpatient community-acquired pneumonia; PCV13 Adult = 13-valent pneumococcal conjugate vaccine for adults; PD = pneumococcal disease; PVS = persistent vegetative state; SD = severe disability; TD = temporary disability

We use an age-differentiated single-cohort Markov model of disease with one-year cycles (Figure 1).^31^

An uninfected cohort faces a one-time decision to vaccinate, represented by the square node. Depending on the decision, the cohort enters the annual Markov cycle tree at node “M.” It subsequently faces sequential risks of non-PD-related death, infection with one of four PD manifestations (inpatient CAP, outpatient CAP, bacteremia, and meningitis), and these infections’ associated outcomes (death and various disabilities). Each PD-related Markov state (i.e., states other than the uninfected and non-PD death states) is a manifestation-specific outcome and has a state utility equal to the indirect costs associated with that outcome. Absorbing states terminate in left-pointed triangles. We label nodes with capitalized letters A–E for ease of reference (for example, all nodes labeled “A” have the same structure and can be referred to using the same label). Indirect costs of each state equal the loss in expected present-discounted value of lifetime pre-tax paid work and unpaid work associated with that state. The uninfected state and death from non-PD-related causes do not involve PD-related costs and thus have zero state utility. All other states involve PD-related costs and so have positive state utility.

Vaccination produces benefits by reducing risks of contracting PD (see node B in Figure 1) and therefore risks of transitioning to states with positive state utilities. The benefit of vaccination is the difference in expected discounted lifetime costs (or expected discounted cumulative state utilities) between vaccinated and unvaccinated cohorts. The policy comparison of our evaluation is therefore one actually faced by the Turkish Ministry of Health: “PCV13 program versus no PCV13 program,” where we assume the impact of the existing reimbursement of PPV23 to be constant between the two alternatives and reflected in the incidence rates of the “no PCV13 program” arm.

### 2.4. Demographic structure and temporal horizon

We apply the Markov model to a single cohort consisting of the program population in the program’s first year, thus measuring the benefit to vaccination in that year. This program population consists of two subpopulations: the elderly and comorbid subpopulations. The members of our single cohort are differentiated by (and therefore have transition probabilities and state utilities differentiated by) age and comorbidity status (i.e., a binary indicator for whether or not an individual has at least one comorbidity). We do not disaggregate cohort members or results by gender. Evidence from Sweden suggests that the elderly perform well on instrumental activities of daily living (ADLs) until age 84 and show little impairment with respect to personal ADLs until age 90.^32^ This suggests that age 85 may be a threshold beyond which functional disability will impair the ability to perform unpaid work. This consideration, along with data scarcity for ages after 85, lead us to conservatively assume that no individual survives longer than 85 years of age. The longest any cohort-member stays in the model is 68 years (i.e., an 18-year-old who lives to age 85). Our analysis covers the calendar years 2015–2082.

### 2.5. Background mortality

We derive probabilities of non-PD-related death during a cycle (see node A in Figure 1) from Eurostat life tables for Turkey, and adjust them to control for PD-related mortality (see the Methodological Appendix for details).^33^

### 2.6. Incidence

We assume adult vaccination does not reduce the severity of infection.^34^ Lack of reliable estimates also leads us to assume no serotype replacement or herd effects from adult vaccination. These assumptions limit the impact of adult vaccination to reducing PCV13-serotype (vaccine-type PD) incidence (as opposed to also reducing the probability of severe sequelae like death), and imply that we can limit our attention to vaccine-type (as opposed to all-type) incidence and vaccine efficacy.

Our model allows at most one treatment episode (i.e., inpatient or outpatient medical visit) per individual per cycle. In an unvaccinated cohort, we set the probabilities of developing the various manifestations of PD equal to the relevant incidence rates (i.e., the number of infections per 100,000 person years). Thus, we implicitly assume only one treatment episode per infection, which is conservative since infections may involve multiple treatments.

We use Turkey-specific 2017 Global Burden of Disease (GBD) estimates of lower respiratory infection (LRI) incidence,^35^ global population attributable fractions of LRI due to pneumococcal pneumonia,^36^ a 19.4% hospitalization rate,^16^ and 68% PCV13 vaccine-type coverage to derive vaccine-type inpatient and outpatient CAP incidence rates.^37^ We use Turkey-specific 2017 GBD^35^ estimates of pneumococcal meningitis incidence and 2016 European Centre for Disease Prevention and Control (ECDC)^38^ data on IPD cases and deaths for six comparator countries (Czech Republic, Greece, Hungary, Poland, Slovakia, and Slovenia) disaggregated by clinical presentation, along with a 68% PCV13 vaccine-type coverage rate, to derive vaccine-type bacteremia and meningitis incidence. These comparator countries were chosen based on the following criteria: (i) they have 2016 ECDC data on both the number of IPD cases and number of IPD deaths; (ii) they are in geographically close proximity to Turkey (i.e., in or adjacent to Central Europe or Eastern Europe); (iii) the per capita GDP (denominated in 2016 international dollars) is close to, or slightly higher than, Turkey’s; and (iv) the Socio-Demographic Index (SDI)^39^ is equal to, or slightly higher than, Turkey’s. See the Methodological Appendix for more details on incidence rate construction.

### 2.7. Herd effects from pediatric vaccination

We assume that herd effects from Turkey’s national pediatric PCV13 vaccination program will reduce steady state PCV13-type incidence rates among adults by the same 88% reduction observed in seven-valent pneumococcal conjugate vaccine (PCV7) type incidence rates in Denmark and in the United Kingdom after the introduction of PCV7.^40,41^ We also assume that part of the transition to the steady state has already occurred by 2015 (our model’s base year) and that the remainder of the transition will occur from 2016 to 2019 (see the Methodological Appendix for details).

### 2.8. Vaccine efficacy

We derive vaccine efficacy from the Community-Acquired Pneumonia Immunization Trial in Adults (CAPITA) study.^13^ We let baseline vaccine efficacy (i.e., initial vaccine efficacy, before waning) decline with age among those aged 50 and up in order to account for immunosenescence following the van Werkhoven et al. (2014)^42^ Cox-proportional hazard-based analysis of CAPITA study results. This analysis implies baseline vaccine-type efficacies against inpatient and outpatient CAP of 0.80 at age 50 and 0.27 at age 85. The corresponding figures for bacteremia and meningitis are 0.95 and 0.58, respectively. We assume constant proportional decline at intermediate ages. Since van Werkhoven et al. (2014)^42^ do not provide vaccine efficacies for ages younger than 50, we follow Mangen et al. (2015)^26^ in assigning the baseline vaccine efficacy for 50-year-olds to individuals aged 18–49. This is conservative in light of the van Werkhoven et al. (2014)^42^ finding that PCV13 vaccine efficacy declines with age, and the general phenomenon of immune response declining with age.

We allow vaccine efficacy to wane over time. Consistent with previous economic modeling of PCV13 Adult,^21^ we assume that vaccine efficacy is constant for the five years immediately following vaccination, declines 5% annually for the next five years, then declines 10% annually for the next five years, and falls to zero thereafter.

We call the vaccine efficacies resulting from the above assumptions our base-case vaccine efficacies.

### 2.9. Case fatality and disability rates

Case fatality and disability rates determine the various transition probabilities at nodes C, D, and E in Figure 1.

We assume inpatient and outpatient CAP case fatality rates (CFRs) of 9.2% and 0%, respectively.^43^ We derive bacteremia and meningitis CFRs using 2016 data from the ECDC *Surveillance Atlas of Infectious Diseases* for the above-mentioned six comparator countries.^38^ See the Methodological Appendix for further details.

We calculate the probability of temporary disability from inpatient CAP and bacteremia as one minus their respective CFRs. For meningitis, we assume five outcomes: death, persistent vegetative state (PVS), severe disability, moderate disability, and temporary disability. We derive probabilities of meningitis-related PVS, severe disability, and moderate disability from 1999–2002 data from van de Beek et al. (2004),^44^ with the residual probability representing the probability of temporary disability.

### 2.10. Indirect costs (productivity losses)

Indirect costs constitute the state utilities associated with the various PD-related Markov states in Figure 1. Categories of productivity loss include lost paid work and unpaid work in the form of housework, caregiving, and volunteering. The work may be that of the infected individual or of the infected individual’s formal and informal caregivers. The value of lost work equals time lost multiplied by the value per unit of time. We value paid work at the average pre-tax wage across all occupations (“average wage”), adjusted for the probability of employment. We value the infected individual’s unpaid work at its replacement cost (i.e., the cost of hiring someone to do the work) rather than at its opportunity cost (i.e., foregone earnings),^45^ the former of which we estimate using the average pre-tax wage in the elementary occupations (“unskilled wage”). We value time spent by the infected individual’s caregivers at its opportunity cost equal to foregone paid work. We assume the infected individual’s social circle is nationally representative, and that everyone in this circle is equally willing and able to miss work to provide care, and so value this foregone paid work at the average wage across all adults.

PD-related death, meningitis-related PVS, and severe disability each involves the loss of the infected individual’s lifetime productivity. Based on average months of survival in a study by the Multi-Society Task Force on PVS,^46^ we assume that an individual who enters a PVS stays in that state for four years and dies thereafter. According to van de Beek et al. (2004),^44^ severe disability involves such disability for the rest of the individual’s expected lifetime. Based on definitions of Glasgow Outcome Scale outcomes as reported in van de Beek et al. (2004),^44^ we assume that both PVS and severe disability necessitate long-term care – which we take to comprise nursing, domestic, and informal care – for the duration of disability.^47^ Nursing care is valued at the average pre-tax wage in the professional occupations (“professional wage”), which includes health work; domestic care is valued at the unskilled wage; and, because we assume that all potential informal caregivers are equally willing and able to miss work to provide care, informal care is valued at the average wage across all adults (see the Methodological Appendix for details).^48^

Based on a study by Weisfelt et al. (2006),^49^ we assume that moderate disability persists for two years (see the Methodological Appendix for details). After a study by Schmand et al. (2010),^50^ we assume that subsequent good recovery is lifelong. Based on definitions of Glasgow Outcome Scale outcomes as reported in van de Beek et al. (2004),^44^ we associate the two years of disability with an inability to do paid or unpaid work. However, these definitions also indicate that, unlike PVS and severe disability, moderate disability does not necessitate long-term care.

Temporary disability includes periods of no productivity (i.e., absenteeism) and reduced productivity upon returning to work (i.e., presenteeism), both of which we value at the average wage. Temporary disability also includes informal caregivers’ foregone paid work, which we value at the average wage across all adults.

We derive or construct earnings, employment, and general mortality risk data from Eurostat (see Table 1 for specific Eurostat sources by category); 2016 time use data from the Turkish Statistical Institute (TurkStat);^53^ duration of absenteeism, presenteeism, and days of caregiving required during temporary disability from Wyrwich et al. (2015)^54^ and Jiang et al. (2012)^55^ (see the Methodological Appendix for more information regarding how we construct these values); and population age structures from the World Population Prospects 2015.^56^ The lack of age-disaggregation of time use among the elderly in the data leads us to assume that time use is invariant across all ages 55 and older. This is implausible since rising disability with age should reduce work hours among the elderly, but it clearly does not bias our results. The reason is that we assign the average time use to each individual age, therefore we overstate the productivity of the oldest in the 55 and older age group, but understate that of the youngest in that age group by an exactly compensating amount.

**Table 1.** Sample input parameter values by age group

Parameter	18–19	20–24	25–29	30–34	35–39	40–44	45–49	50–54	55–59	60–64	65–69	70–74	75–79	80–84	85	Source
**Epidemiological parameters, elderly population**																
Vaccine-type incidence per 100,000 of																
Inpatient CAP	209.72	144.25	131.20	118.17	105.15	100.65	104.64	108.70	112.75	133.47	170.64	216.29	254.08	313.78	390.76	*
Outpatient CAP	871.31	599.32	545.10	490.94	436.88	418.18	434.74	451.60	468.44	554.53	708.96	898.63	1,055.60	1,303.64	1,623.47	*
Bacteremia	0.14	0.10	0.10	0.09	0.10	0.09	0.09	0.09	0.10	0.12	0.35	0.43	0.52	0.81	1.30	*
Meningitis	0.044	0.032	0.029	0.029	0.030	0.029	0.027	0.028	0.031	0.036	0.043	0.053	0.065	0.10	0.16	*
Case fatality rate																
Inpatient CAP	0.09	0.09	0.09	0.09	0.09	0.09	0.09	0.09	0.09	0.09	0.09	0.09	0.09	0.09	0.09	^43^
Outpatient CAP	0	0	0	0	0	0	0	0	0	0	0	0	0	0	0	^43^
Bacteremia	0.05	0.05	0.17	0.17	0.17	0.17	0.28	0.28	0.28	0.28	0.29	0.29	0.29	0.29	0.29	*
Meningitis	0.02	0.02	0.07	0.07	0.07	0.07	0.11	0.11	0.11	0.11	0.29	0.29	0.29	0.29	0.29	*
Probability of meningitis causing																
Persistent vegetative state	0.01	0.01	0.01	0.01	0.01	0.01	0.01	0.01	0.01	0.01	0.01	0.01	0.01	0.01	0.01	^44^
Severe disability	0.05	0.05	0.05	0.05	0.05	0.05	0.05	0.05	0.05	0.05	0.05	0.05	0.05	0.05	0.05	^44^
Moderate disability	0.14	0.14	0.14	0.14	0.14	0.14	0.14	0.14	0.14	0.14	0.14	0.14	0.14	0.14	0.14	^44^
Temporary disability	0.78	0.78	0.73	0.73	0.73	0.73	0.69	0.69	0.69	0.69	0.51	0.51	0.51	0.51	0.51	*
Economic parameters, elderly population**																
Annual earnings, all occupations	6,226.88	6,226.88	6,226.88	8,841.04	8,841.04	9,491.37	9,491.37	9,795.04	9,795.04	15,438.37	15,100.82	15,100.82	14,695.77	14,695.77	14,695.77	*
Hourly earnings, all occupations	2.40	2.40	2.40	3.33	3.33	3.54	3.54	3.68	3.68	5.20	5.20	5.20	5.20	5.20	5.20	^48^
Hourly earnings, elementary occupations	1.94	1.94	1.94	2.06	2.06	2.02	2.02	1.98	1.98	1.89	1.89	1.89	1.89	1.89	1.89	^48^
Average hourly earnings (aged 15+) in																
All occupations	3.10	3.10	3.10	3.10	3.10	3.10	3.10	3.10	3.10	3.10	3.10	3.10	3.10	3.10	3.10	^48^
Elementary occupations	2.01	2.01	2.01	2.01	2.01	2.01	2.01	2.01	2.01	2.01	2.01	2.01	2.01	2.01	2.01	^48^
Professional occupations	6.64	6.64	6.64	6.64	6.64	6.64	6.64	6.64	6.64	6.64	6.64	6.64	6.64	6.64	6.64	^48^
Employment rate	0.23	0.46	0.59	0.63	0.64	0.63	0.59	0.46	0.36	0.27	0.19	0.12	0.05	0.05	0.05	^51^
Average employment rate (aged 15+)	0.46	0.46	0.46	0.46	0.46	0.46	0.46	0.46	0.46	0.46	0.46	0.46	0.46	0.46	0.46	^51^
Weekly hours spent working	47.40	47.40	47.40	47.40	47.40	47.40	47.40	47.40	47.40	47.40	47.40	47.40	47.40	47.40	47.40	^52^
Daily minutes spent																
Doing housework	92.68	92.68	159.70	159.70	156.39	156.39	147.29	147.29	129.91	129.91	129.91	129.91	129.91	129.91	129.91	*
Volunteering	9.50	9.50	12.00	12.00	16.50	16.50	27.50	27.50	49.00	49.00	49.00	49.00	49.00	49.00	49.00	*
Caregiving	19.32	19.32	33.30	33.30	32.61	32.61	30.71	30.71	27.09	27.09	27.09	27.09	27.09	27.09	27.09	*
For long-term care, monthly hours of																
Informal care	37.87	37.87	37.87	37.87	37.87	37.87	37.87	37.87	37.87	37.87	37.87	37.87	37.87	37.87	37.87	^47^
Nursing care	2.66	2.66	2.66	2.66	2.66	2.66	2.66	2.66	2.66	2.66	2.66	2.66	2.66	2.66	2.66	^47^
Paid domestic help	9.30	9.30	9.30	9.30	9.30	9.30	9.30	9.30	9.30	9.30	9.30	9.30	9.30	9.30	9.30	^47^
**Parameters for comorbid population**																
Prevalence of comorbidities	0.41	0.41	0.41	0.41	0.41	0.42	0.42	0.43	0.43	0.46	0.47	0.48	0.60	0.62	0.62	*
Earnings scale factor	0.89	0.89	0.89	0.89	0.89	0.89	0.89	0.89	0.89	0.89	0.89	0.89	0.89	0.89	0.89	*
Background mortality rate scale factor	5.48	4.84	4.84	4.40	4.40	2.51	2.51	2.16	2.16	1.84	1.84	1.61	1.61	0.94	0.94	*
Incidence, bacteremia/meningitis scale factor	2.25	2.25	2.25	2.26	2.26	2.23	2.23	2.11	2.11	2.04	1.91	1.87	1.51	1.47	1.26	*
Incidence, outpatient CAP scale factor	1.54	1.54	1.54	1.54	1.54	1.53	1.53	1.52	1.52	1.48	1.47	1.45	1.39	1.36	1.36	*
Incidence, inpatient CAP scale factor	2.16	2.16	2.16	2.16	2.16	2.15	2.15	2.10	2.10	2.03	1.97	1.93	1.51	1.48	1.39	*

* See the Methodological Appendix; abbreviation: CAP = community-acquired pneumonia; ** All monetary values are in 2014 Euros.

### 2.11. Comorbid subpopulation-specific parameters

Our comorbid subpopulation consists of adults who enter the model aged 18–64 with at least one of the following comorbidities: chronic obstructive pulmonary disease, asthma, diabetes, chronic kidney failure, chronic heart disease, and cancer. Prevalence rates of comorbidities come from Turkish data sources.^57–62^ We construct parameters for the comorbid subpopulation by scaling parameter values that apply to Turkey’s general population to account for the impact of comorbidities on those parameter values. This involves scaling up non-PD-related mortality rates, incidence rates, and parameters related to length of hospital stay for inpatient and outpatient CAP (specifically, days before full productivity is reached for temporary disability, the hours per month of long-term care, and days of short-term care needed). It involves scaling down earnings among all comorbid adults, and vaccine efficacy among those at high risk for pneumococcal disease. The scale factor applied to a particular parameter value depends on non-Turkish data on the ratio of that parameter value in comorbid adults to its value in the general population. Note that CFRs discussed in Section 2.8 do not require rescaling since these already apply to comorbid populations along with the elderly.

See the Methodological Appendix for further detail and data.

### 2.12. Vaccination benefits and rate of return

Vaccination benefits equal the indirect costs averted by vaccination, that is, the difference between a vaccinated and unvaccinated cohort in the expected present discounted value of indirect costs of PD. These benefits represent a productivity-based breakeven level for vaccine costs: any costs in excess of these benefits would result in a negative productivity-based rate of return (RoR). These benefits reflect age- and comorbidity-status-specific benefits averaged using age- and comorbidity-status-specific population weights. We construct these weights using population data from the World Population Prospects 2015^56^ and comorbidity prevalence data discussed in Section 2.10 (see also the Methodological Appendix).

The RoR is the ratio of vaccination benefits to vaccine procurement and administration costs, minus 1, multiplied by 100. Since such costs are not in the public domain, we present hypothetical RoRs over a range of hypothetical costs.

### 2.13. Discounting and economic growth

We discount costs and benefits at a rate of 3% annually, which is typical in the economic evaluation literature, and has been recommended by the Second Panel^2^ and used in a prior evaluation of PPV23 Adult in Turkey by Akin et al. (2011).^16^ Note that all vaccination occurs in the base period (there is a one-time vaccination opportunity), so their costs do not require discounting. Based on average economic growth rates over 20 years in Turkey and nine comparator countries, we assume that its economy, and therefore wages, will grow 2.61% annually. Since benefits are multiplicative in wages, (see the Methodological Appendix for benefit formulas), a benefit discount rate of 3% and a wage growth rate of 2.61% implies an effective discount rate of 0.39%.

### 2.14. Currency conversion

To convert monetary input parameters – which are in 2014 euros – to 2017 United States (U.S.) dollars, we first convert 2014 euros to 2017 euros using the ratio of Turkey’s 2017 GDP deflator to its 2014 value, which equals 1.29.^63^ We then convert these 2017 euros to 2017 U.S. dollars using the average monthly exchange rate from January to December 2017 from the U.S. Federal Reserve Economic Data, which equals 1.13.^64^ Therefore, the final conversion factor by which we multiply 2014 euro values to yield 2017 U.S. dollar values is 1.29 × 1.13 = 1.46.

### 2.15. Scenario and sensitivity analyses

Our base case vaccine efficacies (see Section 2.7) allow baseline vaccine efficacy to decline with age to account for immunosenescence among those aged 50 and up. However, given the prominence of the CAPITA results of vaccine efficacy of 45.0% for inpatient and outpatient CAP and 75.0% for bacteremia and meningitis, we perform a scenario analysis using these headline numbers as baseline vaccine efficacies for all ages within the program population. We call this our “age-invariant vaccine efficacy scenario.”

We also assess the sensitivity of vaccine benefits to the magnitude of herd effects from PCV13 Pediatric by raising the assumed incidence rate ratio (i.e., the ratio of incidence rates after herd effects to those before herd effects) from our baseline value of 0.12 up to 1.00 (i.e., an assumption of no herd effects) using intervals of 0.04 (i.e., using incidence rate ratios of 0.12, 0.16, 0.20, …, 0.96, and 1.00). We do so for both the base case and the age-invariant baseline vaccine efficacy scenarios.

In our last sensitivity analysis, we derive vaccine-type incidence rates using two alternative estimates of PCV13 vaccine-type coverage rates, both of which we obtain from Ceyhan et al. (2016).^65^ The first alternative coverage rate is 56.6% based on the Ceyhan et al. estimate for Hungary, and the second is 85.5% based on the study’s estimate for Turkey.

## 3. Results

Our results involve two parts: (i) the indirect costs per treatment episode of PD; and (ii) vaccination benefits and RoR.

### 3.1. Indirect costs

Table 2 presents indirect costs per treatment episode of PD for the elderly and comorbid subpopulations separately, averaged across ages within each subpopulation using weights proportional to incidence rates and population size.

**Table 2.** Average indirect costs per treatment episode (2017 USD) and percent of 2017 per capita GDP by manifestation and subpopulation

PD manifestation	Elderly subpopulation 65–85 years	Comorbid subpopulation 18–64 years
**Inpatient CAP**		
Average indirect costs	$4,449.42	$23,385.72
Percent of 2017 per capita GDP	42.0%	220.6%
**Outpatient CAP**		
Average indirect costs	$250.12	$325.87
Percent of 2017 per capita GDP	2.4%	3.1%
**Bacteremia**		
Average indirect costs	$12,724.00	$31,865.32
Percent of 2017 per capita GDP	120.0%	300.6%
**Meningitis**		
Average indirect costs	$17,219.52	$34,392.25
Percent of 2017 per capita GDP	162.4%	324.4%

The average indirect costs of each manifestation are higher in the comorbid subpopulation than in the elderly subpopulation. The costs are about 5.26 times higher for inpatient CAP, 1.30 times higher for outpatient CAP, 2.50 times higher for bacteremia, and nearly twice as high for meningitis. With the exception of outpatient CAP, the average indirect costs per treatment episode of each manifestation either exceed or are a substantial fraction of Turkey’s 2017 per capita GDP of $10,602.17.^66^ To help assess these magnitudes’ plausibility, we note that these indirect costs are not limited to those foregone during or because of treatment (such as earnings lost from inpatient stays). They include broader indirect costs like those from premature mortality associated with the episode.

In terms of Figure 1, these manifestation-specific indirect costs per treatment episode constitute the expected cycle utilities conditional on contracting inpatient CAP, outpatient CAP, bacteremia, and meningitis, respectively, after node B. Figure 1 shows that for each manifestation (e.g., inpatient CAP), indirect costs are the weighted average across the outcomes under that manifestation (e.g., death and temporary disability in the case of inpatient CAP) of the outcome-specific indirect costs (e.g., the indirect costs of death from inpatient CAP and of temporary disability from inpatient CAP), where the weights equal the probabilities of the outcomes conditional on infection (e.g., the probability of death from inpatient CAP infection equals the CFR and the probability of temporary disability from inpatient CAP is 1 minus the CFR). Table 3 disaggregates manifestation-specific indirect costs into the probabilities and indirect costs of the various outcomes possible under each manifestation. It shows these separately for the elderly and comorbid subpopulations.

**Table 3.** Average indirect costs per treatment episode (2017 USD) by PD manifestation, disease outcome and subpopulation

	Death		TD		PVS		SD		MD	
	Prob.	IC	Prob.	IC	Prob.	IC	Prob.	IC	Prob.	IC
**Inpatient CAP**										
Elderly	0.09	$44,707.71	0.91	$370.39	-	-	-	-	-	-
Comorbid	0.09	$248,957.16	0.91	$530.46	-	-	-	-	-	-
**Outpatient CAP**										
Elderly	-	-	1.00	$250.12	-	-	-	-	-	-
Comorbid	-	-	1.00	$325.87	-	-	-	-	-	-
**Bacteremia**										
Elderly	0.29	$42,651.37	0.71	$446.06	-	-	-	-	-	-
Comorbid	0.22	$198,973.50	0.78	$556.03	-	-	-	-	-	-
**Meningitis**										
Elderly	0.29	$42,651.37	0.51	$446.06	0.01	$46,856.68	0.05	$51,093.16	0.14	$10,728.54
Comorbid	0.11	$242,031.82	0.69	$589.10	0.01	$246,759.71	0.05	$268,885.70	0.14	$16,604.79

Abbreviations: CAP = community-acquired pneumonia; Prob. = probability; IC = indirect costs; TD = temporary disability; PVS = persistent vegetative state; SD = severe disability; MD = moderate disability.

Table 3 shows that death and long-term meningitis sequelae are generally at least two orders of magnitude more costly than temporary disability.

The indirect costs of death at a given age are the same across all manifestations since the magnitude of the productivity loss from death does not depend on the cause of death. Table 4 disaggregates the indirect costs of death at sample ages by the source of productivity losses.

**Table 4.** Indirect costs of death and its components at sample ages (2017 USD)

Age	Indirect costs of death	Value of lifetime paid work lost	Value of lifetime housework lost	Value of lifetime caregiving lost	Value of lifetime volunteering lost
**Comorbid subpopulation**					
20	$406,916.87	$247,306.57	$113,891.41	$23,745.30	$21,973.59
25	$390,629.81	$236,416.70	$109,515.35	$22,832.93	$21,864.84
30	$361,079.55	$219,869.95	$99,042.01	$20,649.34	$21,518.26
35	$317,163.42	$190,346.45	$87,466.06	$18,235.86	$21,115.05
40	$272,491.98	$160,026.76	$76,200.24	$15,887.04	$20,377.93
45	$223,802.39	$126,119.12	$64,609.60	$13,470.50	$19,603.17
50	$178,421.36	$95,069.74	$54,080.23	$11,275.22	$17,996.16
55	$140,278.93	$70,477.34	$44,020.45	$9,177.85	$16,603.29
60	$109,777.97	$52,714.98	$35,986.83	$7,502.92	$13,573.23
65	$76,178.98	$30,694.15	$28,685.05	$5,980.57	$10,819.21
70	$50,546.39	$15,630.26	$22,019.90	$4,590.94	$8,305.30
75	$31,319.77	$6,431.84	$15,695.60	$3,272.39	$5,919.94
80	$19,634.50	$4,032.15	$9,839.64	$2,051.47	$3,711.24
85	$2,181.91	$448.08	$1,093.44	$227.97	$412.42
**Elderly subpopulation**					
65	$89,776.42	$37,640.02	$32,879.87	$6,855.15	$12,401.38
70	$58,548.12	$19,167.55	$24,835.40	$5,177.95	$9,367.22
75	$35,287.01	$7,947.16	$17,241.91	$3,594.78	$6,503.17
80	$19,892.18	$4,480.01	$9,719.70	$2,026.47	$3,666.00
85	$2,237.82	$503.99	$1,093.44	$227.97	$412.42

Table 4 shows that the indirect costs of death (and, as a result, the indirect costs per treatment episode for manifestations other than outpatient CAP) decline with age. It shows that for ages below 70 years, the largest productivity losses from death come from lost paid work, then from lost housework, then from either lost caregiving or volunteering. Although we value unpaid work less per unit of time than paid work, the burdens of lost unpaid work exceed those of lost paid work at ages 70 and older. This is because, on average, older adults devote much more of their time to unpaid work than to paid work (a 70-year-old, for example, can look forward to very little future paid work, but up to 16 more years of unpaid work).

The indirect costs per treatment episode of inpatient CAP in Table 2 are much lower than those of bacteremia and meningitis (recall that these indirect costs are weighted averages of the indirect costs of death and disability associated with that episode). However, as Table 1 shows, the incidence of inpatient CAP is much higher than that of either bacteremia or meningitis. Thus, the expected indirect costs of inpatient CAP (i.e., the indirect costs of a treatment episode multiplied by the probability of such episode, where the probability equals the incidence rate) are much higher than those of either bacteremia or meningitis. Across the comorbid and elderly populations, the expected indirect costs of a treatment episode are €2,232.88 for inpatient CAP, €3.73 for bacteremia, and €0.92 for meningitis. Thus, the expected indirect costs associated with inpatient CAP are over two orders of magnitude larger than those associated with either bacteremia or meningitis. Indeed, the expected indirect costs of outpatient CAP across the comorbid and elderly populations are €119.63, which is over an order of magnitude larger than those of bacteremia and meningitis.

### 3.2. Vaccination benefits and rate of return

**Figure 2.**
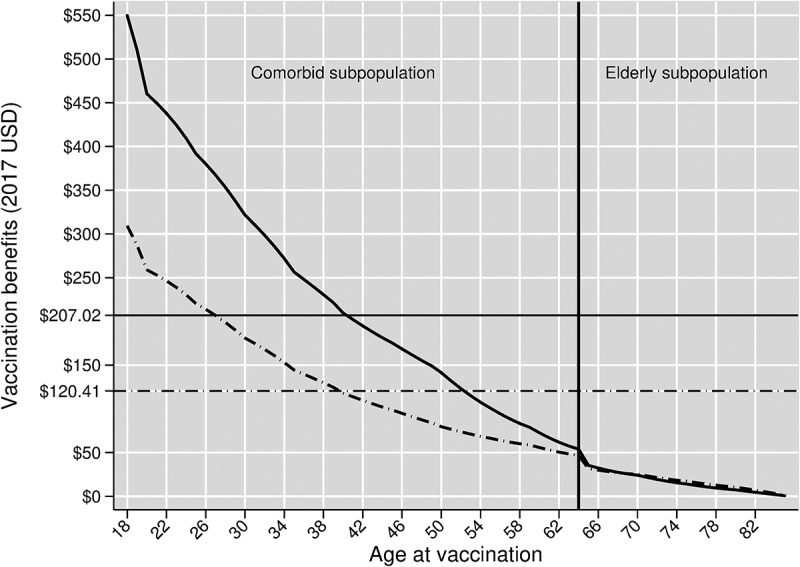
Benefits of PCV13 Adult by age at vaccination, assuming herd effects from pediatric vaccination. The solid curve shows vaccine benefits in our base case scenario, which assumes age-varying baseline vaccine efficacies. The dashed curve shows vaccine benefits in our scenario analysis that replaces our base case vaccine efficacies with age-invariant baseline vaccine efficacies

Vaccination benefits can be differentiated by age and comorbidity status. The solid curve with the downward trajectory in Figure 2 shows these vaccine benefits in our base case vaccine efficacy scenario. The vertical line in Figure 2 separates the vaccine benefits curve into two segments: the left of the line represents vaccination benefits in the comorbid subpopulation, and the right of the line represents vaccination benefits in the elderly subpopulation.

These per-person vaccination benefits are $550 for comorbid adults aged 18 at vaccination and decline to a little above $50 for comorbid adults aged 64. These benefits are $35 for elderly aged 65 and decline to nearly zero at higher ages. Among the manifestations and outcomes, the averted indirect costs of death from inpatient CAP account for the largest share of these vaccine benefits because of: (i) inpatient CAP’s much higher incidence rates relative to those of either bacteremia or meningitis; and (ii) the very high indirect costs of death relative to the indirect costs of temporary disability. Vaccination benefits fall with age due to declining lifetime potential productivity. In the elderly subpopulation, benefits decline in later years largely because of our model’s termination after age 85, which given a maximum of 15 years of vaccine efficacy, truncates the benefit stream of those vaccinated after age 71.

The comorbid subpopulation receives considerably greater vaccine benefits than the elderly subpopulation largely because the former has higher indirect costs per inpatient CAP treatment episode. This stems from comorbid adults being younger and therefore having longer productive lifespans to protect with vaccination.

Averaging vaccination benefits across the entire program population yields a program-wide per-person vaccination benefit of $207.02. This program-wide per-person benefit mainly reflects the benefits from vaccinating the younger comorbid subpopulation rather than the elderly subpopulation because the former group is almost four times the size of the latter in the Turkish population.

**Figure 3.**
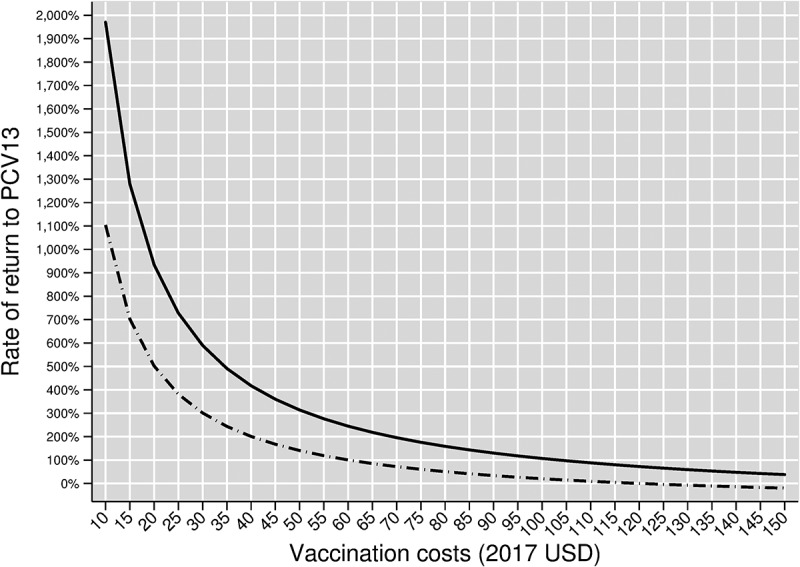
Rate of return to the entire Turkish PCV13 Adult program by vaccination cost, assuming herd effects from pediatric vaccination. The solid curve shows rates of return in our base case scenario, which assumes age-varying baseline vaccine efficacies. The dashed curve shows rates of return in our scenario analysis that replaces our base case vaccine efficacies with age-invariant baseline vaccine efficacies

Since vaccination costs in Turkey are not in the public domain, we present hypothetical rates of return (RoRs) for a range of vaccination costs between $10 and $150 in Figure 3. The relation between hypothetical vaccination cost (on the horizontal axis) and hypothetical RoR (on the vertical axis) for our base case vaccine efficacy scenario is shown in the solid curve.

Breakeven vaccination costs – that is, vaccination costs yielding an RoR of zero – by definition equal the program-wide vaccination benefits of $207.02. The hypothetical RoR is positive at hypothetical vaccination costs below this breakeven level and negative at hypothetical costs above it. At hypothetical vaccination costs of $10, the RoR would be nearly 2,000%. At vaccination costs of: $20, the RoR would be slightly over 900%; $50, the RoR would be slightly over 300%; $80, the RoR would be nearly 160%; $100, the RoR would be slightly over 100%; and $150, the RoR would be 38%.

### 3.3. Scenario and sensitivity analyses

Our first scenario analysis replaces our baseline vaccine efficacies with age-invariant baseline vaccine efficacies. This results in the dashed and downward sloping age-varying vaccine benefit curve in Figure 2, and the dashed curve linking hypothetical vaccination costs and RoRs in Figure 3. Vaccination benefits averaged across the program cohort under this age-invariant vaccine efficacy scenario are $120.41.

The most important effect of this scenario analysis is to reduce vaccination benefits and RoRs. This is because the age-invariant baseline vaccine efficacies in this scenario come from the CAPITA trial whose sample consists of elderly aged 65 and up. Extrapolating baseline vaccine efficacy from this age group to all younger ages therefore involves extrapolating the immunosenescence within this age group to the entire and often much younger program subpopulation. In contrast, our base case vaccine efficacy scenario allows vaccine efficacy to be higher in the non-elderly than in the elderly, reflecting the reduced role of immunosenescence in the former.

**Figure 4.**
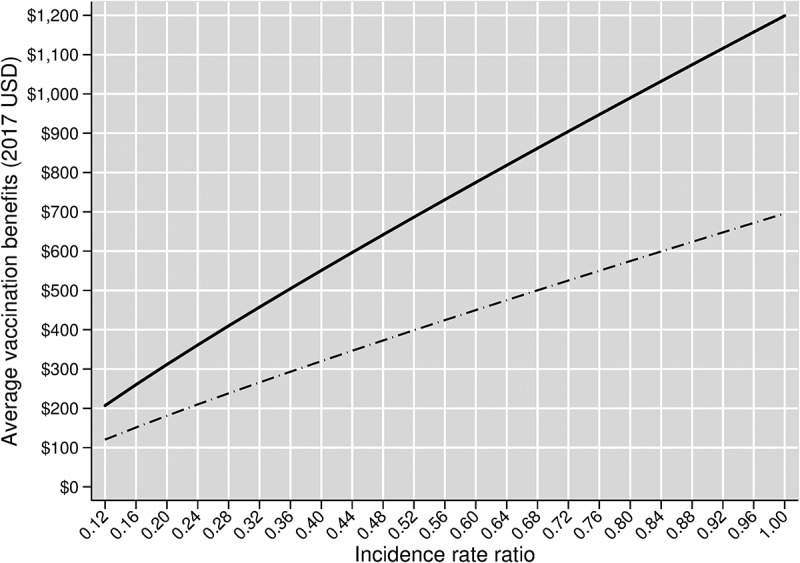
Average vaccination benefits by incidence rate ratio over the entire Turkish PCV13 Adult program. The solid curve shows vaccine benefits in our base case scenario, which assumes age-varying baseline vaccine efficacies. The dashed curve shows vaccine benefits in our scenario analysis that replaces our base case vaccine efficacies with age-invariant baseline vaccine efficacies

Computing program-wide vaccination benefits for intermediate incidence rate ratios for herd effects from PCV13 Pediatric between 0.12 (which we use for our base case analysis) and 1.00 (which implies no herd effects) yields the results in Figure 4.

Figure 4 shows that program-wide per person vaccination benefits rise quickly with weakening herd effects. For example, assuming no herd effects, average benefits are approximately 5.8 times greater ($1,199/$207). This suggests that if herd effects were weaker than our base case assumes, then the benefits of vaccinating adults would quickly become massive.

Figure 4 also shows a substantial difference in average vaccination benefits between our base case and age-invariant vaccine efficacy scenarios. As discussed above with respect to the results illustrated in Figures 2 and Figures 3, this result is explained by the considerably higher baseline vaccine efficacy rates used in the base case compared to those used in the age-invariant efficacy scenario for ages 18–64.

Deriving vaccine-type incidence rates using PCV13 coverage of 56.6% (Hungary-specific estimates) and 85.5% (Turkey-specific estimates) yields program-wide vaccination benefits of $172.33 and $260.26, respectively.

## 4. Discussion

In contrast to much of the economic evaluation literature on PCV13 Adult, we use a (limited) societal perspective CBA using a human capital approach and incorporating unpaid work. And in contrast to that literature, we find large vaccination benefits even in the presence of PPV23 and herd effects from PCV13 pediatric. The broadening of perspective from a health payer’s perspective to a societal perspective raises the benefits of vaccination by $207.02. Our more specific empirical conclusions are as follows. First, PD’s indirect costs in Turkey are large relative to benchmarks like Turkish per capita GDP. Economic theory and welfare economics affirm the value of unpaid work and our indirect costs results prove their quantitative importance in assessing disease burdens and valuing the health of older adults and the elderly. Second, the indirect costs per treatment episode of PD are largely accounted for by the costs of death rather than the costs of temporary disability, by the costs of inpatient CAP rather than the costs of other manifestations, and by the value of market productivity rather than nonmarket productivity among those younger than age 70. Third, vaccination benefits are larger in the younger comorbid subpopulation than in the elderly subpopulation because of the longer productive lifespans that can be protected with vaccination. Fourth, the productivity-based RoR to Turkey’s PCV13 Adult program is sizeable for a wide range of hypothetical vaccination costs. For example, Turkey-specific estimates of the return to investments in schooling are below 20%.^67^ Estimates of the return to the Global Alliance for Vaccines and Immunization’s immunization program are between 12–18%,^68^ and the return to the Expanded Programme on Immunization vaccination in the Philippines is estimated at 21%.^69^ In contrast, at a hypothetical vaccination cost of $50, the RoR to PCV13 Adult in the program population is approximately 300%.

We emphasize that these benefit and RoR calculations reflect productivity benefits alone. They exclude other elements of value, most importantly the intrinsic value of health itself, but also averted direct costs and other socio-economic benefits such as the effects on leisure.

### 4.1. Policy implications

The fundamental principle animating our analysis is that policymakers should evaluate vaccines in light of their full socio-economic benefits over and above narrow QALY gains and avoided medical care costs. Failing to account for vaccines’ full value – including their full productivity value – risks substantial undervaluation. The RoRs we found at a range of hypothetical vaccination costs imply that on productivity grounds alone PCV13 Adult is likely to be a sufficiently attractive investment relative to other publicly-financed health and non-health expenditures.

Another policy implication is the considerable benefit to vaccinating non-elderly comorbid adults, a result of their high prospective lifetime productivity and substantial reductions in mortality risk from vaccination.

### 4.2. Limitations

Our analysis omits health, direct costs, consumption, leisure, financial risk protection, social equity, complete fiscal and macroeconomic impacts, and other broad benefits resulting from PCV13 Adult vaccination, consideration of which we expect to raise the RoR. On the other hand, we also omit consideration of aging-related health care and social care, which would lower the RoR. Given the current state of the literature, it is not clear whether the omitted positive factors dominate the omitted negative ones or vice versa. But this suggests that our RoRs do not obviously overstate or understate those that would result from a full societal perspective, and so are at least somewhat informative of such results.

Data are limited in Turkey, compelling the use of many assumptions and imputations. Real-world data on long-term vaccine effectiveness, herd effects from PCV13 Pediatric (the magnitude of which, in turn, depends on carriage prevalence in children and adults and the impact of pediatric vaccination on such carriage),^72–74^ and possible herd or serotype replacement effects from adult vaccination are incomplete. Estimates of inpatient CAP and IPD incidence depend on hospital discharge records, which suffer from underreporting,^72–74^ though this makes our estimates conservative. Data scarcity compelled us to calibrate our calculations with data from other countries (e.g., the United States with respect to the productivity losses associated with temporary disability^54^). Whether these data limitations combine to produce more or less conservative estimates is unclear.

### 4.3. Implications for future work

Our analysis improves on the existing empirical literature by measuring the indirect costs of adult PD and the productivity-based social RoR to PCV13 Adult, which integrates death and disability over a lifetime, invasive and noninvasive PD, and paid and unpaid work. We argue that such analysis is firmly grounded in CBA from a societal perspective and defend against ethical concerns.

There are some obvious directions for future work. The first is to expand our empirical analysis and theoretical framework to encompass leisure in addition to productivity. Such an analysis would show how producible goods and services and leisure jointly determine well-being and would also allow us to quantify in monetary terms the contribution that PCV13 Adult can make toward the European Commission’s^75^ and World Health Organization’s^76^ policy goal of promoting Healthy Aging.

The second direction for future work is to investigate more fully the relationship between PD and chronic disease comorbidities. Our analysis of Danish diabetics and Turkish comorbid adults suggests that the benefits of vaccinating adults and elderly with chronic disease against PD may be especially high.^77^ In the language of economic theory, this may be because chronic disease status is a conveniently observable marker for *targeting* PCV13 to adults for whom vaccination has especially high benefits. Expanding this work to consider more explicitly the interactions among PD, other chronic and infectious diseases, long-term and permanent disability, and early retirement from the workforce may be valuable.

A third direction for future work could be to move beyond a consideration of PD and PCV13 adult in isolation, toward evaluating its role within a *life course vaccination* framework. Such a framework would simultaneously consider all vaccine-preventable diseases and their associated comorbidities and vaccines and attempt to design a lifetime vaccine schedule that is optimal in the sense of maximizing some social objective like QALYs per euro spent or (better, in our judgment) the social rate of return.

Finally, a fourth direction could be to evaluate PCV13 Adult’s contribution to reducing the risks of antimicrobial resistance (AMR). Some forms of *Streptococcus pneumoniae* have become resistant to antibiotics, and PCV13 protects against some of these forms (e.g., serotype 19A).^78^ Such analysis could be especially valuable given the prominence of AMR among global health priorities and the dearth of empirical estimates of the value of vaccines in preventing AMR.

## Supplementary Material

Supplemental Material
