# Supplementary material for: Indirect costs of adult pneumococcal disease and the productivity-based rate of return to the 13-valent pneumococcal conjugate vaccine for adults in Turkey
Source: Hum Vaccin Immunother. 2020 Jan 29;16(8):1923–36. doi: 10.1080/21645515.2019.1708668 (PMC7482724; doi:10.1080/21645515.2019.1708668)
Supplement: Supplemental Material [file KHVI_A_1708668_SM4591.docx]

**Indirect costs of adult pneumococcal disease and the productivity-based rate of return to the 13-valent pneumococcal conjugate vaccine for adults in Turkey**

**Methodological Appendix**

**Table of Contents**

[A1. Introduction 4](#_Toc22218747)

[A2. Markov model 4](#_Toc22218748)

[A3. Transition probabilities 5](#_Toc22218749)

[**A3.1. Prevalence of comorbidity, non-comorbidity, and various risk groups within the general population** 6](#_Toc22218750)

[**A3.1.1. Prevalence of comorbidities** 6](#_Toc22218751)

[**A3.1.2. Prevalence of non-comorbid adults** 8](#_Toc22218752)

[**A3.1.3. Prevalence of low, medium, and high PD-related risks** 9](#_Toc22218753)

[**A3.1.4. Risk group prevalence rates** 10](#_Toc22218754)

[**A3.2. Incidence rates, herd effects, and vaccine efficacy** 12](#_Toc22218755)

[**A3.2.1. Vaccine-type incidence rates in the absence of vaccination** 13](#_Toc22218756)

[**A3.2.1.i. Community-acquired pneumonia vaccine-type incidence rates for the general population** 13](#_Toc22218757)

[**A3.2.1.ii. Invasive pneumococcal disease vaccine-type incidence rates for the general population** 14](#_Toc22218758)

[**A3.2.2. Herd effects from PCV13 Pediatric** 22](#_Toc22218759)

[**A3.2.3. Vaccine-type community-acquired pneumonia and invasive pneumococcal disease incidence rates for the comorbid subpopulation** 23](#_Toc22218760)

[**A3.2.4. Vaccine-type vaccine efficacy** 24](#_Toc22218761)

[**A3.2.4.i. Baseline vaccine-type vaccine efficacy in the general population, base case scenario** 24](#_Toc22218762)

[**A3.2.4.ii. Baseline vaccine-type vaccine efficacy in the general population, age-invariant scenario** 25](#_Toc22218763)

[**A3.2.4.iii. Waning** 25](#_Toc22218764)

[**A3.2.4.iv. Baseline vaccine-type vaccine efficacy in the comorbid subpopulation** 25](#_Toc22218765)

[**A3.2.5. Vaccine-type incidence rates for the vaccinated** 26](#_Toc22218766)

[**A3.3. Case fatality rates and probabilities of disability** 27](#_Toc22218767)

[**A3.3.1. Community-acquired pneumonia case fatality rates in the general population and comorbid subpopulation** 27](#_Toc22218768)

[**A3.3.2. Invasive pneumococcal disease case fatality rates in the general population and comorbid subpopulation** 27](#_Toc22218769)

[**A3.3.3. Probabilities of disability for the general population and the comorbid subpopulation** 28](#_Toc22218770)

[**A3.4. Probability of a non-PD death** 29](#_Toc22218771)

[**A3.4.1. Mortality risks in the general population** 29](#_Toc22218772)

[**A3.4.2. Mortality risks in the comorbid subpopulation** 30](#_Toc22218773)

[A4. State utilities as indirect costs 31](#_Toc22218774)

[**A4.1. Economic data inputs** 33](#_Toc22218775)

[**A4.1.1. Employment rates** 33](#_Toc22218776)

[**A4.1.2. Earnings** 33](#_Toc22218777)

[**A4.1.2.i. Hourly earnings in the general population** 35](#_Toc22218778)

[**A4.1.2.ii. Annual earnings for all occupations in the general population** 36](#_Toc22218779)

[**A4.1.2.iii. Productivity for the comorbid subpopulation** 39](#_Toc22218780)

[**A4.1.3. Time use** 40](#_Toc22218781)

[**A4.1.3.i Market time use** 40](#_Toc22218782)

[**A4.1.3.ii. Non-market time use** 41](#_Toc22218783)

[**A4.2. Indirect costs of death** 41](#_Toc22218784)

[**A4.2.1. Life table variables and formulas for the general population and the comorbid subpopulation** 41](#_Toc22218785)

[**A4.2.2. Expected value of lifetime earnings** 43](#_Toc22218786)

[**A4.2.3. Growth rate of per capita GDP** 43](#_Toc22218787)

[**A4.2.4. Expected values of lifetime housework, caregiving, and volunteering** 45](#_Toc22218788)

[**A4.2.5. Formula for the indirect costs of death** 46](#_Toc22218789)

[**A4.3. Indirect costs of meningitis-related persistent vegetative state, severe disability and moderate disability** 46](#_Toc22218790)

[**A4.3.1. Indirect costs of a persistent vegetative state** 47](#_Toc22218791)

[**A4.3.2. Indirect costs of severe disability** 48](#_Toc22218792)

[**A4.3.3. Indirect costs of moderate disability** 50](#_Toc22218793)

[**A4.3.3.i. Formulas for lifetime production lost** 50](#_Toc22218794)

[**A4.3.3.ii. Period of informal caregiving** 51](#_Toc22218795)

[**A4.3.3.iii. Formula for the indirect costs of moderate disability** 52](#_Toc22218796)

[**A4.4. Indirect costs of temporary disability** 52](#_Toc22218797)

[**A4.4.1. Period of full incapacity** 52](#_Toc22218798)

[**A4.4.2. Indirect costs during the period of full incapacity** 53](#_Toc22218799)

[**A4.4.3. Period of partial capacity** 54](#_Toc22218800)

[**A4.4.4. Indirect costs during the period of partial capacity** 55](#_Toc22218801)

[**A4.4.5. Value of informal caregiving** 55](#_Toc22218802)

[**A4.4.6. Formula for the indirect costs of temporary disability** 56](#_Toc22218803)

[**A4.4.7. Indirect costs of temporary disability for the comorbid subpopulation** 56](#_Toc22218804)

[A5. Vaccination benefits and rates of return 57](#_Toc22218805)

[**A5.1. Markov cycle utilities** 57](#_Toc22218806)

[**A5.2. Cumulative utility** 58](#_Toc22218807)

[**A5.2.1. Non-vaccinated cohort** 58](#_Toc22218808)

[**A5.2.2. Vaccinated cohort** 59](#_Toc22218809)

[**A5.3. Formula for rates of return** 59](#_Toc22218810)

[A6. Other topics 60](#_Toc22218811)

[**A6.1. Sensitivity analyses** 60](#_Toc22218812)

[**A6.2. Averaging indirect costs across ages** 61](#_Toc22218813)

[**A6.3. Expected indirect costs** 61](#_Toc22218814)

[**A6.4. Currency conversion** 62](#_Toc22218815)

[References 62](#_Toc22218816)

**Indirect costs of adult pneumococcal disease and the productivity-based rate of return to the 13-valent pneumococcal conjugate vaccine for adults in Turkey**

**Methodological Appendix**

# A1. Introduction

This methodological appendix describes the Markov model in Section A2. Implementing this model requires specifying the model’s transition probabilities and state utilities. We discuss the transition probabilities, which are determined by various epidemiological and demographic variables, in Section A3. Section A4 discusses the calculation of state utilities, which consist of the indirect costs associated with a Markov state. These indirect costs depend on economic data, time use data, and on epidemiological and demographic data, which we discuss in Section A4.1. Section A5 discusses the calculation of vaccination benefits and rates of return. Section A6 discusses other topics, which include our sensitivity analyses, our methodology for calculating the summary statistics that we present in the article, and our methodology for converting 2014 euros to 2017 U.S. dollars.

# A2. Markov model

We use a Markov model of disease progression, with one-year cycles represented by the Markov-cycle tree in Figure 1 in the article. We take the base year of our model to be the calendar year 2015, which we denote $t=0$. Our single cohort consists of Turkish adults who are eligible to receive public reimbursement for the 13-valent pneumococcal conjugate vaccine (PCV13) within the Turkish Ministry of Health’s National Immunization Program. We call this cohort the “program population.” It consists of two subpopulations: all adults aged 65 years and older during the base year (the “elderly subpopulation”), and adults aged 18–64 years during the base year with one or more of the following six comorbidities: chronic obstructive pulmonary disease, asthma, diabetes, chronic kidney failure, chronic heart disease, and cancer (the “comorbid subpopulation”).^[[1]](#footnote-1)^

The single cohort faces a one-time opportunity to vaccinate at $t=0$, after which it enters an initial Markov state at $t=0$ corresponding to the “Uninfected by PD” state, where “PD” denotes pneumococcal disease, and then transitions through the various states of the model until death. Data scarcity leads us to conservatively assume that no individual survives longer than 85 years of age. We denote age by $x\in[18,85]$.

The longest any individual stays in the model is 68 years (i.e., a comorbid adult who is 18 years old in the base year and who lives until age 85). Accordingly, the cycles are between the calendar years 2015–2082, inclusive, and are indexed by $t \in[0,67]$. The Markov states are the branches of the M nodes. Transition probabilities and state utilities can vary with respect to both $t$ (calendar time) and $x$ (age). Absorbing states terminate in left-pointed triangles.

Our model allows at most one treatment episode (i.e., inpatient or outpatient medical visit) per person per cycle. We set the probabilities of developing the various manifestations of pneumococcal disease (PD) equal to the relevant incidence rates (i.e., the number of infections per 100,000 person years). Thus, we implicitly assume only one treatment episode per infection, which is conservative since infections may involve multiple treatments.

Each Markov state is associated with a “state utility,” which is equal to the indirect costs of a treatment episode of adult PD associated with that state. Each cycle is associated with a “cycle utility,” which is equal to the expected value of the state utilities within that cycle, where the expectation is taken with respect to all states that are possible within the cycle and the transition probabilities of these states. A cohort entering the Markov model faces a “cumulative utility,” which is equal to the expected present discounted value (PDV) of the sum of cycle utilities over that cohort’s lifetime, where the expectation is taken with respect to the likelihood of surviving to a particular cycle.

We run the Markov model twice: the first run assumes that the cohort chooses not to be vaccinated, and the second assumes that the cohort chooses to be vaccinated. Vaccination’s sole impact on the Markov model is that it reduces the incidence of infection. Thus, though vaccinated cohorts face the same state utilities as unvaccinated cohorts, they will have smaller probabilities of reaching the states that have positive state utilities, and so will have lower expected cycle utilities and cumulative utilities. The benefit of vaccination equals the difference in cumulative utility between a representative member of the unvaccinated cohort and of the vaccinated cohort. The rate of return (RoR) to vaccination equals the vaccine benefit divided by the cost of vaccinating a representative cohort member, minus one.

We end with some notes on terminology.

In addition to the program population and elderly and comorbid subpopulation, we shall also refer to the “general population” and the “non-comorbid subpopulation.” The “general population” is simply the broader population of which the program population, elderly subpopulation, and comorbid subpopulation are subsets. More specifically, the elderly subpopulation simply consists of that subset of the general population that is aged 65 years and older in the base year. The general population aged 18-64 years in the base year, in turn, consists of the comorbid subpopulation, (which by our definition is limited to those aged 18-64 years in the base year), and the non-comorbid adults aged 18-64 years in the base year. Thus, the general population combines elderly and non-elderly adults, as well as comorbid and non-comorbid adults.

We perform scenario and sensitivity analysis with respect to our assumptions regarding vaccine efficacy and herd effects from pediatric PCV13 vaccination (PCV13 Pediatric). We shall refer to our central assumptions regarding these as the “base case” vaccine efficacy and herd effects scenarios. We shall call our counterfactual vaccine efficacy scenario the “age invariant baseline vaccine efficacy scenario.” In this context, baseline vaccine efficacy is vaccine efficacy immediately after vaccination, before any waning takes place.

# A3. Transition probabilities

Parametrizing the Markov model involves parameterizing the transition probabilities across Markov states and the utilities associated with those states. This section describes how we do the former. Moving from left to right in Figure 1, we see that this involves parametrizing the risks of death from non-PD-related causes, the risks of infection from the various manifestations of PD, and conditional on infection, the risks of PD-related death and various forms of PD-related disability.

Our program cohort is differentiated by age and by comorbidity status (whether a cohort member has any comorbidity or not), so our transition probabilities will themselves be age-and-comorbidity-status specific. To do this, we first compute age-specific transition probabilities for the general population, and then model age-specific transition probabilities for the comorbid subpopulation by scalar multiplication of those of the general population.

We begin by discussing the prevalence of comorbidity and non-comorbidity, and of various risk groups, within the general population in subsection A3.1. In subsection A3.2, we then discuss incidence rates, herd effects from PCV13 Pediatric, and vaccine efficacy, which together determine the transition probabilities flowing from node B in Figure 1. In subsection A3.3, we discuss case fatality rates and probabilities of disability, which together determine the transition probabilities flowing from nodes C, D, and E. In subsection A3.4, we discuss the probabilities of non-PD-related death, which determine the probabilities flowing from node A.

## **A3.1. Prevalence of comorbidity, non-comorbidity, and various risk groups within the general population**

Our comorbid subpopulation includes individuals aged 18-64 years during the base year with one or more of the following six distinct policy-relevant comorbidities: chronic obstructive pulmonary disease (COPD), asthma, diabetes, chronic kidney failure (CKF), chronic heart disease (CHD), and cancer. In this subsection, we compute the prevalence of each comorbidity, the prevalence of non-comorbid adults in the general population, and the proportions of the general population having low, medium, or high PD-related risks. We use these prevalence rates to construct scale factors with which to scale parameters that apply to Turkey’s general population in order to account for the impact on these parameters of the higher risk of infection stemming from one or more of the relevant comorbidities (see Section A3.2.3 and Section A4.4.7).

### **A3.1.1. Prevalence of comorbidities**

We obtain prevalence rates for COPD from Kocabaş (2010), ^^[[2]](#footnote-2)^^ asthma from Türk Turaks Derneği (2009),^[[3]](#footnote-3)^ diabetes from Satman et al. (2013),^[[4]](#footnote-4)^ CHD from Değertekin et al. (2012),^[[5]](#footnote-5)^ and cancer from the World Health Organization International Agency for Research on Cancer (2018).^[[6]](#footnote-6)^

We derive prevalence rates for CKF based on data from Ateş. Türkiye’de diyabet ve kronik böbrek hastalığı: CREDIT çalışması. Presentation by Türk Nefrologı Derneğı.^[[7]](#footnote-7)^ Slide 18 provides the prevalence of chronic kidney disease by age groups. We scale chronic kidney *disease* prevalence downward to produce an estimate of the prevalence of chronic kidney *failure* using Slide 21, which provides chronic kidney disease prevalence by stage, wherein chronic kidney failure consists of the last three stages. Our scale factor is therefore the ratio of the sum of the prevalence rates of the last three stages to the sum of the prevalence rates of all five stages, which gives us 32.5%. Thus, given the prevalence of chronic kidney disease $p_{CKD}^{x}$ from slide 18, for each discrete age $x$we derive the prevalence of chronic kidney failure as shown in Equation A3.1:

$$\begin{aligned} p_{CKF}^{x}=p_{CKD}^{x}*0.325\#\left( A3.1 \right) \end{aligned}$$

The six policy-relevant comorbidities that we analyze are listed in Table S1, along with each comorbidity’s age-specific prevalence rate.

**Table S1. Prevalence rates of comorbidities by condition and age**

| Age | COPD | Asthma | Diabetes | CKF | CHD | Cancer |
| --- | --- | --- | --- | --- | --- | --- |
| 18–29 | 0.19 | 0.074 | 0.165 | 0.032 | 0.020 | 0.0057 |
| 30–39 | 0.19 | 0.074 | 0.165 | 0.028 | 0.020 | 0.0057 |
| 40–49 | 0.19 | 0.074 | 0.165 | 0.041 | 0.020 | 0.0057 |
| 50–59 | 0.19 | 0.074 | 0.165 | 0.059 | 0.020 | 0.0057 |
| 60-64 | 0.19 | 0.074 | 0.165 | 0.106 | 0.020 | 0.0057 |
| 65-69 | 0.19 | 0.074 | 0.165 | 0.106 | 0.040 | 0.0057 |
| 70-74 | 0.19 | 0.074 | 0.165 | 0.134 | 0.040 | 0.0057 |
| 75-79 | 0.19 | 0.074 | 0.165 | 0.134 | 0.250 | 0.0057 |
| 80-84 | 0.19 | 0.074 | 0.165 | 0.178 | 0.250 | 0.0057 |
| 85 | 0.19 | 0.074 | 0.165 | 0.178 | 0.250 | 0.0057 |

Abbreviations: CHD = chronic heart disease; CKF = chronic kidney failure; COPD = chronic obstructive pulmonary disease

Our derivations of the prevalence of our policy-relevant comorbidities are based on the following assumptions:

1. These comorbidities are the only ones that are relevant, and therefore the only ones we model.
2. These comorbidities are not mutually exclusive, meaning an individual in the general population could have any, all, or none of them.
3. The probability of an individual in the general population having any of these comorbidities is equal to the prevalence rate of that comorbidity.
4. These probabilities are independent, meaning that having (or not having) one does not affect the likelihood of having any other.

Note that in (iv) above, we make the assumption of independence on the grounds of simplicity and conservatism. With respect to simplicity, our analysis focuses on six specific conditions, thus the total number of possible combinations of comorbidities is very large. Without the assumption of independence, the potential number of prevalence rates required to calibrate the model would be massive. The evidence base is insufficient to generate all the possible prevalence rates. Alternatively, we could allow for some combinations of comorbidities to be non-independent by investigating the related literature; however, this research would be challenging in a data-poor setting such as Turkey.

Moreover, the assumption of independence is conservative. If in reality one condition is more likely to render an individual vulnerable to other conditions, then our assumption of independence underestimates the proportion of the population with multiple comorbidities, and therefore the proportion of the population with any comorbidities. This yields conservative estimates of the benefit and RoR per vaccinated individual. This is because: (i) these benefits and RoRs are population-weighted averages across non-comorbid and comorbid adults; (ii) comorbid adults enjoy larger vaccination benefits than non-comorbid adults; and (iii) our independence assumption down-weights comorbid adults’ larger benefits.

For illustrative purposes, under the assumption of the independence of these comorbidities, Equation A3.2 gives the probability of an individual of age $x$ in the general population having COPD, asthma, and diabetes given prevalence rates $p$.

$$\begin{aligned} p_{COPD, asthma,diabetes}^{x}=p_{COPD}^{x}*p_{asthma}^{x}*p_{diabetes}^{x}\#\left( A3.2 \right) \end{aligned}$$

### **A3.1.2. Prevalence of non-comorbid adults**

An individual could also be free of all the six comorbidities, which we refer to hereafter as “non-comorbid.” Under the assumption of the independence of these comorbidities, Equation A3.3 gives the probability of being non-comorbid as the product across the comorbidities of the probabilities of not having each comorbidity.

$$\begin{aligned} p_{h}^{x}={(1-p}_{COPD}^{x})({1-p}_{asthma}^{x}){(1-p}_{diabetes}^{x}){(1-p}_{CKF}^{x})({1-p}_{CHD}^{x}){(1-p}_{cancer}^{x})\#\left( A3.3 \right) \end{aligned}$$

Table S2 gives the results of this calculation using the prevalence rates from Table S1.

**Table S2. Calculated**

**prevalence of non-comorbid**

**individuals**

| Age | $\boldsymbol{p}_{\boldsymbol{h}}^{\boldsymbol{x}}$ |
| --- | --- |
| 18–29 | 0.59 |
| 30–39 | 0.59 |
| 40–49 | 0.58 |
| 50–59 | 0.57 |
| 60–64 | 0.54 |
| 65-69 | 0.53 |
| 70-74 | 0.52 |
| 75-79 | 0.40 |
| 80-84 | 0.38 |
| 85 | 0.38 |

### **A3.1.3. Prevalence of low, medium, and high PD-related risks**

We sort our general population into three groups depending on whether they have low, medium, or high PD-related risks, where such risks involve increased incidence, lower vaccine efficacy, and higher case fatality rates. For this purpose, we rely on a taxonomy of comorbidity risks from Mangen et al. (2015), who list conditions belonging to medium- and high-risk groups.^^[[8]](#footnote-8)^^ They consider any condition not in their lists to be a low-risk condition. We follow their classification with the exception of asthma. While Mangen et al*.* (2015) do not list asthma, implying that it is a low-risk condition, we find evidence linking asthma to increased PD risk.^^[[9]](#footnote-9)^,^^[[10]](#footnote-10),^^[[11]](#footnote-11)^ We therefore classify asthma as a medium-risk condition. Table S3 presents the full list of conditions that we consider and their associated risk group.

**Table S3. Risk group by comorbidity**

| Comorbidity | Risk group |
| --- | --- |
| COPD | Medium |
| Asthma | Medium |
| Diabetes | Medium |
| CKF | High |
| CHD | Medium |
| Cancer | High |

Data source: Mangen et al. (2015) for CHD,

CKF, COPD, cancer, and diabetes.

Abbreviations: CHD = chronic heart disease;

CKF = chronic kidney failure;

COPD = chronic obstructive pulmonary disease

We classify all non-comorbid adults as low risk.

### **A3.1.4. Risk group prevalence rates**

We assume that an individual in the general population could have any, all, or none of the relevant comorbidities, and that the probabilities of having each comorbidity are independent (see Section A3.1.1). Based on these assumptions, we derive the probability that an individual in the general population belongs to a certain risk group.

We begin with the high-risk category. To derive the probability that an individual has at least one high-risk condition, we start with the probability that the individual has *no* high-risk condition. We equate the probability that an individual has a certain condition to the prevalence of that condition. Equation A3.4.a gives the age-specific probability of having a no high-risk condition:

$$\begin{aligned} p_{no high}^{x}={(1-p}_{CLF}^{x})({1-p}_{cancer}^{x})\#\left( A3.4.a \right) \end{aligned}$$

The probability of having at least one high-risk disease is the probability of *not* having a *no* high-risk condition, or 1 minus the probability given by Equation A3.4.a. This definition is illustrated in Equation A3.4.b:

$$\begin{aligned} p_{high}^{x}={1-p}_{no high}^{x} \\ =1-{(1-p}_{CLF}^{x})({1-p}_{cancer}^{x})\#\left( A3.4.b \right) \end{aligned}$$

We now move to the medium-risk category. As with the high-risk category, we start with the probability that the individual has *no* medium-risk condition. Equation A3.5.a gives this age-specific probability:

$$\begin{aligned} p_{no medium}^{x}={(1-p}_{COPD}^{x})({1-p}_{asthma}^{x}){(1-p}_{diabetes}^{x})\left( {1-p}_{CHD}^{x} \right)\#\left( A3.5.a \right) \end{aligned}$$

The probability of having at least one medium-risk disease is the probability of *not* having a *no* medium-risk condition, or 1 minus the probability given by Equation A3.5.a. This definition is illustrated in Equation A3.5.b:

$$\begin{aligned} p_{medium}^{x}={1-p}_{no medium}^{x} \\ =1-[{(1-p}_{COPD}^{x})({1-p}_{asthma}^{x}){(1-p}_{diabetes}^{x})\left( {1-p}_{CHD}^{x} \right)]\#\left( A3.5.b \right) \end{aligned}$$

For individuals with more than one comorbidity, we assign them their most severe comorbidity in terms of risk status. We do this by removing individuals with high-risk conditions from the medium-risk group defined by Equation A3.5.b. The probability of having a medium-risk condition *and no* high-risk condition is given by Equation A3.5.c:

$$\begin{aligned} p_{medium, no high}^{x}={(1-p}_{no medium}^{x})(p_{no high}^{x}) \\ =[1-{(1-p}_{COPD}^{x})({1-p}_{asthma}^{x}){(1-p}_{diabetes}^{x})\left( {1-p}_{CHD}^{x} \right)]* \\ \{\left( {1-p}_{CLF}^{x} \right)\left( {1-p}_{cancer}^{x} \right)\}\#\left( A3.5.c \right) \end{aligned}$$

The prevalence of any comorbidity is the sum of $p_{medium, no high}^{x}$ and $p_{high}^{x}$, as shown in Equation A3.6:

$$\begin{aligned} p_{c}^{x}=p_{medium, no high}^{x}+p_{high}^{x}\#\left( A3.6 \right) \end{aligned}$$

The prevalence rates calculated using Equation A3.6 are reported in Table 1 of the article.

We define the low-risk group as any individual without these comorbidities, which implies that $p_{low}^{x}$ equals 1 minus $p_{comorbidity}^{x}$ as shown in Equation A3.7:

$$\begin{aligned} p_{low}^{x}=1-p_{comorbidity}^{x}\#\left( A3.7 \right) \end{aligned}$$

We now have prevalence rates for the low-, medium- (and not high), and high-risk groups in the general population as presented in Table S4:

**Table S4. Calculated prevalence of risk groups by age, general**

**population^^[[12]](#footnote-12)^^**

| Age | $\boldsymbol{p}_{\boldsymbol{low}}^{\boldsymbol{x}}$ | $\boldsymbol{p}_{\boldsymbol{medium,no high}}^{\boldsymbol{x}}$ | $\boldsymbol{p}_{\boldsymbol{high}}^{\boldsymbol{x}}$ |
| --- | --- | --- | --- |
| 18–29 | 0.590 | 0.372 | 0.038 |
| 30–39 | 0.593 | 0.374 | 0.033 |
| 40–49 | 0.585 | 0.369 | 0.046 |
| 50–59 | 0.573 | 0.362 | 0.065 |
| 60–64 | 0.545 | 0.344 | 0.111 |
| 65-69 | 0.534 | 0.355 | 0.111 |
| 70-74 | 0.517 | 0.344 | 0.139 |
| 75-79 | 0.404 | 0.457 | 0.139 |
| 80-84 | 0.384 | 0.434 | 0.182 |
| 85 | 0.384 | 0.434 | 0.182 |

Since our comorbid group consists of those who are medium- and high-risk, we derive the prevalence of each risk group in the comorbid subpopulation by scaling up $p_{medium,no high}^{x}$ and $p_{high}^{x}$ in Table S4 such that they sum to 1. Denoting the prevalence in the comorbid subpopulation for individuals age $x$ in the medium-risk group by ${pc}_{medium,no high}^{x}$, and that for individuals age $x$ in the high-risk group by ${pc}_{high}^{x}$, these prevalence rates are expressed in Equations A3.8.a and A3.8.b:

$$\begin{aligned} {pc}_{medium,no high}^{x}=\frac{p_{medium,no high}^{x}}{p_{medium,no high}^{x}+p_{high}^{x}}\#\left( A3.8.a \right) \end{aligned}$$

$$\begin{aligned} {pc}_{high}^{x}=\frac{p_{high}^{x}}{p_{medium,no high}^{x}+p_{high}^{x}}\#\left( A3.8.b \right) \end{aligned}$$

Table S5 presents our calculations of ${pc}_{medium,no high}^{x}$ and ${pc}_{high}^{x}$. Note that ${pc}_{low}^{x}$ is always 0 because, by definition, low-risk individuals are not in the comorbid subpopulation.

**Table S5. Calculated prevalence of risk groups by age, comorbid**

**subpopulation**

| Age | $\boldsymbol{pc}_{\boldsymbol{low}}^{\boldsymbol{x}}$ | $\boldsymbol{pc}_{\boldsymbol{medium,no high}}^{\boldsymbol{x}}$ | $\boldsymbol{pc}_{\boldsymbol{high}}^{\boldsymbol{x}}$ |
| --- | --- | --- | --- |
| 18–29 | 0 | 0.91 | 0.09 |
| 30–39 | 0 | 0.92 | 0.08 |
| 40–49 | 0 | 0.89 | 0.11 |
| 50–59 | 0 | 0.85 | 0.15 |
| 60–64 | 0 | 0.76 | 0.24 |
| 65-69 | 0 | 0.76 | 0.24 |
| 70-74 | 0 | 0.71 | 0.29 |
| 75-79 | 0 | 0.77 | 0.23 |
| 80-84 | 0 | 0.70 | 0.30 |
| 85 | 0 | 0.70 | 0.30 |

## **A3.2. Incidence rates, herd effects, and vaccine efficacy**

As a cohort moves through the Markov model, the probabilities of contracting the various manifestations of PD at chance node B are given by incidence rates. These incidence rates vary with age and comorbidity status (subsection A3.2.3), the impact of herd effects from PCV13 Pediatric (subsection A3.2.2), and vaccination status (subsection A3.2.5). The impact of vaccination on incidence in turn depends on baseline vaccine efficacy (i.e., vaccine efficacy immediately after vaccination, before any waning takes effect), age and comorbidity status, and waning.

We assume that adult PCV13 vaccination (PCV13 Adult) does not reduce the severity of infection or cause serotype replacement or herd effects. This assumption limits the impact of PCV13 vaccination to reducing PCV13-serotype (vaccine-type) incidence, and limits our focus to vaccine-type (as opposed to all-type) incidence and vaccine efficacy.

### **A3.2.1. Vaccine-type incidence rates in the absence of vaccination**

This subsection describes how we construct age- and comorbidity-specific vaccine-type incidence rates in the absence of herd effects from PCV13 Pediatric. We treat inpatient community-acquired pneumonia (CAP), outpatient CAP, bacteremia, and meningitis as distinct manifestations (see Figure 1 of the article). Therefore, to parameterize our Markov model, we calculate base year vaccine-type incidence rates for each of these manifestations. In cases where the input parameters and data used in these calculations predate 2015, we assume that the values hold for 2015.

We build our vaccine-type CAP and invasive pneumococcal disease (IPD) incidence rates using Turkey-specific 2017 Global Burden of Disease (GBD) estimates of lower respiratory infection (LRI) incidence and pneumococcal meningitis incidence, respectively.^^[[13]](#footnote-13)^^

#### **A3.2.1.i. Community-acquired pneumonia vaccine-type incidence rates for the general population**

We calculate (all-type) pneumococcal pneumonia incidence rates by multiplying the Turkey-specific 2017 GBD LRI incidence rates by the ratio of global pneumococcal pneumonia incidence to global LRI incidence. This ratio, which is computed from Table 2 in GBD 2016 LRI Collaborators,^[[14]](#footnote-14)^ equals 26.7/(26.7+3.4+0.9+5.3)=0.736 across all ages, 0.665 in ages below 5, and 0.767 in ages 70 and up. (These global incidence rates are in turn generated by the GBD project on the basis of population attribution fractions that disaggregate LRI into the following four etiologies: streptococcus pneumonia, respiratory syncytial virus, haemophilus influenza type B, and influenza).^[[15]](#footnote-15)^ We take the “all ages” value of 0.736 to apply to ages 5 to 69.

We assume that 19.4% of the resulting pneumococcal pneumonia consists of inpatient cases, and the remainder outpatient cases, based on the CAP hospitalization for at risk adults and elderly in Turkey from Akin et al*.* (2011).^^[[16]](#footnote-16)^^ This rate is based on 2004 Turkey Ministry of Health data. We assume that 68% of inpatient and outpatient cases are PCV13-type on the basis of a Turkish estimate of PCV13 vaccine-type coverage in invasive and non-invasive pneumococcal disease from Oksuz and Gurler (2017).^^[[17]](#footnote-17)^^ In sensitivity analysis, we use alternative PCV13-type coverage rates of 85.5% and 56.6% based on Ceyhan et al*.* (2016)’s^[[18]](#footnote-18)^ estimates of PCV13 coverage of invasive isolates for Turkey and Hungary, respectively.

Table S11 presents these base year vaccine-type pneumococcal pneumonia incidence rates along with the IPD incidence rates whose calculations we discuss next.

#### **A3.2.1.ii. Invasive pneumococcal disease vaccine-type incidence rates for the general population**

Our meningitis incidence rates are the 2017 Turkey-specific GBD incidence rates for pneumococcal meningitis.^^[[19]](#footnote-19)^^ We calculate bacteremia incidence rates by multiplying the GBD meningitis incidence rates by the ratio of bacteremia cases to meningitis cases that we obtain from 2016 data for six comparator countries from the European Centre for Disease Prevention and Control (ECDC) *Surveillance Atlas of Infectious Diseases* (Turkey does not have the relevant data in this database).^[[20]](#footnote-20)^ These countries are the Czech Republic, Greece, Hungary, Poland, Slovakia, and Slovenia. We selected these countries based on the following criteria: (1) they have 2016 ECDC data on both the number of IPD cases and number of IPD deaths; (2) they are in close geographic proximity to Turkey (i.e., in or adjacent to Central Europe or Eastern Europe); (3) the per capita GDP is close to, or slightly higher than, Turkey’s; and (4) the Socio-Demographic Index (SDI)^[[21]](#footnote-21)^ is equal to, or slightly higher than, Turkey’s. Table S6 provides the country-specific values for these criteria for each comparator country:

| **Table S6. European Center for Disease Control comparator countries** | | |
| --- | --- | --- |
| **Country** | **GDP per capita (2016 international dollars)^1^** | **Socio-Demographic Index^2^** |
| Czech Republic | $34,749 | High |
| Greece | $26,765 | High Middle |
| Hungary | $26,701 | High |
| Poland | $27,383 | High |
| Slovakia | $30,460 | High |
| Slovenia | $32,723 | High |
| Turkey | $25,247 | High Middle |
| 1. Source: World Development Indicators, The World Bank. <https://databank.worldbank.org/reports.aspx?source=world-development-indicators>. | | |
| 2. Source: Global Burden of Disease Study 2015. Global Burden of Disease Study 2015 (GBD 2015) Socio-Demographic Index (SDI) 1980–2015. Seattle, United States: Institute for Health Metrics and Evaluation (IHME), 2016. | | |

The ECDC provides country-specific counts of the number of IPD cases and deaths for the following age groups: all ages, 0-<1 year, 1-4 years, and 65 years and older. For these age groups, the ECDC also provides country-specific distributions of cases and deaths amongst the following IPD manifestations (manifestations are referred to as “clinical presentations” in the ECDC Surveillance Atlas): bacteraemic pneumonia, septicaemia, meningitis, meningitis/septicaemia, other, and unknown.

The ECDC counts and distributions described above are provided in Table S7 (we use the data on deaths for the computation of CFRs in Section A3.3.2 below). For example, for ages 65 and older in the Czech Republic, there are 37 confirmed deaths of which 21.6% are caused by septicaemia, 56.8% by bacteraemic pneumonia, 13.5% by meningitis, 0% by other manifestations, 0% by meningitis/septicaemia, and 8.1% by manifestations that are unknown. For this age group in the Czech Republic, there are 157 confirmed cases of which 24.8% are caused by septicaemia, 61.8% by bacteraemic pneumonia, 10.8% by meningitis, 0% by other manifestations, 0% by meningitis/septicaemia, and 2.5% by manifestations that are unknown.

Using the counts and percentages in Table S7, we can calculate the number of cases and deaths by country and manifestation for all ages, 0-<1 year, 1-4 years, and 65 years and older. These calculations are provided in Table S8. For example, for ages 65 and older in the Czech Republic, the number deaths that are caused by septicaemia is equal to 21.6 percent of 37, or 7.99. For this age group in the Czech Republic, the number of cases due to septicaemia is equal to 24.8 percent of 157, or 38.94.

| **Table S7. Number of deaths and cases and distribution of deaths and cases amongst IPD outcome** | | | | | | | | | | | | | | | | | | | | | | | |
| --- | --- | --- | --- | --- | --- | --- | --- | --- | --- | --- | --- | --- | --- | --- | --- | --- | --- | --- | --- | --- | --- | --- | --- |
|  | **Czech Republic** | | | | | **Greece** | | | | **Hungary** | | | | **Poland** | | | | **Slovakia** | | | | **Slovenia** | |
|  | **Confirmed number of deaths and cases: All ages** | | | | | | | | | | | | | | | | | | | | | | |
|  | **deaths** | | **cases** | | | **deaths** | **cases** | | | **deaths** | | **cases** | | **deaths** | | **cases** | | **deaths** | | **Cases** | | **deaths** | **cases** |
|  | 66 | | 323 | | | 2 | 52 | | | 67 | | 226 | | 246 | | 962 | | 3 | | 59 | | 12 | 281 |
| **IPD outcome** | **Distribution of deaths and cases amongst IPD outcomes: All ages** | | | | | | | | | | | | | | | | | | | | | | |
| Septicemia | 26% | | 23% | | | 0% | 0% | | | 15% | | 15% | | 49% | | 43% | | 67% | | 42% | | 0% | 14% |
| Bacteraemic pneumonia | 55% | | 59% | | | 0% | 0% | | | 70% | | 66% | | 29% | | 28% | | 0% | | 20% | | 42% | 23% |
| Meningitis | 14% | | 15% | | | 50% | 71% | | | 15% | | 19% | | 8% | | 12% | | 33% | | 27% | | 0% | 4% |
| Other | 0% | | 0% | | | 0% | 0% | | | 0% | | 0% | | 12% | | 10% | | 0% | | 10% | | 8% | 6% |
| Meningitis/septicaemia | 0% | | 0% | | | 50% | 29% | | | 0% | | 0% | | 3% | | 6% | | 0% | | 0% | | 0% | 0% |
| Unknown | 6% | | 3% | | | 0% | 0% | | | 0% | | 0% | | 0% | | 0% | | 0% | | 0% | | 50% | 54% |
|  | **Confirmed number of deaths and cases: Age <1** | | | | | | | | | | | | | | | | | | | | | | |
|  | **deaths** | | **cases** | | | **deaths** | **cases** | | | **deaths** | | **cases** | | **deaths** | | **cases** | | **deaths** | | **Cases** | | **deaths** | **cases** |
|  | 0 | | 1 | | | 0 | 1 | | | 1 | | 5 | | 1 | | 26 | | 0 | | 0 | | 0 | 5 |
| **IPD outcome** | **Distribution of deaths and cases amongst IPD outcomes: Age <1** | | | | | | | | | | | | | | | | | | | | | | |
| Septicemia | 0.00% | | 100.00% | | | 0.00% | 0.00% | | | 0.00% | | 20.00% | | 100.00% | | 46.20% | | 0.00% | | 0.00% | | 0.00% | 0.00% |
| Bacteraemic pneumonia | 0.00% | | 0.00% | | | 0.00% | 0.00% | | | 0.00% | | 20.00% | | 0.00% | | 3.80% | | 0.00% | | 0.00% | | 0.00% | 0.00% |
| Meningitis | 0.00% | | 0.00% | | | 0.00% | 100.00% | | | 100.00% | | 60.00% | | 0.00% | | 19.20% | | 0.00% | | 0.00% | | 0.00% | 0.00% |
| Other | 0.00% | | 0.00% | | | 0.00% | 0.00% | | | 0.00% | | 0.00% | | 0.00% | | 7.70% | | 0.00% | | 0.00% | | 0.00% | 40.00% |
| Meningitis/septicaemia | 0.00% | | 0.00% | | | 0.00% | 0.00% | | | 0.00% | | 0.00% | | 0.00% | | 23.10% | | 0.00% | | 0.00% | | 0.00% | 0.00% |
| Unknown | 0.00% | | 0.00% | | | 0.00% | 0.00% | | | 0.00% | | 0.00% | | 0.00% | | 0.00% | | 0.00% | | 0.00% | | 0.00% | 60.00% |
|  | **Confirmed number of deaths and cases: Ages 1-4 years** | | | | | | | | | | | | | | | | | | | | | | |
|  | **deaths** | | **cases** | | | **deaths** | **cases** | | | **deaths** | | **cases** | | **deaths** | | **cases** | | **deaths** | | **Cases** | | **deaths** | **cases** |
|  | 0 | | 12 | | | 0 | 4 | | | 2 | | 10 | | 0 | | 65 | | 0 | | 8 | | 0 | 32 |
| **IPD outcome** | **Distribution of deaths and cases amongst IPD outcomes: Ages 1-4** | | | | | | | | | | | | | | | | | | | | | | |
| Septicemia | 0.00% | | 25.00% | | | 0.00% | 0.00% | | | 0.00% | | 20.00% | | 0.00% | | 49.20% | | 0.00% | | 37.50% | | 0.00% | 18.80% |
| Bacteraemic pneumonia | 0.00% | | 66.70% | | | 0.00% | 0.00% | | | 50.00% | | 70.00% | | 0.00% | | 21.50% | | 0.00% | | 62.50% | | 0.00% | 21.90% |
| Meningitis | 0.00% | | 8.30% | | | 0.00% | 75.00% | | | 50.00% | | 10.00% | | 0.00% | | 10.80% | | 0.00% | | 0.00% | | 0.00% | 0.00% |
| Other | 0.00% | | 0.00% | | | 0.00% | 0.00% | | | 0.00% | | 0.00% | | 0.00% | | 10.80% | | 0.00% | | 0.00% | | 0.00% | 6.30% |
| Meningitis/septicaemia | 0.00% | | 0.00% | | | 0.00% | 25.00% | | | 0.00% | | 0.00% | | 0.00% | | 7.70% | | 0.00% | | 0.00% | | 0.00% | 0.00% |
| Unknown | 0.00% | | 0.00% | | | 0.00% | 0.00% | | | 0.00% | | 0.00% | | 0.00% | | 0.00% | | 0.00% | | 0.00% | | 0.00% | 53.10% |
|  | | | | | | | | | | | | | | | | | | | | | | | |
| **Table S7. Number of deaths and cases and distribution of deaths and cases amongst IPD outcome. Continued** | | | | | | | | | | | | | | | | | | | | | | | |
|  | | **Czech Republic** | | | **Greece** | | | | **Hungary** | | | | **Poland** | | | | **Slovakia** | | | | **Slovenia** | | |
|  | | **Confirmed number of deaths and cases: Ages 65 and older** | | | | | | | | | | | | | | | | | | | | | |
|  | | **deaths** | | **cases** | **deaths** | | | **cases** | **deaths** | | **cases** | | **deaths** | | **cases** | | **deaths** | | **cases** | | **deaths** | | **cases** |
|  | | 37 | | 157 | 2 | | | 14 | 36 | | 104 | | 138 | | 385 | | 1 | | 15 | | 9 | | 144 |
| **IPD outcome** | | **Distribution of deaths and cases amongst IPD outcomes: Ages 65 and older** | | | | | | | | | | | | | | | | | | | | | |
| Septicemia | | 21.60% | | 24.80% | 0.00% | | | 0.00% | 11.10% | | 15.40% | | 47.80% | | 44.70% | | 100.00% | | 46.70% | | 0.00% | | 14.60% |
| Bacteraemic pneumonia | | 56.80% | | 61.80% | 0.00% | | | 0.00% | 72.20% | | 71.20% | | 33.30% | | 36.40% | | 0.00% | | 26.70% | | 33.30% | | 21.50% |
| Meningitis | | 13.50% | | 10.80% | 50.00% | | | 78.60% | 16.70% | | 13.50% | | 7.20% | | 6.50% | | 0.00% | | 20.00% | | 0.00% | | 3.50% |
| Other | | 0.00% | | 0.00% | 0.00% | | | 0.00% | 0.00% | | 0.00% | | 10.10% | | 8.80% | | 0.00% | | 6.70% | | 11.10% | | 4.20% |
| Meningitis/septicaemia | | 0.00% | | 0.00% | 50.00% | | | 21.40% | 0.00% | | 0.00% | | 1.40% | | 3.60% | | 0.00% | | 0.00% | | 0.00% | | 0.00% |
| Unknown | | 8.10% | | 2.50% | 0.00% | | | 0.00% | 0.00% | | 0.00% | | 0.00% | | 0.00% | | 0.00% | | 0.00% | | 55.60% | | 56.30% |
| Data Source: 2016 ECDC Surveillance Atlas of Infectious Diseases (https://atlas.ecdc.europa.eu/public/index.aspx). | | | | | | | | | | | | | | | | | | | | | | | |

| **Table S8. Calculated number of deaths and cases by IPD outcome** | | | | | | | | | | | | | |
| --- | --- | --- | --- | --- | --- | --- | --- | --- | --- | --- | --- | --- | --- |
|  |  | **Czech Republic** | | **Greece** | | **Hungary** | | **Poland** | | **Slovakia** | | **Slovenia** | |
| **Age** | **IPD outcome** | **deaths** | **cases** | **deaths** | **cases** | **deaths** | **cases** | **deaths** | **cases** | **deaths** | **cases** | **deaths** | **cases** |
| All ages | Septicemia | 17.03 | 74.94 | 0.00 | 0.00 | 9.98 | 33.90 | 120.05 | 416.55 | 2.00 | 25.02 | 0.00 | 37.94 |
| All ages | Bacteraemic pneumonia | 35.97 | 189.92 | 0.00 | 0.00 | 46.97 | 148.93 | 71.09 | 272.25 | 0.00 | 11.98 | 5.00 | 64.07 |
| All ages | Meningitis | 8.98 | 49.10 | 1.00 | 37.02 | 9.98 | 42.94 | 18.94 | 116.40 | 1.00 | 15.99 | 0.00 | 10.96 |
| All ages | Other | 0.00 | 0.00 | 0.00 | 0.00 | 0.00 | 0.00 | 29.03 | 97.16 | 0.00 | 6.02 | 1.00 | 16.86 |
| All ages | Meningitis/septicaemia | 0.00 | 0.00 | 1.00 | 14.98 | 0.00 | 0.00 | 6.89 | 59.64 | 0.00 | 0.00 | 0.00 | 0.00 |
| All ages | Unknown | 4.03 | 9.04 | 0.00 | 0.00 | 0.00 | 0.00 | 0.00 | 0.00 | 0.00 | 0.00 | 6.00 | 150.90 |
|  |  |  |  |  |  |  |  |  |  |  |  |  |  |
| Age<1 | Septicemia | 0.00 | 1.00 | 0.00 | 0.00 | 0.00 | 1.00 | 1.00 | 12.01 | 0.00 | 0.00 | 0.00 | 0.00 |
| Age<1 | Bacteraemic pneumonia | 0.00 | 0.00 | 0.00 | 0.00 | 0.00 | 1.00 | 0.00 | 0.99 | 0.00 | 0.00 | 0.00 | 0.00 |
| Age<1 | Meningitis | 0.00 | 0.00 | 0.00 | 1.00 | 1.00 | 3.00 | 0.00 | 4.99 | 0.00 | 0.00 | 0.00 | 0.00 |
| Age<1 | Other | 0.00 | 0.00 | 0.00 | 0.00 | 0.00 | 0.00 | 0.00 | 2.00 | 0.00 | 0.00 | 0.00 | 2.00 |
| Age<1 | Meningitis/septicaemia | 0.00 | 0.00 | 0.00 | 1.00 | 0.00 | 0.00 | 0.00 | 6.01 | 0.00 | 0.00 | 0.00 | 0.00 |
| Age<1 | Unknown | 0.00 | 0.00 | 0.00 | 0.00 | 0.00 | 0.00 | 0.00 | 0.00 | 0.00 | 0.00 | 0.00 | 3.00 |
|  |  |  |  |  |  |  |  |  |  |  |  |  |  |
| Ages 1-4 | Septicemia | 0.00 | 3.00 | 0.00 | 0.00 | 0.00 | 2.00 | 0.00 | 31.98 | 0.00 | 3.00 | 0.00 | 6.02 |
| Ages 1-4 | Bacteraemic pneumonia | 0.00 | 8.00 | 0.00 | 0.00 | 1.00 | 7.00 | 0.00 | 13.98 | 0.00 | 5.00 | 0.00 | 7.01 |
| Ages 1-4 | Meningitis | 0.00 | 1.00 | 0.00 | 3.00 | 1.00 | 1.00 | 0.00 | 7.02 | 0.00 | 0.00 | 0.00 | 0.00 |
| Ages 1-4 | Other | 0.00 | 0.00 | 0.00 | 0.00 | 0.00 | 0.00 | 0.00 | 7.02 | 0.00 | 0.00 | 0.00 | 2.02 |
| Ages 1-4 | Meningitis/septicaemia | 0.00 | 0.00 | 0.00 | 1.00 | 0.00 | 0.00 | 0.00 | 5.01 | 0.00 | 0.00 | 0.00 | 0.00 |
| Ages 1-4 | Unknown | 0.00 | 0.00 | 0.00 | 0.00 | 0.00 | 0.00 | 0.00 | 0.00 | 0.00 | 0.00 | 0.00 | 16.99 |
|  |  |  |  |  |  |  |  |  |  |  |  |  |  |
| Ages 65+ | Septicemia | 7.99 | 38.94 | 0.00 | 0.00 | 4.00 | 16.02 | 65.96 | 172.10 | 1.00 | 7.01 | 0.00 | 21.02 |
| Ages 65+ | Bacteraemic pneumonia | 21.02 | 97.03 | 0.00 | 0.00 | 25.99 | 74.05 | 45.95 | 140.14 | 0.00 | 4.01 | 3.00 | 30.96 |
| Ages 65+ | Meningitis | 5.00 | 16.96 | 1.00 | 11.00 | 6.01 | 14.04 | 9.94 | 25.03 | 0.00 | 3.00 | 0.00 | 5.04 |
| Ages 65+ | Other | 0.00 | 0.00 | 0.00 | 0.00 | 0.00 | 0.00 | 13.94 | 33.88 | 0.00 | 1.01 | 1.00 | 6.05 |
| Ages 65+ | Meningitis/septicaemia | 0.00 | 0.00 | 1.00 | 3.00 | 0.00 | 0.00 | 1.93 | 13.86 | 0.00 | 0.00 | 0.00 | 0.00 |
| Ages 65+ | Unknown | 3.00 | 3.93 | 0.00 | 0.00 | 0.00 | 0.00 | 0.00 | 0.00 | 0.00 | 0.00 | 5.00 | 81.07 |

Data Source: 2016 ECDC Surveillance Atlas of Infectious Diseases (https://atlas.ecdc.europa.eu/public/index.aspx).

We calculate the counts of deaths and cases for ages 5-64 as equal to the counts for all ages minus the sum of the counts for age 0-<1 year, 1-4 years, and 65 years and older. These calculations are provided in Table S9. For example, for ages 5-64 in the Czech Republic, the number deaths that are caused by septicaemia is equal to 17.03 - (0 + 0 + 7.99) = 9.04. For this age group in the Czech Republic, the number of cases that are due to septicaemia is equal to 74.97 – (1 + 3 + 38.94) = 32.0.

| **Table S9. Calculated number of deaths and cases by IPD manifestation for Ages 5-64** | | | | | | | | | | | | | | |
| --- | --- | --- | --- | --- | --- | --- | --- | --- | --- | --- | --- | --- | --- | --- |
|  | **Czech Republic** | | **Greece** | | **Hungary** | | **Poland** | | **Slovakia** | | **Slovenia** | | **All countries combined** | |
|  | **Number of deaths and cases: Ages 5-64** | | | | | | | | | | | | | |
| **IPD manifestation** | **deaths** | **cases** | **deaths** | **cases** | **deaths** | **cases** | **deaths** | **cases** | **deaths** | **Cases** | **deaths** | **cases** | **deaths** | **cases** |
| Septicemia | 9.04 | 32.00 | 0.00 | 0.00 | 5.99 | 14.88 | 53.08 | 200.46 | 1.00 | 15.01 | 0.00 | 10.90 | 69.11 | 273.25 |
| Bacteraemic pneumonia | 14.95 | 84.89 | 0.00 | 0.00 | 19.98 | 66.89 | 25.14 | 117.14 | 0.00 | 2.97 | 2.01 | 26.10 | 62.08 | 298.00 |
| Meningitis | 3.98 | 31.14 | 0.00 | 22.02 | 1.97 | 24.90 | 9.01 | 79.37 | 1.00 | 12.99 | 0.00 | 5.92 | 15.96 | 176.34 |
| Other | 0.00 | 0.00 | 0.00 | 0.00 | 0.00 | 0.00 | 15.09 | 54.26 | 0.00 | 5.01 | 0.00 | 6.80 | 15.09 | 66.07 |
| Meningitis/septicaemia | 0.00 | 0.00 | 0.00 | 9.98 | 0.00 | 0.00 | 4.96 | 34.77 | 0.00 | 0.00 | 0.00 | 0.00 | 4.96 | 44.75 |
| Unknown | 1.03 | 5.12 | 0.00 | 0.00 | 0.00 | 0.00 | 0.00 | 0.00 | 0.00 | 0.00 | 1.00 | 49.83 | 2.03 | 54.95 |
|  |  |  |  |  |  |  |  |  |  |  |  |  |  |  |
| **Septicemia+ Bacteraemic pneumonia+ Meningitis** | **27.97** | **148.04** | **0.00** | **22.02** | **27.93** | **106.67** | **87.23** | **396.97** | **2.00** | **30.97** | **2.01** | **42.91** | **147.14** | **747.58** |

Data Source: 2016 ECDC Surveillance Atlas of Infectious Diseases (https://atlas.ecdc.europa.eu/public/index.aspx).

Summing deaths and cases across all countries, we obtain Table S10. (Note that henceforth, we exclude from our tables the age<1 and ages 1-4 age groups as they are no longer needed in our analysis of the ECDC data.)

| **Table S10. Calculated number of deaths and cases by IPD manifestation** | | | |
| --- | --- | --- | --- |
| **All countries combined** | | | |
| **Age** | **IPD manifestation** | **deaths** | **cases** |
| Ages 5-64 | Septicemia | 69.11 | 273.25 |
| Ages 5-64 | Bacteraemic pneumonia | 62.08 | 298.00 |
| Ages 5-64 | Bacteremia = Septicemia + Bacteraemic pneumonia | 131.18 | 571.24 |
| Ages 5-64 | Meningitis | 15.96 | 176.34 |
| Ages 5-64 | IPD = Bacteremia + Meningitis | 147.14 | 747.58 |
| Ages 65+ | Septicemia | 78.95 | 255.08 |
| Ages 65+ | Bacteraemic pneumonia | 95.96 | 346.18 |
| Ages 65+ | Bacteremia = Septicemia + Bacteraemic pneumonia | 174.91 | 601.26 |
| Ages 65+ | Meningitis | 21.94 | 75.07 |
| Ages 65+ | IPD = Bacteremia + Meningitis | 196.85 | 676.32 |

Data Source: 2016 ECDC Surveillance Atlas of Infectious Diseases (https://atlas.ecdc.europa.eu/public/index.aspx).

As shown in Table S10, the ratio of bacteremia cases to meningitis cases are 571.24/176.34 = 3.24 for ages 6-64, and 601.26/75.07 = 8.01 for ages 65 and older. We obtain all-type bacteremia incidence rates by multiplying the GBD meningitis incidence rates discussed above by the age-specific ratio.

To complete our construction of bacteremia and meningitis incidence rates, for both manifestations we assume the same 68% vaccine-type coverage to convert our all-type incidence rates discussed above to vaccine-type. Table S11 presents these base year vaccine-type invasive pneumococcal disease incidence rates along with those for CAP.

| **Table S11. Calculated 2015 vaccine-type incidence per 100,000 population without herd effects from PCV13 Pediatric** | | | | |
| --- | --- | --- | --- | --- |
|  | **Pneumococcal pneumonia** | | **Invasive pneumococcal disease** | |
| **Age** | **Inpatient** | **Outpatient** | **Bacteremia** | **Meningitis** |
| 18-19 | 605.41 | 2,515.26 | 0.41 | 0.13 |
| 20-24 | 416.42 | 1,730.07 | 0.29 | 0.09 |
| 25-29 | 378.75 | 1,573.57 | 0.28 | 0.08 |
| 30-34 | 341.12 | 1,417.23 | 0.27 | 0.08 |
| 35-39 | 303.55 | 1,261.16 | 0.28 | 0.09 |
| 40-44 | 290.56 | 1,207.17 | 0.27 | 0.08 |
| 45-49 | 302.07 | 1,254.99 | 0.25 | 0.08 |
| 50-54 | 313.78 | 1,303.65 | 0.26 | 0.08 |
| 55-59 | 325.48 | 1,352.27 | 0.29 | 0.09 |
| 60-64 | 385.30 | 1,600.80 | 0.33 | 0.10 |
| 65-69 | 492.61 | 2,046.60 | 1.00 | 0.13 |
| 70-74 | 624.39 | 2,594.11 | 1.23 | 0.15 |
| 75-79 | 733.46 | 3,047.24 | 1.51 | 0.19 |
| 80-84 | 905.81 | 3,763.29 | 2.34 | 0.29 |
| 85 | 1,128.03 | 4,686.55 | 3.75 | 0.47 |
| Data Sources: 2016 ECDC Surveillance Atlas of Infectious Diseases (<https://atlas.ecdc.europa.eu/public/index.aspx>); Global Burden of Disease. IHME (2017). <http://ghdx.healthdata.org/gbd-2017> | | | | |

The GBD does not account for herd immunity in their estimates.^^[[22]](#footnote-22)^^ However, the empirical literature suggests that widespread childhood pneumococcal conjugate vaccination leads to herd effects that reduce adult PCV13-serotype incidence.^[[23]](#footnote-23)^ Therefore, in our base case analysis we adjust the incidence rates in Table S11 for herd effects from PCV13 Pediatric using the methodology described in Section A3.2.2.

### **A3.2.2. Herd effects from PCV13 Pediatric**

To adjust the GBD-based incidence rates in Table S11 for herd immunity from PCV13 Pediatric, we make three assumptions. The first is that herd effects eventually reduce the above vaccine-type incidence rates by 88% (or equivalently, that post-herd effects incidence is 12% of the above pre-herd-effects incidence rates),^^[[24]](#footnote-24)^^ which we base on the findings in Harboe et al*.* (2014) and Waight et al*.* (2015).^^[[25]](#footnote-25)^^ Harboe et al*.* (2014) find an incidence rate ratio (IRR) of 0.12 between the 7-valent pneumococcal conjugate vaccine (PCV7) serotype IPD incidence in the pre-PCV era in Denmark (2000–2007) and the PCV13 era (2011–2013), while Waight et al*.* (2015) find an IRR of 0.11 between PCV7-serotype IPD incidence in the pre-PCV13 era in England (2008–2010) and the PCV13 era (2013–2014). Thus, we assume that the PCV13-serotype IPD and CAP incidence rates will face the same IRR (i.e., 0.12) as the PCV7-serotype IPD incidence rate did.

The second assumption is that by the time of our base year of 2015, half of the duration of herd effects had already elapsed. This assumption is in turn based on the assumption that a steady state is reached in 2019, which is common in the literature.^[[26]](#footnote-26),^^[[27]](#footnote-27)^ Therefore, our 2015 incidence rates are equal to $\sqrt{0.12}$ of the pre-herd effects estimates in Table 11. These incidence rates are presented in Table 1 of the article.

Our third assumption is that there is constant proportional decline in incidence from 2015 to 2019. This implies that for every year from 2016 to 2019, incidence rates are ${0.12}^{1/8}$ of the year prior, and 2019 incidence rates are $\sqrt{0.12}$ of 2015 incidence rates and 0.12 of pre-herd-effect incidence rates. Thus, the incidence rate in 2016 is ${0.12}^{1/8}$ times the incidence rate in 2015, the incidence rate in 2017 is ${0.12}^{1/8}$ x ${0.12}^{1/8}$ = ${0.12}^{1/4}$ times the incidence rate in 2015, and so on such that the total reduction across both halves of the herd effect duration is 88%.

### **A3.2.3. Vaccine-type community-acquired pneumonia and invasive pneumococcal disease incidence rates for the comorbid subpopulation**

In this subsection, we describe our comorbidity adjustments to the CAP and IPD incidence rates for the general population in order to account for the impact on these parameters of the higher risk of infection stemming from one or more of the relevant comorbidities. Our basic approach is use data from another country to construct age- and manifestation-specific ratios of the incidence rates in comorbid individuals to that in the general population, and then multiply the incidence rates from the Turkish general population (presented in Table 1 of the article) by the appropriate ratio. To construct these ratios, we use Dutch data on risk-group-specific CAP and IPD incidence rates from Mangen et al. (2015).^^[[28]](#footnote-28)^^ The age-specific ratios of IPD, inpatient CAP, and outpatient CAP incidence in comorbid adults to those in the general population are expressed in Equation A3.9a, A3.9b, A3.9c, respectively:

$$\begin{aligned} {SF}_{IPD}^{x}=\frac{{IPD}_{low}^{x}*{pc}_{low}^{x}+{IPD}_{medium}^{x}*{pc}_{medium}^{x}+{IPD}_{high}^{x}*{pc}_{high}^{x}}{{IPD}_{low}^{x}*p_{low}^{x}+{IPD}_{medium}^{x}*p_{medium}^{x}+ {IPD}_{high}^{x}*p_{high}^{x}}\#(A3.9a) \end{aligned}$$

$$\begin{aligned} {SF}_{ICAP}^{x}=\frac{{ICAP}_{low}^{x}*{pc}_{low}^{x}+{ICAP}_{medium}^{x}*{pc}_{medium}^{x}+{ICAP}_{high}^{x}*{pc}_{high}^{x}}{{ICAP}_{low}^{x}*p_{low}^{x}+{ICAP}_{medium}^{x}*p_{medium}^{x}+ {ICAP}_{high}^{x}*p_{high}^{x}}\#(A3.9b) \end{aligned}$$

$$\begin{aligned} {SF}_{OCAP}^{x}=\frac{{OCAP}_{low}^{x}*{pc}_{low}^{x}+{OCAP}_{medium}^{x}*{pc}_{medium}^{x}+{OCAP}_{high}^{x}*{pc}_{high}^{x}}{{OCAP}_{low}^{x}*p_{low}^{x}+{OCAP}_{medium}^{x}*p_{medium}^{x}+ {OCAP}_{high}^{x}*p_{high}^{x}}\#(A3.9c) \end{aligned}$$

Here, ${IPD}_{low}^{x}$, ${IPD}_{medium}^{x}$, and ${IPD}_{high}^{x}$ are the Mangen et al. (2015) PD risk-group-specific IPD incidence rates for individuals aged $x$ years in the Netherlands. ${ICAP}_{low}^{x}$, ${ICAP}_{medium}^{x}$, and ${ICAP}_{high}^{x}$ are the analogous Mangen et al. (2015) inpatient CAP incidence rates, and ${OCAP}_{low}^{x}$, ${OCAP}_{medium}^{x}$, and ${OCAP}_{high}^{x}$ are the analogous Mangen et al. (2015) outpatientCAP incidence rates. The weights in the numerators of Equations A3.9a, A3.9b, and A3.9c are the age- and risk group-specific prevalence in the comorbid subpopulation as reported in Table S5; and the weights in the denominators are the age- and risk group-specific prevalence in the general population as reported in Table S4. Thus, these scale factors are ratios of the weighted average of IPD incidence rates across the risk groups in the comorbid subpopulation to that in the general population. These scale factors are presented in Table 1 of the article.

### **A3.2.4. Vaccine-type vaccine efficacy**

Vaccine efficacy equals the percentage decline in the incidence of infection resulting from vaccination. For example, a vaccine efficacy of 0.75 would mean that the incidence rate faced by a vaccinated individual is 25% of that faced by an unvaccinated individual. Assuming vaccination does not reduce infection severity, the expected number of treatment episodes per infection is unaffected by vaccination. Therefore, vaccine efficacy also equals the percentage decline in treatment episode incidence resulting from vaccination.

In this subsection, we provide the methodology used to derive age- and time-specific vaccine-type vaccine efficacy rates for the general population and the comorbid subpopulation (henceforth, “vaccine efficacy” without qualification refers to vaccine-type vaccine efficacy).

#### **A3.2.4.i. Baseline vaccine-type vaccine efficacy in the general population, base case scenario**

We conduct our analyses under two different assumptions regarding vaccine-type vaccine efficacy, which we refer to as our “base case” analysis and our “age invariant” scenario. The “base case analysis” represents our central analysis, while the age-invariant scenario is in the nature of a scenario analysis or sensitivity analysis. For our base case analysis, baseline age-specific vaccine-type vaccine efficacy (by “baseline” we mean initial vaccine efficacy, prior to any waning) follows the van Werkhoven et al. (2014)^[[29]](#footnote-29)^ Cox-proportional hazard-based analysis of the Community-Acquired Pneumonia Immunization Trial in Adults (CAPITA)^[[30]](#footnote-30)^ study results. The van Werkhoven et al. (2014) analysis produces a baseline efficacy against vaccine-type CAP of about 0.80 in 50-year-olds, about 0.27 in 85-year-olds, and constant proportional decline in between. These results suggest that vaccine efficacy for any given age from 51 years-old onward is ${(27/80)}^{1/35} = 0.9694$ the efficacy of the previous age. The methodology also suggests a baseline efficacy against vaccine-type IPD of about 0.95 in 50-year-olds and 0.58 in 85-year-olds and constant proportional decline of ${(58/95)}^{1/35} = 0.9860$ per year. Thus, our age-specific baseline vaccine-type vaccine efficacies for CAP and IPD follow Equations A3.10.a and A3.10.b, respectively:

$$\begin{aligned} {ve}_{x}^{0,CAP}=0.80*{0.9694}^{x-50}\#\left( A3.10.a \right) \end{aligned}$$

$$\begin{aligned} {ve}_{x}^{0,IPD}=0.95*{0.9860}^{x-50}\#\left( A3.10.b \right) \end{aligned}$$

The vaccine-type vaccine efficacy rates that we derive following the van Werkhoven et al. (2014) methodology are not calculated for ages younger than 50 years-old; therefore, for our base case analysis we follow Mangen et al. (2015) in assigning the baseline vaccine-type vaccine efficacy calculated for 50-year-olds to individuals aged 18–49 years.^[[31]](#footnote-31)^

#### **A3.2.4.ii. Baseline vaccine-type vaccine efficacy in the general population, age-invariant scenario**

We also conduct a scenario analysis using the age-invariant vaccine-type vaccine efficacy values taken directly from the CAPITA study. We use the study’s per-protocol analysis figures, which give us a baseline vaccine efficacy of 45.0% against noninvasive CAP and 75.0% against IPD.^[[32]](#footnote-32)^ Hereafter, we refer to this analysis as the age-invariant baseline vaccine efficacy scenario.

#### **A3.2.4.iii. Waning**

Consistent with previous economic modeling of PCV13 Adult, we assume that vaccine efficacy is constant for the first five years immediately following vaccination, declines 5% annually for the next five years, declines 10% annually for the next five years, and then goes to zero thereafter. We follow the same pattern of waning efficacy for all manifestations of PD in our analysis.

#### **A3.2.4.iv. Baseline vaccine-type vaccine efficacy in the comorbid subpopulation**

The vaccine efficacy rates derived above for the general population apply to low- and medium-risk populations. Mangen et al. (2015) derive a different efficacy rate for high-risk populations. Because a large share of high-risk individuals exists in the comorbid subpopulation, ignoring this distinction potentially overstates vaccine efficacy in the comorbid subpopulation. We therefore follow the same strategy as Mangen et al. (2015), who write, “For subjects in the high-risk group, PCV13 [vaccine efficacy] against [vaccine-type] IPD and CAP (both inpatient and outpatient) was assumed to be 22% and 35% lower, respectively….”^^[[33]](#footnote-33)^,^[[34]](#footnote-34)^^ We use these percentages to scale vaccine efficacies that we assume apply to the low- and medium-risk populations to estimate vaccine efficacy in the high-risk population. Our baseline vaccine efficacy rates (i.e., efficacy in 2015) for the comorbid subpopulation are then a weighted average of the efficacy rates for medium- and high-risk individuals, where the weights are the prevalence of medium- and high-risk individuals in the comorbid subpopulation. Equations A3.11.a and A3.11.b state this mathematically.

$$\begin{aligned} {vec}_{x}^{CAP,0}={ve}_{x}^{CAP,0}{pc}_{medium,no high}^{x}+{ve}_{x}^{CAP,0}\left( 1-0.35 \right){pc}_{high}^{x}\#\left( A3.11.a \right) \end{aligned}$$

$$\begin{aligned} {vec}_{x}^{IPD,0}={ve}_{x}^{IPD,0}{pc}_{medium,no high}^{x}+{ve}_{x}^{IPD,0}\left( 1-0.22 \right){pc}_{high}^{x}\#\left( A3.11.b \right) \end{aligned}$$

Here, ${ve}_{x}^{CAP,0}$ and ${ve}_{x}^{IPD,0}$ are the baseline vaccine efficacies (either from our base case or age-invariant scenarios) from the general population, ${vec}_{x}^{CAP,0}$ and ${vec}_{x}^{IPD,0}$ are the corresponding vaccine efficacies for our comorbid subpopulation, and ${pc}_{medium,no high}^{x}$ and ${pc}_{high}^{x}$ correspond to the medium- and high-risk group prevalence rates, respectively, shown in Table S5.

Table S12 presents our calculations of ${vec}_{x}^{CAP,0}$ and ${vec}_{x}^{IPD,0}$ under both the base case and age-invariant baseline vaccine efficacy scenarios. As shown in Table S12, vaccine efficacy in the comorbid subpopulation, $vec$, varies by age even in the age-invariant efficacy scenario. The reason is that the relative share of high-risk individuals varies with age (see Table S5). However, the underlying prescaled efficacy rates for CAP (i.e., 0.45) and IPD (i.e., 0.75) do not vary with age under this scenario.

**Table S12. Calculated vaccine efficacy against CAP and IPD in the comorbid subpopulation**

|  | Base case scenario | | Age-invariant baseline vaccine efficacy scenario | |
| --- | --- | --- | --- | --- |
| Age | $\boldsymbol{vec}_{\boldsymbol{x}}^{\boldsymbol{CAP,0}}$ | $\boldsymbol{vec}_{\boldsymbol{x}}^{\boldsymbol{IPD,0}}$ | $\boldsymbol{vec}_{\boldsymbol{x}}^{\boldsymbol{CAP,0}}$ | $\boldsymbol{vec}_{\boldsymbol{x}}^{\boldsymbol{IPD,0}}$ |
| 20 | 0.77 | 0.93 | 0.44 | 0.73 |
| 25 | 0.77 | 0.93 | 0.44 | 0.73 |
| 30 | 0.78 | 0.93 | 0.44 | 0.74 |
| 35 | 0.78 | 0.93 | 0.44 | 0.74 |
| 40 | 0.77 | 0.93 | 0.43 | 0.73 |
| 45 | 0.77 | 0.93 | 0.43 | 0.73 |
| 50 | 0.76 | 0.92 | 0.43 | 0.73 |
| 55 | 0.65 | 0.86 | 0.43 | 0.73 |
| 60 | 0.54 | 0.78 | 0.41 | 0.71 |
| 65 | 0.46 | 0.73 | 0.41 | 0.71 |
| 70 | 0.39 | 0.67 | 0.40 | 0.70 |
| 75 | 0.34 | 0.63 | 0.41 | 0.71 |
| 80 | 0.28 | 0.58 | 0.40 | 0.70 |
| 85 | 0.24 | 0.54 | 0.40 | 0.70 |

### **A3.2.5. Vaccine-type incidence rates for the vaccinated**

Equation A3.12 illustrates the formula for the vaccine-type incidence rates for the vaccinated $iv$, which vary by disease manifestation, age, and time $t$.

$$\begin{aligned} {iv}_{x}^{t,m}=\left( 1-\frac{{rve}^{t}}{100}{ve}_{x}^{0,m} \right)i_{x}^{t,m}\#\left( A3.12 \right) \end{aligned}$$

Here, $\text{rve}^{\text{ }\text{t}}$ stands for *relative vaccine efficacy* in year $t$, which reflects waning; $\text{ve}_{\text{x}}^{\text{0,m}}$ is baseline vaccine efficacy for disease manifestation $\text{m}$ and age $\text{x}$; and $\text{i}_{\text{x}}^{\text{ }\text{t,m}}$ is the vaccine-type incidence rate at age $\text{x}$ in year $\text{t}$ for disease manifestation $\text{m}$ for the unvaccinated. Note that due to waning vaccine efficacy, $\text{iv}_{\text{x}}^{\text{ }\text{t,m}\text{ }}\text{=}\text{ }\text{i}_{\text{x}}^{\text{ }\text{t,m}}$ for any $\text{t}\text{ }\text{>}\text{ }\text{14}$.

## **A3.3. Case fatality rates and probabilities of disability**

If an individual becomes infected with PD at chance node B, then he or she faces the risk of either death, short-term disability, or long-term sequelae depending on the manifestation. This subsection describes how we derive these probabilities for the general population and the comorbid subpopulation.

### **A3.3.1. Community-acquired pneumonia case fatality rates in the general population and comorbid subpopulation**

We obtain an inpatient CAP case fatality rate (CFR) from a 2018 study by Akyil et al. (2018)^[[35]](#footnote-35)^ on hospitalized adult patients (i.e., 18 years and older) with CAP as recorded in the pneumonia database of the Turkish Thoracic Society between 2011 and 2013. We take the inpatient CAP CFR to be the study’s 30-day mortality rate of 9.2%. We assume that the outpatient CAP CFR is equal to zero.

### **A3.3.2. Invasive pneumococcal disease case fatality rates in the general population and comorbid subpopulation**

We construct bacteremia and meningitis CFRs using the 2016 data from the ECDC *Surveillance Atlas of Infectious Diseases* that we describe in Section A3.2.1.ii. As discussed in that section, Turkey is not in this database, thus we construct our bacteremia and meningitis CFRs for Turkey by aggregating the counts of IPD-related cases and deaths across the six comparator countries presented in Table S6.

We take our bacteremia CFR to be the ratio of deaths to cases of bacteraemic pneumonia and septicemia combined. Looking at the counts of deaths and cases aggregated across our six comparator countries in Table S10 above, this equals (62.08+69.11)/(298.00+273.25) = 0.230 for ages 5-64 and (95.96+78.95)/(346.18+255.08) = 0.291 for ages 65 and up. We take our meningitis CFR to be the ratio of deaths to cases in the “meningitis” row of Table S10. This equals 15.96/176.34 = 0.091 for ages 5-64, and 21.94/75.07 = 0.292 for ages 65 and up.

The above process allows us to calculate a single bacteremia CFR and a single meningitis CFR for the broad age group 5-64 (0.230 and 0.091, respectively). However, there is likely to be age-variation in these CFRs within that broad age group. We impute such age-variation on the basis of the age distribution in deaths and cases of IPD across our comparator countries from the ECDC data. Unfortunately, these IPD distributions are not disaggregated by manifestation—or else we would rely on these data directly—so our imputation method below assumes that the age variation in bacteremia deaths and cases is the same as that in meningitis.

ECDC Surveillance Atlas provides for each comparator country the percentages of IPD-related deaths and cases in the narrower age groups 5-14, 15-24, 25-44, and 45-64 (see Columns 2 to 7, and Columns 2’ to 7’ of Table S13). (Note that these do not sum to 100% because we have omitted ages 4 and below and 65 and up.) We take a weighted average of these percentages across our comparator countries, where the weights correspond to each country’s share of bacteremia and meningitis deaths and cases in the broad age group 5-64 across all the countries. For example, from Table S9 the Czech Republic’s weight on the age structure of death is 27.97/147.14=0.19. We then rescale these percentages such that they sum to 100%, which yields Columns 8 and 8’ in Table S13. Thereafter, we impose the resulting age structures in Columns 8 and 8’ of Table S13 on the number of bacteremia deaths and cases, respectively, within the broad age group 5-64 across all the comparator countries in Table S10, and similarly for meningitis deaths and cases. For example, the number of bacteremia deaths for ages 45-64 is 0.8114 x 131.18, which is approximately 106. The number of bacteremia cases for ages 45-64 is 0.668 x 571.24, which is approximately 382.

The bacteremia and meningitis CFRs for each of the narrower age groups follow naturally. For example, the bacteremia CFR for ages 45-64 is 106/382, which is approximately 0.28. The resulting CFRs are reported in Table 1 of the article.

| **Table S13. Imputation of bacteremia and meningitis age structures within ages 5-64 years** | | | | | | | |
| --- | --- | --- | --- | --- | --- | --- | --- |
| **Age** | **Distribution of all IPD deaths amongst age groups** | | | | | | **Age structure of bacteremia and meningitis deaths** |
| **(1)** | **(2)** | **(3)** | **(4)** | **(5)** | **(6)** | **(7)** | **(8)** |
|  | **Czech Rep.** | **Greece** | **Hungary** | **Poland** | **Slovakia** | **Slovenia** |  |
| 5-14 | 0% | 0% | 0% | 0% | 0% | 0% | 0.00% |
| 15-24 | 0% | 0% | 0% | 0% | 0% | 0% | 0.56% |
| 25-44 | 11% | 0% | 5% | 8% | 0% | 0% | 18.31% |
| 45-64 | 29% | 0% | 37% | 35% | 67% | 25% | 81.14% |
| Total |  |  |  |  |  |  | 100.00% |
| **Age** | **Distribution of all IPD cases amongst age groups** | | | | | | **Age structure of bacteremia and meningitis cases** |
| **(1')** | **(2')** | **(3')** | **(4')** | **(5')** | **(6')** | **(7')** | **(8')** |
|  | **Czech Rep.** | **Greece** | **Hungary** | **Poland** | **Slovakia** | **Slovenia** |  |
| 5-14 | 3% | 6% | 2% | 2% | 10% | 3% | 5.29% |
| 15-24 | 2% | 0% | 0% | 2% | 3% | 1% | 2.79% |
| 25-44 | 10% | 22% | 12% | 13% | 9% | 10% | 25.12% |
| 45-64 | 32% | 35% | 34% | 34% | 39% | 22% | 66.80% |
| Total |  |  |  |  |  |  | 100.00% |
| Data Source: 2016 ECDC Surveillance Atlas of Infectious Diseases (https://atlas.ecdc.europa.eu/public/index.aspx). | | | | | | | |

### **A3.3.3. Probabilities of disability for the general population and the comorbid subpopulation**

Inpatient CAP- and bacteremia-related treatment episodes result in either death or short-term temporary disability. Thus, temporary disability for inpatient CAP and bacteremia occur with probabilities equal to the complements of their respective CFRs. Outpatient CAP involves only temporary disability, which means that outpatient CAP has a CFR of zero and the probability of temporary disability, conditional on contracting outpatient CAP, is one.

For meningitis, we follow Van de Beek et al. (2004) who study inpatient bacterial meningitis in the Netherlands.^[[36]](#footnote-36)^ Following this study, we allow for five meningitis-related outcomes: death, persistent vegetative state, severe disability, moderate disability, and temporary disability. Severe disability is defined as disability (inability to work, volunteer, provide care, or perform housework) *and* long-term dependency (needs long-term care), while moderate disability is defined as disability *without* long-term dependency. Our approach to parameterizing the probabilities associated with each of these outcomes relies on Table 3 of the van de Beek et al. study, which provides the disaggregation of the outcomes at discharge according to the Glasgow Outcome Score (GOS). Table S14 reports some of the GOS results:

**Table S14. Probability of various meningitis outcomes**

| Outcome | Probability |
| --- | --- |
| Death (GOS = 1) | 0.30 |
| Persistent vegetative state (GOS = 2) | 0.01 |
| Severe disability (GOS = 3) | 0.05 |
| Moderate disability (GOS = 4) | 0.14 |
| Good recovery (GOS = 5) | 0.50 |

Data Source: Glasgow Outcome Score in van de Beek et al. (2004).

The values that Table S14 reports are problematic because their probabilities are age-invariant while other sources suggest that the probability of mortality and other adverse outcomes rise with age. To address this issue, we hold fixed across all ages the probabilities of a persistent vegetative state, severe disability, and moderate disability as given by van de Beek et al. (2004), which together sum to 20%. We then use our age-varying CFRs for meningitis derived in Section A3.3.2 as our probabilities of death in place of the age-invariant probability of death from van de Beek et al. (2004), with the residual probability representing the probability of temporary disability. The probabilities of each of the five possible outcomes associated with meningitis are reported in Table 1 of the article.

We assume that comorbidities have no impact on the probability of a persistent vegetative state, the probability of severe or moderate disability, or the probability of death.

## **A3.4. Probability of a non-PD death**

The first component probability in our Markov model is the probability of surviving a non-PD-related death at chance node A. We now derive the age-, comorbid status-, and time-dependent probabilities associated with that outcome.

### **A3.4.1. Mortality risks in the general population**

In what follows, $\text{t0}$ denotes time at $\text{t}\text{ }\text{=}\text{ }\text{0}$ (i.e., year 2015), and $\text{x0}$ denotes age at time $\text{t}\text{ }\text{=}\text{ }\text{0}$.

We obtain age-specific base-year all-cause mortality rates ${\text{M}\text{ }}_{\text{x}}^{\text{t0}}$ from Eurostat Life Tables for 2014.^[[37]](#footnote-37)^ We calculate non-PD-related mortality rates by subtracting PD-related mortality rates from all-cause mortality rates as shown in Equation A3.13:

$\begin{aligned} M_{x}^{t0,nPD}= M_{x}^{t0} -\left( i_{x}^{t0,ICAP}{cfr}_{x}^{ICAP}+ i_{x}^{t0,B}{cfr}_{x}^{B}+ i_{x}^{t0,M}{cfr}_{x}^{M} \right)\#\left( A3.13 \right) \end{aligned}$

In a standard continuous-time life table setting *(*i.e., outside of our discrete time Markov model framework), assuming deaths occur uniformly within a yearlong interval, the probability of a representative member of a cohort’s dying from non-PD-related causes between ages $\text{x}$ and $\text{x}\text{ }\text{+}\text{ }\text{1}$ can be approximated by Equation A3.14:

$$\begin{aligned} q_{x}^{t0,nPD}=\frac{M_{x}^{t0,nPD}}{\left( 1+\left( \frac{M_{x}^{t0,nPD}}{2} \right) \right)}\#\left( A3.14 \right) \end{aligned}$$

In such a setting, the conditional number of life years lived between ages $\text{x}$ and $\text{x}\text{ }\text{+}\text{ }\text{1}$ equals the area under the survival curve where the survival curve’s value at $\text{x}$ is normalized to 1 (where the conditionality is with respect to being alive at age $\text{x}$). If we compute this survival curve such that it incorporates only non-PD deaths, then this area can be interpreted as the conditional number of life years not lost to non-PD-related deaths between ages $\text{x}$ and $\text{x}\text{ }\text{+}\text{ }\text{1}$. A linear approximation to this area is provided by Equation A3.15:

$$\begin{aligned} \left( \frac{1}{2} \right)*\left( 1+\left( 1-q_{x}^{t0,nPD} \right) \right)=1-\left( \frac{q_{x}^{t0,nPD}}{2} \right)\#\left( A3.15 \right) \end{aligned}$$

The complement of this area is $\text{q}_{\text{x}}^{\text{ }\text{t0,nPD}}\text{/}\text{ }\text{2}\text{,}$ which we interpret as the number of life years between ages $\text{x}$ and $\text{x}\text{ }\text{+}\text{ }\text{1}$ lost to non-PD death. We take this continuous time quantity as our discrete time approximation to the probability of a non-PD-related death.

### **A3.4.2. Mortality risks in the comorbid subpopulation**

To account for the impact of comorbidity status on mortality rates, we use data on diabetics’ mortality risks and apply them to the entire comorbid subpopulation. We begin by obtaining gender-specific mortality risk ratios (MRRs) from the International Diabetes Federation (IDF).^^[[38]](#footnote-38)^^ As the IDF only provides these data in 10-year age groups from age 20 through 79 years, we use linear interpolation to extrapolate the MRRs to ages 18-19 years. We compute the average values presented in Table S15 as a weighted average of the gender-specific MRRs using weights that are proportional to the male and female shares of each age group in the general Turkish population.^^[[39]](#footnote-39)^,^^[[40]](#footnote-40)^ Table S15 reports the population-weighted average MRRs.

**Table S15. Calculated MRRs of diabetics by**

**gender and age**

| Age | Males | Females | Weighted average |
| --- | --- | --- | --- |
| 18-19 | 4.11 | 6.90 | 5.48 |
| 20-29 | 3.66 | 6.05 | 4.84 |
| 30-39 | 3.38 | 5.41 | 4.40 |
| 40-49 | 1.85 | 3.14 | 2.51 |
| 50–59 | 1.63 | 2.64 | 2.16 |
| 60–64 | 1.60 | 2.04 | 1.84 |
| 65-69 | 1.60 | 2.04 | 1.84 |
| 70-74 | 1.39 | 1.79 | 1.61 |
| 75-79 | 1.39 | 1.79 | 1.61 |
| 80-84 | 0.94 | 0.94 | 0.94 |
| 85 | 0.94 | 0.94 | 0.94 |

We create the background mortality rates that comorbid individuals face by multiplying the MRRs in Table S15 by the mortality rates faced by the general population. The resulting mortality risks are those that comorbid individuals face even if they are not infected with PD. We then apply these mortality rates to Equation A3.14 to derive the probability of a non-PD death in the comorbid subpopulation.

# A4. State utilities as indirect costs

The Markov states in Figure 1 include the uninfected state; death from non-PD-related causes; death from inpatient CAP, bacteremia, or meningitis; temporary disability (TD) from inpatient CAP, outpatient CAP, bacteremia, and meningitis; and persistent vegetative state (PVS), severe disability (SD), and moderate disability (MD) from meningitis. The state utilities associated with the various PD-related Markov states consist of the productivity-based indirect costs associated with each state. Therefore, the uninfected state and death from non-PD-related causes are associated with zero state utilities, and all other states have positive state utilities (where the larger the state utility, the worse is the state). Indirect costs from death or disability following infection equal the expected present discounted value of lifetime losses in the infected individual’s paid and unpaid work and in caregivers’ paid work. In this section, we discuss the calculation of these indirect costs.

Categories of productivity loss include lost paid work and lost unpaid work, the latter in the form of lost housework, caregiving, and volunteering. The work may be that of the infected individual or of the infected individual’s formal and informal caregivers. The value of lost work equals time lost multiplied by the value per unit of time. We value paid work at the average pretax wage across all occupations (“average wage”), adjusted for the probability of employment. We value the infected individual’s unpaid work at its replacement cost (i.e., the cost of hiring someone to do the work) rather than at its opportunity cost (i.e., foregone earnings).^[[41]](#footnote-41)^ Based on this approach, we assume that the value of lost unpaid work is equal to the average pretax wage in the elementary occupations (“unskilled wage”). We value time spent by the infected individual’s caregivers at its opportunity cost equal to foregone paid work. We assume everyone in the infected individual’s social network is equally willing and able to miss work to provide care, thus we value this foregone paid work at the average wage across all adults.

The Markov states of our disease model include the uninfected state and death from non-PD-related causes; death from inpatient CAP, bacteremia, and meningitis; temporary disability from inpatient CAP, outpatient CAP, bacteremia, and meningitis; and PVS, severe disability, and moderate disability from meningitis. The uninfected state and death from non-PD-related causes are associated with no indirect costs. The indirect costs of death equal the expected present discounted value (PDV) of foregone lifetime paid and unpaid work. These costs are the same across inpatient CAP, bacteremia, and meningitis since we assume cause of death does not affect foregone lifetime productivity. Temporary disability includes periods of no productivity (i.e., absenteeism) and reduced productivity upon returning to work (i.e., presenteeism), which we value at the average wage. Temporary disability also includes informal caregivers’ foregone paid work, which we value at the average wage across all adults.

Meningitis-related PVS and severe disability each involve the loss of the infected person’s lifetime productivity. We assume that an individual who enters a PVS stays in that state for four years and dies thereafter. We derive this length based on a study by the Multi-Society Task Force on PVS^[[42]](#footnote-42)^ by computing the average months survived from this study, which is equal to (38.4 + 4.4 * 12 + 3.3 * 12 + 5.25 * 12) / 4. According to van de Beek et al. (2004),^[[43]](#footnote-43)^ both PVS and severe disability necessitate long-term care—comprising nursing, domestic, and informal care—for the duration of disability; however, severe disability involves disability for the rest of the individual’s expected lifetime. We value nursing care at the average pretax wage in the professional occupations (“professional wage”), which includes (among other jobs) medical and health services.^[[44]](#footnote-44)^ We conservatively value paid domestic care at the pretax wage in the elementary occupations (“unskilled wage”).^[[45]](#footnote-45)^ Because we assume that all potential informal caregivers are equally willing and able to miss work to provide care, we value informal care at the average wage across all adults.^[[46]](#footnote-46)^

We assume that moderate disability persists for two years based on a study by Weisfelt et al. (2006), which shows no role impairment difference between patients with Glasgow Outcome Scale 4 at discharge and patients with Glasgow Outcome Scale 5 at discharge after an average duration of 29.3 months.^[[47]](#footnote-47)^ Based on Schmand et al*.*(2010), we assume that subsequent good recovery is lifelong.^[[48]](#footnote-48)^ Based on definitions of Glasgow Outcome Scale outcomes,^[[49]](#footnote-49)^ we associate the two years of disability with an inability to do paid or unpaid work. However, these definitions also indicate that, unlike PVS and severe disability, moderate disability does not necessitate long-term care.

Constructing indirect costs depends on measuring the following set of economic and time use variables: employment rates, annual and hourly earnings, and annual and daily hours spent doing various types of paid and unpaid work. We do this in subsection A4.1. We compute indirect costs of death in subsection A4.2; indirect costs associated with meningitis-related PVS, severe disability, and moderate disability in subsection A4.3; and indirect costs of temporary disability in subsection A4.4.

### **A4.1. Economic data inputs**

#### **A4.1.1. Employment rates**

We obtain our employment rates from 2015 Eurostat labor force survey data.^[[50]](#footnote-50)^ Throughout our analysis, the employment rate refers to the ratio of employed individuals to the number of individuals in the population, rather than the ratio of employed individuals to the number of individuals in the labor market. Our calculations of the indirect costs of PD also use the average employment rate for adults of all ages. This average employment rate is obtained by filtering the age code variable in the Eurostat data to “Y_GE15.” This value in 2015 is 0.46.

#### **A4.1.2. Earnings**

We obtain annual earnings data^[[51]](#footnote-51)^ and hourly earnings data^[[52]](#footnote-52)^ from 2014 survey data available from Eurostat. These earnings data come averaged across gender, age group, occupation, and establishment size. Our analysis requires average earnings over all establishment sizes. However, the Eurostat earnings data for Turkey are based on establishments with 10 or more employees. To calculate the average earnings over all establishments for Turkey, we use the Eurostat earnings data for comparator countries for which the relevant data are available to calculate ratios of age-specific average earnings over all establishments to age-specific average earnings over establishments with 10 or more employees. We then calculate a simple average of these ratios over the comparator countries. We calculate the average earnings over all establishments for Turkey as the product of the Turkey average earnings for 10 or more establishments and the age-specific ratios described above. The comparator countries that we use to construct these ratios are Bulgaria, Czech Republic, Cyprus, Hungary, Poland, Slovenia, and Slovakia. We selected these countries on the basis of geographical proximity to Turkey, similarity in levels of socio-demographic development as measured by the SDI from the Global Burden of Disease, and similarity in per capita GDP (denominated in current 2016 international (PPP) dollars) from the World Development Indicators (WDI) from the World Bank.^[[53]](#footnote-53)^

The measurements of earnings (both hourly and annual) also need to be adjusted to account for the fact that the Eurostat hourly and annual earnings data reflect only the earnings of those with direct employment contracts, while our calculations of the shadow values of a unit of time spent doing some productive activity apply to all individuals.^^[[54]](#footnote-54)^^ Since the Eurostat data do not reflect the earnings of those who are employed but do not have direct employment contracts, such as the self-employed or unpaid family workers, they potentially overstate average earnings to the extent that the earnings of those without direct contracts are relatively lower on average compared to those with direct contracts. To correct this potential source of bias in the earnings data, we rely on income information by employment status from the 2014 Income and Living Conditions Survey that we obtain from the Turkish Statistical Institute (TurkStat)^^[[55]](#footnote-55)^^ and report in Table S16:

**Table S16. Annual income and share of employed population^^[[56]](#footnote-56)^^ by employment status**

| Employment status | Annual income (2014 Turkish lira) | Percentage of employed in 2014 |
| --- | --- | --- |
| Regular employees | 19,772 | 66.0%* |
| Casual employees | 7,667 |  |
| Employer | 44,587 | 4.5% |
| Self-employed | 14,629 | 17.3% |
| Unpaid family workers | N/A | 12.2% |
| All employed except unpaid family workers | 19,051 | 87.8% |

Data Sources: Turkish Statistical Institute (TurkStat). Mean annual income at main job by employment status; Turkish Statistical Institute (TurkStat). Employment status.

*TurkStat presents only one aggregate value representing the share of both regular and casual employees among the employed.

In this table, regular employees are defined as those who “work for a definite wage that is determined independent from the sales or interests of the company in accordance with the written or verbal agreement with their employer.”^^[[57]](#footnote-57)^^ Thus, we infer that “regular employees” in Table S16 correspond to “employees with direct employment contracts” in Eurostat. We therefore take the 19,772 Turkish lira value in Table S16 as the TurkStat counterpart to Eurostat data on earnings among regular employees.

What we need for our analysis is average earnings across all types of employment status in Table S16, not just earnings of regular employees. We calculate earnings across all types of employment by multiplying the Eurostat hourly and annual earnings data by the ratio of the average earnings across all employed workers to the earnings of regular employees in Table S16. To compute this ratio, we need to assign an earnings value to “unpaid family workers” in Table S16. According to the National Transfer Accounts (NTA), incomes reported by self-employed individuals typically reflect the joint efforts of the self-employed individual and of unpaid family workers.^^[[58]](#footnote-58)^^ If so, then the appropriate interpretation of the 14,629 lira in Table A16 is as the sum of the annual earnings of the average self-employed individual and the average unpaid family worker. To conform to this interpretation, we treat 14,629 lira as the earnings of the self-employed and nominally assign earnings of 0 lira to unpaid family workers. We then construct a weighted average of earnings across all employment categories in Table S16 as equal to 0.878 × 19,051 + 0.122 × 0 = 16,726.778 Turkish lira. The ratio of average earnings across all employed workers to earnings of regular employees is therefore equal to 16,726.778 / 19,772 = 0.846, or approximately 85%. Thus, we scale down the Eurostat hourly and annual earnings data by 100 – 85 = 15%.

##### **A4.1.2.i. Hourly earnings in the general population**

Our calculations use average hourly wages in the “elementary occupations,” the “professional occupations,” and across all occupations. The “elementary occupations” is a technical term used in the International Standard Classification of Occupations (ISCO) to refer to several unskilled manual occupations.^^[[59]](#footnote-59)^^ Hourly earnings in the elementary occupations are identified in the Eurostat data by a value of “OC9” for the ISCO08 code. The “professional occupations” is a technical term that ISCO uses to encompass (among other things) medical and health services.^^[[60]](#footnote-60)^^ Hourly earnings in the “professional occupations” are identified in the Eurostat data by a value of “OC2” for the ISCO08 code. Hourly earnings across all occupations are identified in the Eurostat data by a value of “TOTAL” for the ISCO08 code.

For each of these occupational categories (overall, elementary, and professional), the average hourly earnings for adults of all ages is obtained by filtering the raw Eurostat data by a value of “TOTAL” for the age code. These raw average hourly earnings are €3.84, €2.41, and €8.15 for the overall, elementary, and professional occupations, respectively. As we discuss above, we adjust these values for the fact that they are based on establishments with 10 or more employees by decreasing them by 5%, 1% and 4% for the overall, elementary, and professional occupations, respectively. As we also discuss above, we account for the fact that the Eurostat data only measure hourly earnings for employees with direct employment contracts by reducing them by an additional 15%. The final hourly earnings that we analyze are €3.10, €2.01, and €6.64 for the overall, elementary and professional occupations, respectively.

##### **A4.1.2.ii. Annual earnings for all occupations in the general population**

Table S17 provides the annual earnings based on Eurostat averages. Column 2 reports the raw Eurostat annual earnings. Column 4 adjusts these earnings for establishment size by multiplying them by the ratio of average earnings over all establishments to those over establishments with 10 or more employees from comparator countries, which we report in Column 3. In Column 5, we account for the fact that the Eurostat data only measure annual earnings for employees with direct employment contracts by reducing the earnings in Column 4 by additional 15%.

| **Table S17. Adjusted annual earnings by age group, Turkish general population (2014 €)** | | | | |
| --- | --- | --- | --- | --- |
| **(1)** | **(2)** | **(3)** | **(4)** | **(5)** |
| **Age** | **Raw Eurostat annual earnings** | **Ratio of earnings over all establishment to those over establishments with 10+ employees (from comparator countries)** | **Earnings adjusted for establishment size** | **Earnings adjusted for employment contract premium** |
|  | **(2014 euros)** |  | **Column (2)** × **Column (3)** | **Column (4)** × **0.85** |
| 18-29 | 7,701.00 | 95.58% | 7,360.53 | 6,226.88 |
| 30-39 | 11,043.00 | 94.64% | 10,450.61 | 8,841.04 |
| 40-49 | 11,879.00 | 94.45% | 11,219.33 | 9,491.37 |
| 50-59 | 12,147.00 | 95.32% | 11,578.29 | 9,795.04 |
| 60-85 | 19,090.00 | 94.46% | 18,032.35 | 15,255.06 |

We modify the annual earnings in Column 5 of Table S17 for those aged 60–85 years in an attempt to allow for more plausible age variation in earnings within this broad age group. To do this, we assume that an employed individual aged 60 years and older can either be employed full-time, with probability denoted by $\text{FT}$, or part-time with probability $\text{1}\text{ }\text{–}\text{ }\text{FT}$. Table S18 reports these probabilities:

**Table S18. Calculated probability of**

**full-time employment among employed**

**adults aged 60 and older by age, Turkish**

**general population^^[[61]](#footnote-61)^^**

| Age | Rate of full employment |
| --- | --- |
| 60–64 | 0.77 |
| 65–74 | 0.72 |
| 75–85 | 0.66 |
| Data Source: European Commission, Eurostat | |

We then allow a part-time employed individual of age $x$ to have annual earnings ${\text{Ann\_Earn}\text{ }}_{\text{ }\text{x}}^{\text{PT}}$ that are only a fraction of the earnings of a similarly aged full-time employed individual, ${\text{Ann\_Earn}\text{ }}_{\text{x}}^{\text{FT}}$. We do so for two reasons: first, part-time employees work fewer hours than full-time employees, and second part-time employees may be paid on a different salary scale than full-time employees. If we measure the first factor using the ratio of part-time hours worked to full-time hours worked (denoted by $\text{PH}\text{ }\text{/}\text{ }\text{FH}$) and the second factor using the ratio of part-time earnings in full-time equivalents to full-time earnings (denoted by $\text{PTE\_FTE}\text{ }\text{/}\text{ }{\text{Ann\_Earn}\text{ }}_{\text{x}}^{\text{FT}}$, where $\text{PTE\_FTE}$ equals the amount a part-time employee would have earned if she/he worked full-time hours at her/his current hourly earnings rate), then Equation A4.1 illustrates the relationship between part-time earnings and full-time earnings:

$$\begin{aligned} {Ann\_Earn}_{x}^{PT}=\left( {PH}/{FH} \right)(PTE\_FTE/{Ann\_Earn}_{x}^{FT}){Ann\_Earn}_{x}^{FT}\#\left( A4.1 \right) \end{aligned}$$

To simplify our notation, we define the fraction $\text{K}\text{ }\text{=}\text{ }\left( \text{PH}/{\text{ }\text{FH}} \right)\text{ }\text{(PTE\_FTE}\text{ }\text{/}{\text{Ann\_Earn}\text{ }}_{\text{x}}^{\text{FT}}\text{)}$, which

according to the data reported in Table S19 is equal to 0.60.

**Table S19. Weekly work hours and annual earnings for adults**

**aged 60 and older by work status, Turkish general population**

| Work status | Weekly work hours^[[62]](#footnote-62)^ | Annual earnings (€2014)^[[63]](#footnote-63)^ |
| --- | --- | --- |
| Full-time | 50.9 ($FH$) | 19,010 ($\text{Ann\_Earn }_{\text{x}}^{\text{FT}}$) |
| Part-time | 19.1 ($PH$) | 30,532* ($\text{PTE\_FTE}$) |

Data source: European Commission, Eurostat

*Full-time equivalent rate, or how much a part-time worker would have earned annually

if he or she worked full-time hours at her/his current earnings per hour

By definition, the annual earnings among those aged $x$ is equal to the weighted average of earnings of the full-time employed and part-time employed of that age, as Equation A4.2 shows:

$${Ann\_Earn}_{x}=FT*{Ann\_Earn}_{x}^{FT}+\left( 1-FT \right)*{Ann\_Earn}_{x}^{PT}$$

$$=FT*{Ann\_Earn}_{x}^{FT}+\left( 1-FT \right)*K*{Ann\_Earn}_{x}^{FT}$$

$$\begin{aligned} =[FT+\left( 1-FT \right)K]{Ann\_Earn}_{x}^{FT}\#\left( A4.2 \right) \end{aligned}$$

Also by definition, the annual earnings among those aged 60 years and older, ${Ann\_Earn}_{60+}$, is equal to the weighted average of annual earnings ${\text{Ann\_Earn}\text{ }}_{\text{x}}$ across the age groups $\text{x}\text{ = 60–64, 65–69, 70–74, 75–79, 80–84}$, where the weight for a particular age group (e.g., $W_{60-64})$ is the ratio of the number of full-time employed within that age group (equal to the product of the full-time employment rate in that age group and the population size of that age group) to the number full-time employed across all age groups (i.e., the number of employed aged 60-84) as shown in Equation A4.3:

$${Ann\_Earn}_{60+}=W_{60-64}{Ann\_Earn}_{60-64}+W_{65-69}{Ann\_Earn}_{65-69}+W_{70-74}{Ann\_Earn}_{70-74}+W_{75-79}{Ann\_Earn}_{75-79}$$

$$\begin{aligned} + W_{80-84}{Ann\_Earn}_{80-84}\#\left( A4.3 \right) \end{aligned}$$

Combining these two equations yields Equation A4.4:

$${Ann\_Earn}_{60+}=W_{60-64}\left[ {FT}_{60-64}+\left( 1-{FT}_{60-64} \right)K \right]{Ann\_Earn}_{60-64}^{FT}+$$

$$W_{65-69}[{FT}_{65-69}+\left( 1-{FT}_{65-69} \right)K]{Ann\_Earn}_{65-69}^{FT}+$$

$$W_{70-74}[{FT}_{70-74}+\left( 1-{FT}_{70-74} \right)K]{Ann\_Earn}_{70-74}^{FT}+$$

$$W_{75-79}\left[ {FT}_{75-79}+\left( 1-{FT}_{75-79} \right)K \right]{Ann\_Earn}_{75-79}^{FT}+$$

$$\begin{aligned} W_{80-84}\left[ {FT}_{80-84}+\left( 1-{FT}_{80-84} \right)K \right]{Ann\_Earn}_{80-84}^{FT}\#\left( A4.4 \right) \end{aligned}$$

A final issue to address is how full-time annual earnings (on the right-hand side of Equation A4.4) vary with respect to age. To the extent that age is negatively correlated with physical and mental health, it is also negatively correlated with earnings within the retirement population. However, since highly productive individuals tend to receive a higher return to working, as workers age, those that are high-income earners may have a relatively stronger incentive to remain employed compared to their low-income counterparts. This self-selection bias would tend to make earnings positively correlated with age within the retirement population. We assume that these two effects offset each other and that full-time annual earnings are age-invariant among those aged 60 and older as shown in Equations A4.5.a–A4.5.d:

$$\begin{aligned} {Ann\_Earn}_{65-69}^{FT}={Ann\_Earn}_{60-64}^{FT}\#\left( A4.5.a \right) \end{aligned}$$

$$\begin{aligned} {Ann\_Earn}_{70-74}^{FT}={Ann\_Earn}_{60-64}^{FT}\#\left( A4.5.b \right) \end{aligned}$$

$$\begin{aligned} {Ann\_Earn}_{75-79}^{FT}={Ann\_Earn}_{60-64}^{FT}\#\left( A4.5.c \right) \end{aligned}$$

$$\begin{aligned} {Ann\_Earn}_{80-84}^{FT}={Ann\_Earn}_{60-64}^{FT}\#\left( A4.5.d \right) \end{aligned}$$

Our expression for ${\text{Ann\_Earn}\text{ }}_{\text{60+}}$ therefore becomes that shown in Equation A4.6:

$${Ann\_Earn}_{60+}=W_{60-64}\left[ {FT}_{60-64}+\left( 1-{FT}_{60-64} \right)K \right]{Ann\_Earn}_{60-64}^{FT}+$$

$$W_{65-69}[{FT}_{65-69}+\left( 1-{FT}_{65-69} \right)K]{Ann\_Earn}_{60-64}^{FT}+$$

$$W_{70-74}[{FT}_{70-74}+\left( 1-{FT}_{70-74} \right)K]{Ann\_Earn}_{60-64}^{FT}+$$

$$W_{75-79}\left[ {FT}_{75-79}+\left( 1-{FT}_{75-79} \right)K \right]{Ann\_Earn}_{60-64}^{FT}+$$

$$\begin{aligned} W_{80-84}\left[ {FT}_{80-84}+\left( 1-{FT}_{80-84} \right)K \right]{Ann\_Earn}_{60-64}^{FT}\#\left( A4.6 \right) \end{aligned}$$

Note that every quantity in Equation A4.6 is given but for the unknown ${\text{Ann\_Earn}\text{ }}_{\text{60-64}}^{\text{FT}}$. Therefore, Equation A4.6 allows us to compute the value of this unknown. Given this value, we use Equation A4.7 to compute the values of ${\text{Ann\_Earn}\text{ }}_{\text{60-64}\text{ }}\text{,…,}\text{ }{\text{Ann\_Earn}\text{ }}_{\text{80-84}\text{ }}$:

$${Ann\_Earn}_{x}={FT}_{x}*{Ann\_Earn}_{x}^{FT}+\left( 1-{FT}_{x} \right)*{Ann\_Earn}_{x}^{PT}$$

$$={FT}_{x}*{Ann\_Earn}_{x}^{FT}+\left( 1-{FT}_{x} \right)*K*{Ann\_Earn}_{x}^{FT}$$

$$\begin{aligned} =[{FT}_{x}+\left( 1-{FT}_{x} \right)K]{Ann\_Earn}_{60-64}^{FT}\#\left( A4.7 \right) \end{aligned}$$

##### **A4.1.2.iii. Productivity for the comorbid subpopulation**

To account for the likelihood that comorbid adults have lower annual and daily earnings than adults in the general population, we use data related to diabetics and apply the resulting scale factors to all comorbid individuals in our program population. To this end, we rely on a study by Sortsø et al*.* (2016)^[[64]](#footnote-64)^ on the socioeconomic costs of diabetes in Denmark that measures the following three categories of productivity losses associated with diabetes:

1. The impact of diabetes on annual gross income, reflecting among other things its impact on occupational category, employment rates, and early retirement. This impact is measured by the difference in annual gross income between a diabetic population aged 15–69 years and controls matched by gender, educational attainment, and five-year age category. When averaging these differences across all the matched subsamples, Sortsø et al*.* (2016) find that, on average, diabetics’ annual income is 3,045 euros lower than that of nondiabetics.
2. The productivity loss from premature mortality.
3. The productivity loss from absenteeism, which is the annual number of days absent from work because of diabetes estimated at three days and a value of 367 euros.

We use the Sortsø et al*.* (2016) categories (i) and (iii) to generate an earnings scale factor that we interpret as the “penalty” that diabetes imposes on expected annual earnings (including its expected impact on the likelihood of being employed). This scale factor is expressed in Equation A4.8:

$$\begin{aligned} {SF}_{annual earnings}=\frac{{Ann\_Earn}_{15+}*{ER}_{15+}-€(3,045+367)}{{Ann\_Earn}_{15+}*{ER}_{15+}}\#\left( A4.8 \right) \end{aligned}$$

In this expression, $\text{Ann\_Earn}_{\text{15+}}\text{* }\text{ER}_{\text{15+}}$ is the expected annual earnings across employed and unemployed aged 15 and older in Denmark using Eurostat data for Denmark. (We use Eurostat data for Denmark instead of Turkey, as Denmark is the source of this scale factor.) The second term in the numerator is the sum of the average difference in annual earnings between the diabetic and nondiabetic populations and the value of productivity loss because of diabetes-related absenteeism.^^[[65]](#footnote-65)^^ The computed earnings scale factor is equal to 0.89. We take annual and daily earnings in the comorbid subpopulation to equal the corresponding values in the general population multiplied by this scale factor.

Literature on the impact of comorbidities on non-market productivity is scarce.^[[66]](#footnote-66),^^[[67]](#footnote-67)^ Therefore, we assume that comorbidities do not have any impact on non-market productivity. In summary, we assume that, in the absence of PD, the comorbid subpopulation has 0.89 the market productivity of the general population and the same nonmarket productivity as the general population.

#### **A4.1.3. Time use**

The productivity losses in our model include the values of lost labor market earnings and lost time spent housekeeping, caregiving, and volunteering. We now describe the creation of the time-use variables that we use in our calculations of these values.

##### **A4.1.3.i Market time use**

The indirect costs calculations use the mean daily hours worked per employed individual. This value is estimated using 2015 labor market survey data from Eurostat on mean weekly hours worked.^^[[68]](#footnote-68)^^ In 2015 in Turkey, this value was 47.4. We divide these weekly hours by 5 to get the mean daily hours worked of 9.48.

##### **A4.1.3.ii. Non-market time use**

The data sources for non-market time are from TurkStat^^[[69]](#footnote-69)^^ and the Organisation for Economic Cooperation and Development (OECD).^^[[70]](#footnote-70)^^ The TurkStat data are age-specific, while the OECD data are not age-specific. In line with OECD definitions,^[[71]](#footnote-71)^ we take volunteering to be half of the TurkStat time use category “Volunteer Work and Meetings.” TurkStat provides age-specific time spent in a broad activity called “Household and family care.” We break up this broad activity into two sub-activities, “household work” and “family care,” as follows: The OECD reports time spent on household work and family care separately. These data indicate that 17.25% of this time is spent on informal care and the remainder on housework. We therefore assume that 17.25% of “Household and family care” in the TurkStat dataset consists of informal care, and the remainder is housework. For all time use categories, we apply the TurkStat values for the age group starting at age 55 to our entire age 65–85 elderly subpopulation.

### **A4.2. Indirect costs of death**

The indirect costs of death equal the expected present discounted value of foregone lifetime paid and unpaid work. These costs are the same across inpatient CAP, bacteremia, and meningitis since we assume that the cause of death does not affect foregone lifetime productivity.

Our calculations of the expected lifetime market- and non-market-based productivity losses resulting from infection assume that an individual who is alive during a particular year can participate in four productive activities: paid work, housework, volunteering, and caregiving. Therefore, the indirect costs of death center on the loss of a lifetime’s worth of these four productive activities. Also, during any particular year, a healthy individual (i.e., one who is not infected with PD) faces the background mortality risks of the general population. For every healthy individual of age $x$ at time $t$, we can compute this individual’s expected lifetime productivity. We calculate the expected lifetime productivity as the sum across all future years of life of the probability of being alive in each year of life, multiplied by the expected time spent in productive work conditional on being alive, and multiplied in turn by the shadow value of production per unit of time. These future years are discounted at rate $r$, which we set to 3%.

#### **A4.2.1. Life table variables and formulas for the general population and the comorbid subpopulation**

To compute expected lifetime productivity requires constructing a life table. To construct this life table, we depart from the discrete time structure of Markov models and adopt a continuous time framework. In effect, we model the life that would have occurred otherwise (i.e., the life that would have occurred had death not occurred) in continuous time. This continuous-time modeling depends on an expression for discounted age-specific life expectancy, which we derive here. We take age-specific base-year mortality rates ${\text{M}\text{ }}_{\text{x}}^{\text{t0}}$ from Eurostat. In a standard continuous-time life table setting, assuming deaths occur uniformly within a yearlong interval, the probability of a representative member of a cohort’s dying from any cause between ages $\text{x}$ and $\text{x}\text{ }\text{+}\text{ }\text{1}$ can be approximated by Equation A4.9:

$$\begin{aligned} q_{x}=\frac{M_{x}^{t}}{1+\frac{M_{x}^{t}}{2}}\#\left( A4.9 \right) \end{aligned}$$

for $\text{x }\text{≥ }\text{18}\text{ }\text{and}\text{ }\text{x}\text{ }\text{< 85}$. We use this variable to generate a survival function:

$$\begin{aligned} l_{18}=1\#\left( A4.10 \right) \end{aligned}$$

$$\begin{aligned} l_{x}=l_{x-1}*\left( 1-q_{x-1} \right), 18<x\leq85\#\left( A4.11 \right) \end{aligned}$$

The number of life years lived between ages $\text{x}$ and $\text{x }\text{+ 1}$ equal to the area under the survival function between those ages, can be approximated linearly by:

$$\begin{aligned} L_{x}=\left( 0.5 \right)\left( l_{x}+l_{x+1} \right), 18\leq x<85\#\left( A4.12 \right) \end{aligned}$$

$$\begin{aligned} L_{85}^{t}=0.5*l_{85}^{t}\#\left( A4.13 \right) \end{aligned}$$

A guaranteed (i.e., non-risky) life of length $\text{∆}\text{y}$ lived, starting today but lived as a continuous stream and discounted at a rate $r$, has a continuous time PDV of $\int_{\text{0}}^{\text{∆}\text{y}} \text{1*}\text{e}^{\text{-}\text{r}\text{*}\text{a}}\text{da}\text{ }\text{=}\left( \text{1}/\text{r} \right)\text{(1-}\text{e}^{\text{-}\text{r}\text{*∆}\text{y}}\text{)}$.^^[[72]](#footnote-72)^^ And if this life is lived starting $\Delta t$ years in the future, then its PDV as of today is $\text{(}\text{e}\text{\textasciicircum(-}\text{r}\text{*∆}\text{t}\text{)}\text{ }\text{⁄}\text{ }\text{r}\text{)(1-}\text{ }\text{e}\text{\textasciicircum(-}\text{r}\text{*∆}\text{y}\text{))}$.

For example, consider an individual who at time $\text{t }\text{= 0}$ has his or her 45^th^ birthday and an age-specific life expectancy of 40 (i.e., he or she is expected to live until age 85). The empirical question we seek to answer is: What is the benefit to this individual on his or her 45^th^ birthday of a future stream of 40 additional years of life? To answer this question, we can think of this stream as a series of annual benefits equal to $\text{L}_{\text{x}}^{\text{0}}\text{ /}\text{ l}_{\text{45}}^{\text{0}}$ that start on the individual’s $\text{x}$th birthday and extend to his or her 85^th^ birthday. On the 45^th^ birthday, the benefit is $\text{L}_{\text{45}}^{\text{0}}\text{ / }\text{l}_{\text{45}}^{\text{0}}$, on the 46^th^ birthday it is $\text{L}_{\text{46}}^{\text{1}}\text{ / }\text{l}_{\text{45}}^{\text{0}}$, and so on until the 85^th^ birthday when it is equal to $\text{L}_{\text{85}}^{\text{85-45}}\text{ / }\text{l}_{\text{45}}^{\text{0}}$. Considering the benefit of one additional year of life of $\text{L}_{\text{45 }}^{\text{0}}\text{/ }\text{l}_{\text{45}}^{\text{0}}$ on the 45^th^ birthday and assuming this quantity of life is spread out continuously between the 45^th^ and 46^th^ birthdays, its PDV as of today is $\text{(}\text{L}_{\text{45}}^{\text{0}}\text{ / }\text{l}_{\text{45}}^{\text{0}}\text{)(}$1/r)$\text{(1-}\text{e}^{\text{-r*1}}\text{)}$. The benefit of $\text{L}_{\text{46}}^{\text{1}}\text{ / }\text{l}_{\text{45}}^{\text{0}}$ on the individual’s 46^th^ birthday has PDV as of today is $\text{(}\text{L}_{\text{46}}^{\text{1}}\text{ / }\text{l}_{\text{45}}^{\text{0}}\text{)}\left( {\text{e}^{\text{-r*1}}}/\text{r} \right)\text{(1-}\text{e}^{\text{-r*1}}\text{)}$. And so on. Thus, the PDV of a future stream of 40 additional years of life (i.e., the PDV of the stream of benefits, is $({((1/{r)*\left( 1-e^{-r*1} \right))}}/{l_{x}^{0}})\left( L_{x}^{0}+e^{-r*1}L_{x+1}^{1}+\ldots+e^{-r*\left( 85-x \right)}L_{85}^{85-x} \right)$. Thus, the PDV of a future stream of life is equal to:

$$\begin{aligned} \frac{\frac{1}{r}\left( 1-e^{-r*1} \right)}{l_{x}^{t}}\sum_{i=x}^{85} e^{-r*\left( i-x \right)}L_{i}^{t+\left( i-x \right)}\#\left( A4.14 \right) \end{aligned}$$

Equation A4.14 gives the continuous time formula for discounted age-specific life expectancy. We do not use such age-specific life expectancies directly, but we use this formula to measure the expected PDV over a lifetime of various economic variables below. The conceptually critical variable in this formula is $L_{x}^{t}$, which is equal to the amount of life that an individual is expected to live within some future year (e.g*.*, the year 2020). If the individual is sure to survive until the start of that year (i.e., January 1, 2020) and survive to the end of that year (i.e., December 31, 2020), then $\text{L}_{\text{x }}^{\text{t}}\text{= 1}$. However, this is unlikely ever to be the case because there is always some chance that he/she will die before January 1, 2020. Therefore, in general, the expected quantity of life an individual will live in any future year is less than one (i.e., $\text{L}_{\text{x}}^{\text{t}}\text{<1}$).

We calculate the discounted age-specific life expectancies for comorbid individuals by applying the comorbid-specific mortality rates that we construct in Section A3.4.2 to Equation A4.9.

#### **A4.2.2. Expected value of lifetime earnings**

The loss in lifetime earnings from paid work is the discounted sum across future years of expected yearly earnings, where expected yearly earnings is the product of the probability of being alive in that year, the probability of being employed conditional on being alive, and annual earnings conditional on being employed. We assume long-term technological progress makes workers more productive over time, in turn making annual earnings grow at a rate $g$ equal to the long-term growth rate of per capita gross domestic product (GDP).^^[[73]](#footnote-73)^^

Equation A4.15 gives the formula for loss in lifetime earnings:

$$\begin{aligned} {Val\_Life\_Earn\_Lost}_{x}=\frac{\frac{1}{r-g}(1-e^{(g-r)*1})}{l_{x}^{t}}\sum_{i=x}^{85} e^{(g-r)*(i-x)}{Ann\_Earn}_{i}^{0}{ER}_{i}^{t+(i-x)}L_{i}^{t+(i-x)}\#\left( A4.15 \right) \end{aligned}$$

In this formula, $\text{t}\text{ }\text{=}\text{ }\text{0 }$(i.e., year 2015). The left-hand side variable refers to the value of lifetime earnings lost due to premature mortality at age $\text{x}$ in time $\text{t}$ (i.e., 2015). In the right-hand side variable, ${\text{ER}\text{ }}_{\text{x}}^{\text{t}}$ is the age-specific employment rate in time $\text{t}$ and$\text{ }{\text{Ann\_Earn}\text{ }}_{\text{x}}^{\text{0}}$ is the age-specific annual earnings among the employed in the base year (i.e*.*, 2015). Note that given our assumption that age-specific employment rates do not vary with time, the variable ${\text{ER}\text{ }}_{\text{x}}^{\text{t}}$ can be replaced with ${\text{ER}\text{ }}_{\text{x}}^{\text{0}}$ in Equation A4.15.

#### **A4.2.3. Growth rate of per capita GDP**

Note that the discount rate is now $\text{r}\text{ }\text{–}\text{ }\text{g}$. Setting this new rate is equivalent to assuming that annual earnings $\text{t}\text{ }\text{>}\text{ }\text{0}$ years into the future are $\text{e}^{\text{ }\text{gt}}$ times larger than annual earnings in the base year, ${\text{Ann\_Earn}\text{ }}_{\text{x}}^{\text{0}}$ . Our general approach to estimating the growth rate $\text{g}$ is to obtain two historical values of per capita GDP spaced sufficiently far apart in time and then calculate the rate of growth under the assumption that the later value grew from the earlier value continuously at an implied annual rate of $\text{g}$. Applying this method to Turkey, we find that the resulting estimate is highly sensitive to our choice of the time horizon due to volatility in the historic growth rates. For example, using IMF data, $g$ varied by more than a full percentage point between a 20-year estimate (i.e., between 1996 and 2016) and a 15-year estimate (i.e., between 2001 and 2016). This volatility is likely due to the massive effects of the 2001 Turkish economic crisis, wherein Turkey experienced years of negative growth.

To insulate against such effects, we pool growth rate estimates across 10 countries who are similar to Turkey with respect to 1995 per capita GDP and population, where 1995 is the beginning of our reference period. Specifically, we implement the following steps:

1. We use data on per capita GDP denominated in constant international purchasing power parity dollars from the WDI.^^[[74]](#footnote-74)^^
2. We use a 20-year interval to calculate a growth rate. This time horizon is sufficiently long to insulate against the effects of outlier years and short-term macroeconomic fluctuations, but sufficiently short to ensure that the historical economies do not differ from their present-day counterparts in economically relevant ways. Because the latest year of WDI per capita GDP data for most countries is 2015, we compute the average annual growth rate from 1995 forward.
3. To find nine comparator countries, we first rank countries by their per capita GDP in 1995. We then choose the four countries whose per capita GDP ranks immediately above Turkey and the five countries that rank immediately below Turkey. In generating this list, we exclude countries with populations below 4,000,000 in 1995 (per UN Population Prospects data^[[75]](#footnote-75)^) under the assumption that the economies in these countries are not sufficiently comparable to Turkey’s economy. The resulting countries are Mexico, Russian Federation, Croatia, Iran, Turkey, Chile, Poland, Brazil, Romania, and Thailand.
4. To calculate a 20-year average growth rate in each country, we use earlier and later values of per capita GDP given by ${pc\_gdp}_{0}$ and ${pc\_gdp}_{20}$, respectively, spaced 20 years apart. We model economic growth using an exponential growth function, as expressed in Equation A4.16:

$$\begin{aligned} {pc\_gdp}_{20}=e^{gt}{pc\_gdp}_{0}\#\left( A4.16 \right) \end{aligned}$$

This in turn gives us Equation A4.17:

$$\begin{aligned} g=\frac{1}{20}ln(\frac{{pc\_gdp}_{20}}{{pc\_gdp}_{0}})\#\left( A4.17 \right) \end{aligned}$$

1. We take a simple average of these 10 country-specific growth rates, which generates a value of 2.61% for $g$.

Note that our expression for the indirect costs of death depends on a continuous-time formula for discounted age-specific life expectancy. This formulation of time treats a death that occurs during a particular year of life as occurring at the initial moment of the interval (e.g., at 12:01 am of January 1), treats any production that would have occurred (had death not happened) during that cycle as occurring (like life itself) in a continuous stream throughout the interval, and therefore discounts that productivity using the same continuous-time discounted formula as for life expectancy.

#### **A4.2.4. Expected values of lifetime housework, caregiving, and volunteering**

We calculate the values of expected lifetime housework, caregiving, and volunteering following our methodology for calculating the value of expected lifetime earnings. The time-use and earnings variables that we use in these calculations are described in Section A4.1.3 and A4.1.2, respectively. We denote time spent per year on housework, caregiving, and volunteering for an individual of age $\text{x}$ at time $\text{t}$ by ${\text{AH\_Hwork}\text{ }}_{\text{x}}^{\text{t}}$ , ${\text{AH\_care}\text{ }}_{\text{x}}^{\text{t}}\text{ }$, and ${\text{AH\_vol}\text{ }}_{\text{x}}^{\text{t}}$ , respectively, where “$\text{AH}$” stands for “annual hours.”

We adopt a conservative replacement cost approach to the shadow values. That is, we value these units of time based on how much it would have cost the beneficiaries of these activities to pay someone else to perform them. We conservatively estimate this cost at the hourly earnings in the elementary occupations ${\text{Hour\_Earn}\text{ }}_{\text{x}}^{\text{EO,0}}$, where the superscript stands for the hourly earnings in the elementary occupations in the base year (i.e., $t=0$) and the subscript refers to age. We assume that these earnings grow at the same rate as per capita GDP. In what follows, we refer to these hourly earnings as the shadow value of the time spent on housework, caregiving, and volunteering.

Note that the formulas below for the values of expected lifetime housework, caregiving, and volunteering have the same structure as the formula for discounted age-specific life expectancy, except that $\text{L}_{\text{x}}^{\text{t}}$ is premultiplied by the product of the annual hours spent on an activity and the shadow value of that activity.

Equation A4.18 gives the value of lifetime housework lost due to PD:

$${Val\_Life\_Hwork\_Lost}_{x}=$$

$$\begin{aligned} \frac{\frac{1}{r-g}(1-e^{(g-r)*1})}{l_{x}^{t}}\sum_{i=x}^{85} e^{(g-r)*(i-x)}{Hour\_Earn}_{i}^{EO,0}{AH\_Hwork}_{i}^{t+(i-x)}L_{i}^{t+(i-x)}\#\left( A4.18 \right) \end{aligned}$$

In this formula, $\text{t}\text{ }\text{=}\text{ }\text{0 }$(i.e., year 2015). Note that given our assumption that age-specific hours of housework do not vary with time, the variable ${\text{AH\_Hwork}\text{ }}_{\text{x}}^{\text{t}}$ can be replaced with ${\text{AH\_Hwork}\text{ }}_{\text{x}}^{\text{0}}$ in Equation A4.18.

Equation A4.19 gives the value of lifetime caregiving lost due to PD, which is the value of caregiving that an individual would have given over the course of his or her life but for PD (not to be confused with the care that an individual with PD requires from others):

$${Val\_Life\_Care\_Lost}_{x}=$$

$$\begin{aligned} \frac{\frac{1}{r-g}(1-e^{(g-r)*1})}{l_{x}^{t}}\sum_{i=x}^{85} e^{(g-r)*(i-x)}{Hour\_Earn}_{i}^{EO,0}{AH\_care}_{i}^{t+(i-x)}L_{i}^{t+(i-x)}\#\left( A4.19 \right) \end{aligned}$$

In this formula, $\text{t}\text{ }\text{=}\text{ }\text{0 }$(i.e., year 2015). Note that given our assumption that age-specific hours of caregiving do not vary with time, the variable ${\text{AH\_care}\text{ }}_{\text{x}}^{\text{t}}$ can be replaced with ${\text{AH\_care}\text{ }}_{\text{x}}^{\text{0}}$ in Equation A4.19.

Equation A4.20 gives the value of lifetime volunteering lost due to PD:

$${Val\_Life\_Vol\_Lost}_{x}=$$

$$\begin{aligned} \frac{\frac{1}{r-g}(1-e^{(g-r)*1})}{l_{x}^{t}}\sum_{i=x}^{85} e^{(g-r)*(i-x)}{Hour\_Earn}_{i}^{EO,0}{AH\_vol}_{i}^{t+(i-x)}L_{i}^{t+(i-x)}\#\left( A4.20 \right) \end{aligned}$$

In this formula, $\text{t = 0 }$(i.e., year 2015). Note that given our assumption that age-specific hours of volunteering do not vary with time, the variable ${\text{AH\_vol}\text{ }}_{\text{x}}^{\text{t}}$ can be replaced with ${\text{AH\_vol}\text{ }}_{\text{x}}^{\text{0}}$ in Equation A4.20.

#### **A4.2.5. Formula for the indirect costs of death**

The indirect costs of death constitute the utilities that are attached to Markov states with the following labels in Figure 1: “Dead from ICAP,” “Dead from B,” and “Dead from M.” We calculate the indirect costs of a PD-related death by summing the values of expected lifetime lost earnings, expected lifetime lost volunteering, expected lifetime lost caregiving, and expected lifetime lost housework, as shown in Equation A4.21:

$${IC}_{x,D}^{m}={Val\_Life\_Earn\_Lost}_{x}+{Val\_Life\_Vol\_Lost}_{x}+{Val\_Life\_Care\_Lost}_{x}$$

$$\begin{aligned} +{Val\_Life\_HWork\_Lost}_{x}\#\left( A4.21 \right) \end{aligned}$$

### **A4.3. Indirect costs of meningitis-related persistent vegetative state, severe disability and moderate disability**

The Markov states associated with meningitis are death, temporary disability, moderate disability, severe disability, and persistent vegetative state (PVS). The indirect costs of death are derived in Section A4.2, and the indirect costs of temporary disability are derived in the next subsection. In this subsection, we derive the indirect costs of PVS, severe disability and moderate disability. These derivations are based on the following assumptions (see Section A4): (i) an individual stays in a PVS state for four years and then dies (see the introduction to this section);^[[76]](#footnote-76)^ (ii) the life expectancy of an individual with a severe disability involves disability for the rest of that individual’s expected lifetime under such disability;^[[77]](#footnote-77)^ (iii) moderate disability persists for two years; and (iv) patients with a moderate disability are fully-recovered after the two-year duration of the disability.

#### **A4.3.1. Indirect costs of a persistent vegetative state**

We assume that during the four-year period of disability, an individual in a PVS is completely unable to work, perform housework, volunteer, or provide care. Because such an individual dies after this four-year period, the loss of lifetime earnings, housework, volunteering, and caregiving is identical to that of a death and is represented by our prior formulas (see Equations A4.18, A4.19, A4.20, and A4.21). We further assume that during this four-year period, this individual requires long-term care. (While in principle such care belongs in an estimate of direct costs, we include it with indirect costs since most estimates of the direct costs of PD do not include long-term home-based medical care.) We follow a study by Balia and Brau (2014) on long-term home care for the elderly in Europe in allowing three inputs into long-term home care: unpaid informal care performed by friends and family members, nursing care, and paid domestic helper care.^^[[78]](#footnote-78)^^ We calibrate our model using the following European-wide monthly hour ($HPM$) estimates of each type of care from this study, extrapolated to all individuals aged 18-85:

**Table S20. Hours of care per month required**

**by individuals with long-term disability, by**

**type of care received**

| **Type of Care** | **Hours of care received per month** |
| --- | --- |
| Informal care (IC) | 37.9 |
| Nursing care (NC) | 2.7 |
| Paid domestic help (PDC) | 9.3 |
| Data source: Balia and Brau (2014) | |

Each of these inputs has a shadow value, which we define in Equations A4.22–A4.24:

$$\begin{aligned} SV\_IC={ER}_{15+}*{Hour\_Earn}_{15+}^{AO}\#\left( A4.22 \right) \end{aligned}$$

$$\begin{aligned} SV\_NC={ER}_{15+}*{Hour\_Earn}_{15+}^{PO}\#\left( A4.23 \right) \end{aligned}$$

$$\begin{aligned} SV\_PDC={ER}_{15+}*{Hour\_Earn}_{15+}^{EO}\#\left( A4.24 \right) \end{aligned}$$

Hourly earnings across all occupations for adults of all ages,${\text{Hour\_Earn}\text{ }}_{\text{15+}}^{\text{AO}}$ , is €3.10 in 2014.^[[79]](#footnote-79)^ Hourly earnings in the elementary occupations for adults of all ages, ${\text{Hour\_Earn}\text{ }}_{\text{15+}}^{\text{EO}}$ , is €2.01 in 2014 (see Section A4.1.2.i for the derivation).^[[80]](#footnote-80)^ Hourly earnings in the “professional occupations,” denoted by ${\text{Hour\_Earn}\text{ }}_{\text{15+}}^{\text{PO}}$, for adults of all ages is €6.64 in 2014.^^[[81]](#footnote-81)^^ We premultiply hourly earnings in the shadow values by the adult employment rate to reflect the fact that when an individual requires long-term home-based care, those who will provide that care will not necessarily be diverted from other productive activities. We calibrate the employment rate using the 2015 adult employment rate in Turkey of 0.46.^[[82]](#footnote-82)^ We assume earnings grow at the same rate as per capita GDP to reflect long-term productivity growth. Using these values, Equation A4.25 gives the cost of PVS care:

$${Cost\_PVS\_Care}_{x,PVS}=\frac{\frac{1}{r-g}(1-e^{(g-r)*1})}{l_{x}^{t}}\sum_{i=x}^{min(x+3,85)} e^{(g-r)*(i-x)}*\{SV\_IC*HPM\_IC$$

$$\begin{aligned} + SV\_NC*HPM\_NC+SV\_PDC*HPM\_PDC\}*12*L_{i}^{t+\left( i-x \right)}\#\left( A4.25 \right) \end{aligned}$$

In Equation A4.25, $\text{t}\text{ }$= 0. Note that the upper limit of the summation reflects that care is required for four years or until the individual’s 86^th^ birthday, whichever comes first.

The indirect costs of PVS are defined by Equation A4.26:

$${IC}_{x,PVS}^{M}={Val\_Life\_Earn\_Lost}_{x,PVS}+{Val\_Life\_Vol\_Lost}_{x,PVS}+{Val\_Life\_Care\_Lost}_{x,PVS}$$

$$\begin{aligned} +{Val\_Hwork\_Lost}_{x,PVS}+{Cost\_PVS\_Care}_{x,PVS}\#\left( A4.26 \right) \end{aligned}$$

#### **A4.3.2. Indirect costs of severe disability**

An individual with severe disability cannot work, perform housework, volunteer, or provide care for the remainder of his or her life, and requires permanent long-term care. Therefore, the loss of lifetime earnings, housework, volunteering, and caregiving during the remainder of the severely disabled individual’s lifetime is identical to that of a death as represented by our prior formulas (see Equations A4.18, A4.19, A4.20, and A4.21) except that we account for empirical evidence suggesting that the severely disabled population faces relatively higher mortality risks compared to those of the general population. We do so by adjusting the mortality rates for the general population upward using mortality rate ratios (MRRs) for pneumococcal meningitis patients relative to a control population from Roed et al. (2010).^[[83]](#footnote-83)^ These MRRs are provided in Table S21:

**Table S21. Mortality rate ratio of individuals with pneumococcal meningitis,**

**by age at infection and years of follow-up, relative to mortality**

**risks faced by similarly aged members of the general population**

| Age at infection | Years of follow-up | Mortality rate ratio |
| --- | --- | --- |
| 0-19 | 0–9 | 8.04 |
|  | 10–29 | 8.05 |
| 20-39 | 0–9 | 4.60 |
|  | 10–29 | 1.90 |
| 40-59 | 0–9 | 3.83 |
|  | 10–29 | 2.35 |
| 60–79 | 0–9 | 1.58 |
|  | 10–29 | 1.50 |
| 80–85 | 0–9 | 1.33 |
|  | 10–29 | 1.20 |

Data Source: Roed et al. (2010

For simplicity, to adjust mortality rates for the severely disabled population, we only model the age variation in MRRs. Thus, our methodology attempts to reflect the MRR age pattern in Table S21 by assuming the MRRs presented in Table S22, which are relative to the mortality risks faced by similarly aged members of the general population:

**Table S22. Estimated MRRs of**

**individuals with pneumococcal**

**meningitis**

| Age at infection | MRR |
| --- | --- |
| 18-19 | 4^[[84]](#footnote-84)^ |
| 20-29 | 4 |
| 30-39 | 4 |
| 40-49 | 3 |
| 50-59 | 3 |
| 60-69 | 2 |
| 70–79 | 2 |
| 80–85 | 1.5 |

We multiply the raw mortality rates that we obtain from the Eurostat Life Tables for 2014^[[85]](#footnote-85)^ by the age-appropriate MRR in Table S22. Using these adjusted mortality rates, we construct the life table variables described in Section A4.2. Having increased mortality rates by the MRRs in Table S22, the variable ${\text{Lsd}\text{ }}_{\text{x}}^{\text{t}}$, which is the number of person years lived between ages $\text{x}$ and age $\text{x}\text{ + 1}$ in year $\text{t}$ for a severely disabled individual, reflects the fact that individuals with severe disabilities face relatively higher mortality risks compared to individuals from the general population. Therefore, ${\text{Lsd}\text{ }}_{\text{x}}^{\text{t}}\text{ <}\text{ }\text{L}_{\text{x}}^{\text{t}}$.

Equation A4.27 provides the cost of permanent long-term care:

$${Cost\_Perm\_Care}_{x,SD}=\frac{\frac{1}{r-g}(1-e^{(g-r)*1})}{l_{x}^{t}}\sum_{i=x}^{85} e^{(g-r)*(i-x)}*\{SV\_IC*HPM\_IC+SV\_NC$$

$$\begin{aligned} *HPM\_NC+SV\_PDC*HPM\_PDC\}*12*{Lsd}_{i}^{t+\left( i-x \right)}\#\left( A4.27 \right) \end{aligned}$$

In this formula, $\text{t}\text{ }\text{=}\text{ }\text{0 }$(i.e., year 2015).

Equation A4.28 gives indirect costs for an individual with severe disability:

$${IC}_{x,SD}^{M}={Val\_Life\_Earn\_Lost}_{x,SD}+{Val\_Life\_Vol\_Lost}_{x,SD}+{Val\_Life\_Care\_Lost}_{x,SD}$$

$$\begin{aligned} +{Val\_Hwork\_Lost}_{x,SD}+{Cost\_Perm\_Care}_{x,SD}\#\left( A4.28 \right) \end{aligned}$$

#### **A4.3.3. Indirect costs of moderate disability**

Our model defines moderate disability ($MD$) as a state wherein an infected individual cannot work, perform housework, volunteer, or provide care for two years, but returns to full capacity to work, housework, volunteering, and providing care after that time.

##### **A4.3.3.i. Formulas for lifetime production lost**

The formulas that are used to calculate the indirect costs of moderate disability are provided in Equations A4.29-A4.32. These formulas are identical to their lifetime equivalents from Section A4.2, except the upper limit of the summation is *x* + 1 rather than 85:

$${Val\_Mod\_Earn\_Lost}_{x,MD}=$$

$$\begin{aligned} \frac{\frac{1}{r-g}(1-e^{(g-r)*1})}{l_{x}^{t}}\sum_{i=x}^{x+1} e^{(g-r)*(i-x)}{Ann\_Earn}_{i}^{0}{ER}_{i}^{t+(i-x)}L_{i}^{t+(i-x)}\#\left( A4.29 \right) \end{aligned}$$

$${Val\_Mod\_Hwork\_Lost}_{x,MD}=$$

$$\begin{aligned} \frac{\frac{1}{r-g}(1-e^{(g-r)*1})}{l_{x}^{t}}\sum_{i=x}^{x+1} e^{(g-r)*(i-x)}{Hour\_Earn}_{i}^{0}{AH\_Hwork}_{i}^{t+(i-x)}L_{i}^{t+(i-x)}\#\left( A4.30 \right) \end{aligned}$$

$${Val\_Mod\_Care\_Lost}_{x,MD}=$$

$$\begin{aligned} \frac{\frac{1}{r-g}(1-e^{(g-r)*1})}{l_{x}^{t}}\sum_{i=x}^{x+1} e^{(g-r)*(i-x)}{Hour\_Earn}_{i}^{0}{AH\_care}_{i}^{t+(i-x)}L_{i}^{t+(i-x)}\#\left( A4.31 \right) \end{aligned}$$

$${Val\_Mod\_Vol\_Lost}_{x,MD}=$$

$$\begin{aligned} \frac{\frac{1}{r-g}(1-e^{(g-r)*1})}{l_{x}^{t}}\sum_{i=x}^{x+1} e^{(g-r)*(i-x)}{Hour\_Earn}_{i}^{0}{AH\_vol}_{i}^{t+(i-x)}L_{i}^{t+(i-x)}\#\left( A4.32 \right) \end{aligned}$$

In these formulas, $\text{t}\text{ }\text{=}\text{ }\text{0 }$(i.e., year 2015). Note that given our assumption that age-specific employment rates and annual hours spent housekeeping, caregiving, and volunteering do not vary with time, the variables $\text{ER}_{\text{x}}^{\text{ }\text{t}}$, ${\text{AH\_Hwork}\text{ }}_{\text{i}}^{\text{t}}$ , ${\text{AH\_care}\text{ }}_{\text{i}}^{\text{t}}\text{,}$ and ${\text{AH\_vol}\text{ }}_{\text{i}}^{\text{t}}\text{ }$can be replaced with $\text{ER}_{\text{x}}^{\text{0}}$ , ${\text{AH\_Hwork}\text{ }}_{\text{i}}^{\text{0}}$ , ${\text{AH\_care}\text{ }}_{\text{i}}^{\text{0}}\text{,}$ and ${\text{AH\_vol}\text{ }}_{\text{i}}^{\text{0}}$ in Equations A4.29, A4.30, A4.31, and A4.32, respectively.

##### **A4.3.3.ii. Period of informal caregiving**

An individual who is in a state of moderate disability does not require any long-term care. We take the duration of informal caregiving from Wyrwich et al*.* (2015).^[[86]](#footnote-86)^ Equations A4.33.a and A4.33.b calculate the number of caregiver days that are required for individuals age $\text{x}$ who are temporarily disabled by infection manifestations.^^[[87]](#footnote-87)^^ Equation A4.33.a applies for $\text{m}$ equal to inpatient CAP, bacteremia, and meningitis, while Equation A4.33.b applies for $\text{m}$ equal to outpatient CAP.

$$\begin{aligned} {Care\_Days\_Required}_{x}^{m}=0.81*7.5*{ER}_{x}+0.76*16.9*\left( 1-{ER}_{x} \right) \#\left( A4.33.a \right) \end{aligned}$$

$$\begin{aligned} {Care\_Days\_Required}_{x}^{OCAP}=7*{ER}_{x}+11.4*\left( 1-{ER}_{x} \right) \#\left( A4.33.b \right) \end{aligned}$$

Based on the Wyrwich et al*.* (2015) study, the number 7.5 is the number of days of informal caregiving that infected individuals who are employed receive conditional on receiving informal caregiving at all, and 16.9 is the corresponding number for the unemployed. Based on this same study, the number 0.81 is the proportion of employed infected individuals receiving any informal care, and 0.76 is the corresponding proportion for the unemployed. For outpatient CAP (Equation A4.33.b), the numbers 7.0 and 11.4 are the number of caregiver days required for the employed and unemployed, respectively (2016 Jul 10 e-mail from R Saiko to JP Sevilla).

Because an informal caregiver would only need to miss work on weekdays to provide this care, Equation A4.34 reports how we measure the caregiver workdays lost:

$$\begin{aligned} {Caregiver\_Work\_Days\_Lost}_{x}^{m}=(\frac{5}{7})*{Care\_Days\_Required}_{x}^{m}\#\left( A4.34 \right) \end{aligned}$$

Each of these days has a shadow value, denoted by $\text{SV\_Caregiver\_Work\_Days\_Lost}$, which is equal to $\text{ER}_{\text{15+}}\text{*}\left( \text{1}/\text{2} \right)\text{*}{\text{D}\text{H}_{\text{work}}}_{\text{15+}}\text{*}{\text{Hour\_Earn}\text{ }}_{\text{15+}}^{\text{AO}}$.

##### **A4.3.3.iii. Formula for the indirect costs of moderate disability**

Combining all terms defined in this subsection, Equation A4.35 gives the indirect costs of moderate disability:

$${IC}_{x,MD}^{M}={Val\_Mod\_Earn\_Lost}_{x,MD}+{{Val\_Mod\_Hwork\_Lost}_{x,MD}+Val\_Mod\_Vol\_Lost}_{x,MD}+{Val\_Mod\_Care\_Lost}_{x,MD}$$

$$\begin{aligned} +{Caregiver\_Work\_Days\_Lost}_{x}^{m}*SV\_Caregiver\_Work\_Days\_Lost\#\left( A4.35 \right) \end{aligned}$$

Note that as with our expression for the indirect costs of death, our expression for the indirect costs of meningitis-related PVS, severe disability, and moderate disability depends on a continuous-time formula for discounted age-specific life expectancy. This dependency implies that our continuous-time models implicitly assume that these disabilities (like death) occur at the initial moment of the year in which it occurs, and that the lost productivity would have occurred in a continuous stream throughout that interval.

### **A4.4. Indirect costs of temporary disability**

Our analysis of temporary disability is structured in terms of three relevant durations within the treatment episode, which typically lasts less than a month (see Table S23 below). The first is the period of full incapacity, during which time the infected individual cannot perform any productive activities. The second is the period of partial capacity, during which time the infected individual is capable of performing some productive activities, but with impaired abilities to do so. The third is the period during which the infected individual requires an informal caregiver.

#### **A4.4.1. Period of full incapacity**

The period of full incapacity, which we denote $\text{Full\_Days\_Lost}^{\text{ }\text{m}}$, where $\text{m}$ represents the PD manifestation, is the number of days, including weekdays and weekends, during which a temporarily disabled individual is completely unable to do paid work, do housework, volunteer, or provide care when he or she would otherwise have done so. To account for the possibility that $\text{Full\_Days\_Lost}^{\text{ }\text{m}}\text{ }$varies by employment status, we distinguish between $\text{Full\_Days\_Lost}^{\text{ }\text{m,e}}$ and $\text{Full\_Days\_Lost}^{\text{ }\text{m,u}}$, where the superscript $\text{e}$ refers to “employed” and $\text{u}$ refers to “unemployed.”

$\text{Full\_Days\_Lost}^{\text{ }\text{m}}$ for bacteremia and meningitis are based on Jiang et al. (2012), who provide the number of full workdays (by assumption, weekdays) lost to IPD equal to 16.8.^^[[88]](#footnote-88)^^ The number 16.8 refers to the number of workdays lost in patients with IPD. Because we require the number of full days lost, inclusive of weekdays and weekends, during which an individual is unable either to work or do non-market labor, we estimate this number of full days lost by scaling up 16.8 by 7/5, which gives us 16.8 * 7 / 5 = 23.52.

$\text{Full\_Days\_Lost}^{\text{ }\text{m}}$ for inpatient and outpatient CAP are based on unpublished calculations performed by Kathleen Wyrwich and communicated to us by Reiko Sato. These calculations, which we report in Table S23 below, disaggregate the results in Table 3 of a study by Wyrwich et al. (2015). The numbers 15.6 and 7.0 represent the number of days of usual activities or work missed among employed individuals aged 50 and older treated for inpatient and outpatient CAP, respectively, in the United States. The numbers 18.3 and 11.4 are the corresponding numbers for unemployed individuals. Because the number of days in Wyrwich et al. (2015) appear to include weekends (i.e., the number of days of *usual* *activities or work* missed), we use these values in our measurements of lost time spent volunteering, caregiving, and doing housework. Table S23 reports these data:

| **Table S23. Number of full days of productivity lost by manifestation and employment status** | | |
| --- | --- | --- |
| **Manifestation** | **Employment status** | **Full days lost** |
| Inpatient CAP | Employed | 15.60 |
|  | Unemployed | 18.30 |
| Outpatient CAP | Employed | 7.00 |
|  | Unemployed | 11.40 |
| Bacteremia | Employed | 23.52 |
|  | Unemployed | 23.52 |
| Meningitis | Employed | 23.52 |
|  | Unemployed | 23.52 |

Data source: Wyrwich et al. (2015)

Equation A4.36 gives the average $\text{Full\_Days\_Lost}^{\text{ }\text{m}}$ across employed and unemployed individuals.

$$\begin{aligned} {Full\_Days\_Lost}^{m}={ER}_{x}*{Full\_Days\_Lost}^{m,e} +\left( 1-{ER}_{x} \right)*{Full\_Days\_Lost}^{m,u}\#\left( A4.36 \right) \end{aligned}$$

Here, $\text{ER}_{\text{x}}$ refers to the employment rate (see Section A4.1.1) and $\text{m}$ denotes the relevant PD manifestation. Because $\text{Full\_Days\_Lost}^{\text{ }\text{m,e}}$ includes both weekdays and weekends, and because we assume that employed individuals work only on weekdays, we define $\text{Full\_Workdays\_Lost}^{\text{ }\text{m}}$ to be equal to 5/7 the full days lost among the employed, as Equation A4.37 shows.

$$\begin{aligned} {Full\_Workdays\_Lost}^{m,e}=(\frac{5}{7}){Full\_Days\_Lost}^{m,e}\#\left( A4.37 \right) \end{aligned}$$

#### **A4.4.2. Indirect costs during the period of full incapacity**

To calculate the indirect costs during the period of full incapacity, we define the following variables: $\text{Full\_Days\_Lost}^{\text{ }\text{m}}$, which is equal to the number of days of housework, caregiving, and volunteering fully lost to temporary disability (see Equation A4.36); and $\text{Full\_Workdays\_Lost}^{\text{ }\text{m}}$, which is equal to the number of work days that are fully lost due to temporary disability (see Equation A4.37). We denote daily hours of work, housework, caregiving, and volunteering by $\text{DH\_work}_{\text{15+}}$, $\text{DH\_hwork}_{\text{x}}$, $\text{DH\_care}_{\text{x}}$, $\text{DH\_vol}_{\text{x}}$, respectively, where the subscript $15+1$ denotes the average value for ages 15 and older, and the subscript $x$ denotes the average value for individuals of age $\text{x}$. The data sources and methodology that we use to construct the non-market time-use variables (housework, caregiving, and volunteering) are provided in Section A4.1.3.ii. The estimated daily hours of market-based production $\text{DH\_work}_{\text{15+}}$ is 9.48 (see Section A4.1.3.i). We denote the hourly wage across all occupations, which is equal to €3.10, by $\text{Hour\_Earn}_{\text{ }\text{x}}^{\text{ }\text{AO}}$. The hourly earnings in the elementary occupations, which is equal to €2.01, is denoted by $\text{Hour\_Earn}_{\text{ }\text{x}}^{\text{ }\text{EO}}$. Section A4.1.2.i provides the data and methodology used to create these hourly earnings variables.

Using the data inputs described directly above, Equation A4.38 gives the indirect costs during the period of full incapacity, where the time elements are in the curly brackets:

$$\{{Full\_Workdays\_Lost}^{m,e}*{ER}_{x}*{DH\_work}_{15+}\}*{Hour\_Earn}_{x}^{AO}+\{{Full\_Days\_Lost}^{m}$$

$$\begin{aligned} *({DH\_hwork}_{x}+{DH\_care}_{x}+{DH\_vol}_{x})\}*{Hour\_Earn}_{x}^{EO}\#\left( A4.38 \right) \end{aligned}$$

#### **A4.4.3. Period of partial capacity**

The period of partial capacity, which we denote $\text{Part\_Days\_Lost}^{\text{ }\text{m}}$, represents the number of days during which a disabled individual is only partially able to work, volunteer, give care, or do housework when he or she would otherwise have done so as much as a healthy individual would. We do not have direct measures of this duration. We therefore infer it from the difference between the number of days an individual takes to recover fully from the temporary disability (denoted by $\text{Days\_Full\_Recov}^{\text{ }\text{m}}$) and the number of days during which an individual is fully incapacitated (our variable $\text{Full\_Days\_Lost}^{\text{ }\text{m}}$). Given data imperfections stemming from multiple sources for these parameters, this calculated difference may be negative, in which case we set its value to zero. Our data source reports $\text{Days\_Full\_Recov}^{\text{ }\text{m}}$ that vary by manifestation $m$ and employment status (2016 Jul 10 e-mail from R Saiko to JP Sevilla). The number 18.9 is the number of days until usual performance level is reached at work or in other usual daily activities among employed individuals aged 50 and older who were hospitalized for CAP in the United States, and 28.4 is the corresponding number for unemployed individuals. Although these numbers derive from a study of CAP,^[[89]](#footnote-89)^ we extrapolate them to bacteremia and meningitis for lack of better alternatives. The corresponding values for outpatient CAP are 12.4 and 21.4. Table S24 reports these data:

| **Table S24. Number of days to full recovery by manifestation and employment status** | | |
| --- | --- | --- |
| **Manifestation** | **Employment status** | **Days to full recovery** |
| Inpatient CAP | Employed | 18.9 |
|  | Unemployed | 28.4 |
| Outpatient CAP | Employed | 12.4 |
|  | Unemployed | 21.4 |
| Bacteremia | Employed | 18.9 |
|  | Unemployed | 28.4 |
| Meningitis | Employed | 18.9 |
|  | Unemployed | 28.4 |

Data source: 2016 July 10, 2016 e-mail from R Saiko to JP Sevilla based on a direct correspondence with K Wyrwich, the lead author of: Wyrwich et al. (2015).

Using the number of days to full recovery and the number of days during which an individual is fully incapacitated, we define days of partial capacity such that it varies with employment status, as Equations A4.39 and A4.40 show.

$$\begin{aligned} {Part\_Days\_Lost}^{m,e}=max({Days\_Full\_Recov}^{m,e}-{Full\_Days\_Lost}^{m,e},0)\#\left( A4.39 \right) \end{aligned}$$

$$\begin{aligned} {Part\_Days\_Lost}^{m,u}=max({Days\_Full\_Recov}^{m,u}-{Full\_Days\_Lost}^{m,u},0)\#\left( A4.40 \right) \end{aligned}$$

Equation A4.41 gives the average days of partial capacity across the employed and unemployed.

$$\begin{aligned} {Part\_Days\_Lost}^{m}={ER}_{x}*{Part\_Days\_Lost}^{m,e}+(1-{ER}_{x})*{Part\_Days\_Lost}^{m,u}\#\left( A4.41 \right) \end{aligned}$$

Under the assumption that an employed individual works five days per week, Equation A4.42 reports how we measure the workdays at partial capacity.

$$\begin{aligned} {Part\_Workdays\_Lost}^{m,e}=(\frac{5}{7}){Part\_Days\_Lost}^{m,e}\#\left( A4.42 \right) \end{aligned}$$

We further assume that an individual will not return to work—whether paid or unpaid—unless he or she has at least half the normal productivity, and that productivity rises linearly with time until full recovery. Thus, the average productivity during the period of partial capacity is [(1/2 + 1) / 2] = 3/4 of full productivity. The productivity loss from partial capacity is therefore 1/4.

#### **A4.4.4. Indirect costs during the period of partial capacity**

Equation A4.43 gives the indirect costs during the period of partial capacity:

$$\{{Part\_Workdays\_Lost}^{m,e}*{ER}_{x}*{DH\_work}_{15+}\}*[\left( \frac{1}{4} \right){Hour\_Earn}_{x}^{AO}]+\{{Part\_Days\_Lost}^{m}$$

$$\begin{aligned} *\left( {DH\_hwork}_{x}+{DH\_care}_{x}+{DH\_vol}_{x} \right)\}*[\left( \frac{1}{4} \right){Hour\_Earn}_{x}^{EO}]\#\left( A4.43 \right) \end{aligned}$$

#### **A4.4.5. Value of informal caregiving**

We assume that anyone who must provide informal care to a disabled individual during the workweek takes a half-day off from work each day care is provided. Based on this assumption, the informal caregiver gives up a number of hours equal to $\left( \text{1}/\text{2} \right)\text{*}\text{DH\_work}_{\text{15+}}$ for each caregiver workday lost, where $\text{DH\_work}_{\text{15+}}$ is daily hours worked among the employed of all ages. We also assume that only the employed must take time off work, so that the value of caregiver workdays lost depends on the employment rate. However, because we do not necessarily know the age of the caregiver, we use both the daily hours worked and the employment rate for the entire adult population (effectively defined by Eurostat as beginning at age 15), which we denote by $\text{DH\_work}_{\text{15+}}$ and $\text{ER}_{\text{15+}}$, respectively. Finally, we use the hourly earnings across all occupations for the entire adult population, denoted by $\text{Hour\_Earn}_{\text{15+}}^{\text{ A}\text{O}}$ , to value each hour of foregone work.

As reported earlier, daily hours worked among the employed of all ages is $\text{DH\_work}_{\text{15+}}\text{=}\text{ }\text{9.48}$. The employment rate for adults of all ages is $\text{ER}_{\text{15+}}\text{=}\text{ }\text{0.46}$, and the hourly earnings across all occupations for adults of all ages is $\text{Hour\_Earn}_{\text{15+}}^{\text{ A}\text{O}}\text{=}\text{ }\text{€}\text{3.}\text{10}$. Section A4.1.1 and Section A4.1.2 provides the data and methodology for calculating these employment rate and hourly earnings values, respectively. Combining these values with the number of caregiver workdays lost, denoted by$\text{ }\text{Caregiver\_Work\_Days\_Lost}_{\text{x}}^{\text{ }\text{m}}$ (see Equation A4.34), creates the value of caregiver workdays lost that is expressed in Equation A4.44, where time is reported in curly brackets, and the shadow value is the hourly earnings:

$${Val\_Caregiver\_Work\_Days\_Lost}_{x}^{m}=\{{Caregiver\_Work\_Days\_Lost}_{x}^{m}*{ER}_{15+}*(\frac{1}{2})*{DH\_work}_{15+}\}$$

$$\begin{aligned} *{Hour\_Earn}_{15+}^{AO}\#\left( A4.44 \right) \end{aligned}$$

#### **A4.4.6. Formula for the indirect costs of temporary disability**

The indirect costs of temporary disability are calculated as the sum of the indirect costs that are associated with the days of full incapacity, the days of partial capacity, and the number of caregiver workdays lost:

$${IC}_{x,TD}^{m}=$$

$$\{{Full\_Workdays\_Lost}^{m,e}*{ER}_{x}*{DH\_work}_{15+}\}*{Hour\_Earn}_{x}^{AO}+\{{Full\_Days\_Lost}^{m}*({DH\_hwork}_{x}+{DH\_care}_{x}+{DH\_vol}_{x})\}*{Hour\_Earn}_{x}^{EO}+$$

$$\{{Part\_Workdays\_Lost}^{m,e}*{ER}_{x}*{DH\_work}_{15+}\}*[\left( \frac{1}{4} \right){Hour\_Earn}_{x}^{AO}]+\{{Part\_Days\_Lost}^{m}*({DH\_hwork}_{x}+{DH\_care}_{x}+{DH\_vol}_{x})\}*[\left( \frac{1}{4} \right){Hour\_Earn}_{x}^{EO}]+$$

$$\begin{aligned} \{{Caregiver\_Work\_Days\_Lost}_{x}^{m}*{ER}_{15+}*(\frac{1}{2})*{DH\_work}_{15+}\}*{Hour\_Earn}_{15+}^{AO}\#\left( A4.45 \right) \end{aligned}$$

#### **A4.4.7. Indirect costs of temporary disability for the comorbid subpopulation**

To calculate the indirect costs of temporary disability for the comorbid subpopulation, we adjust the parameters that enter our temporary disability formulas for comorbid individuals by scaling up those parameters that are related to the length of hospital stay (LOS) and the duration of temporary disability.^[[90]](#footnote-90)^ These parameters, which we derive above, include: (1) the number of full days of non-market productivity lost ($Full\_Days\_Lost$); (2) the number of full work days lost ($\text{Full\_Workdays\_Lost}$); (3) the number of days that it takes to fully recover from a temporary disability ($Days\_Full\_Recov$); and (4) the number of caregiver work days lost ($Care\_Days\_Required$).

We scale up the parameters listed above using data from the United States to construct the ratio of the weighted average of LOS across PD risk groups in the comorbid subpopulation to that in the general population. For this purpose, we use age-invariant U.S. risk-group-disaggregated values of LOS for CAP and for IPD from Weycker et al. (2016)^^[[91]](#footnote-91)^^ as expressed in Equation A4.46:

$$\begin{aligned} {SF}_{LOS}^{x,m}=\frac{{LOS}_{low}^{m}*{pc}_{low}^{x}+{LOS}_{medium}^{m}*{pc}_{medium}^{x}+{LOS}_{high}^{m}*{pc}_{high}^{x}}{{LOS}_{low}^{m}*p_{low}^{x}+{LOS}_{medium}^{m}*p_{medium}^{x}*{LOS}_{high}^{m}*p_{high}^{x}}\#(A4.46) \end{aligned}$$

In Equation A4.46, the superscript $m$ denotes the PD outcomes CAP and IPD, $p_{low}$, $p_{medium}$ and $p_{high}$ are the PD risk-group specific prevalence rates in the general population, and ${pc}_{low}$, ${pc}_{medium}$ and ${pc}_{high}$ are the corresponding prevalence rates in the comorbid subpopulation. These prevalence rates are presented in Table S4 and S5, respectively.

Note that since the Weycker et al. (2016) LOS values are age-invariant, the LOS scale factor in Equation A4.46 varies with age only due to age variation in the prevalence weights. We collapse these values into a single scale factor by taking a weighted average of the age-varying values, where the weights are proportional to age-specific population size. We use age-specific population sizes from the World Population Prospects 2015,^[[92]](#footnote-92)^ which we then scale down to reflect the portion that represents the comorbid subpopulation. We do so by multiplying raw population values by the age-specific prevalence of having one or more policy-relevant comorbidity. These prevalence rates are presented in Table 1 of the article.

Upon examining the variation in IPD LOS across risk groups, the Weycker et al. (2016) data did not reveal any discernable pattern.^^[[93]](#footnote-93)^^ We therefore assume that IPD-related LOS is risk-group invariant and we set the IPD LOS scale factor equal to 1.

# A5. Vaccination benefits and rates of return

The utility of each PD-related Markov state consists of the indirect costs associated with that state. Vaccination produces benefits by reducing risks of contracting PD (see node B in Figure 1) and therefore risks of transitioning to states with positive state utilities. The benefit of vaccination is the difference in expected discounted lifetime costs (or expected discounted cumulative state utilities) between vaccinated and unvaccinated cohorts.

## **A5.1. Markov cycle utilities**

We associate each cycle with a cycle utility that is equal to the expected value of state utilities within that cycle.

$$\begin{aligned} {CU}_{x}^{t}=\left( 1-q_{x}^{t0,nPD}/2 \right)*{EYC}_{x}^{t} \#\left( A5.1 \right) \end{aligned}$$

where:

$$\begin{aligned} {EYC}_{x}^{t}=\left( i_{x}^{t,ICAP}C_{x}^{ICAP}+i_{x}^{t,OCAP}C_{x}^{OCAP}+i_{x}^{t,B}C_{x}^{B}+i_{x}^{t,M}C_{x}^{M} \right)\#\left( A5.2 \right) \end{aligned}$$

$$\begin{aligned} C_{x}^{ICAP}=p_{x,D}^{ICAP}{*IC}_{x,D}^{ICAP}+p_{x,TD}^{ICAP}*{IC}_{x,TD}^{ICAP}\#\left( A5.3 \right) \end{aligned}$$

$$\begin{aligned} C_{x}^{OCAP}={IC}_{x,TD}^{OCAP}\#\left( A5.4 \right) \end{aligned}$$

$$\begin{aligned} C_{x}^{B}=p_{x,D}^{B}*{IC}_{x,D}^{B}+p_{x,TD}^{B}*{IC}_{x,TD}^{B}\#\left( A5.5 \right) \end{aligned}$$

$$\begin{aligned} C_{x}^{M}=p_{x,D}^{M}*{IC}_{x,D}^{M}+p_{x,PVS}^{M}*{IC}_{x,PVS}^{M}+p_{x,SD}^{M}*IC_{x,SD}^{M}+p_{x,MD}^{M}*{IC}_{x,MD}^{M}+p_{x,TD}^{M}*{IC}_{x,TD}^{M}\#\left( A5.6 \right) \end{aligned}$$

In the above expressions, $i_{x}^{t,ICAP}$, $i_{x}^{t,OCAP}$, $i_{x}^{t,B}$, and $i_{x}^{t,M}$ are the vaccine-type incidence rates associated with inpatient CAP, outpatient CAP, bacteremia, and meningitis, respectively, faced by an individual aged $x$ in year $t$; and $\text{C}_{\text{x}}^{\text{ }\text{ICAP}}\text{,}\text{ }\text{C}_{\text{x}}^{\text{ }\text{OCAP}}\text{,}\text{ }\text{C}_{\text{x}}^{\text{ }\text{B}}\text{,}\text{ }\text{C}_{\text{x}}^{\text{ }\text{M}}$ are the corresponding manifestation-specific expected cost of infection, where the expectations are taken with respect to the possible outcomes of infection. For example, since an inpatient CAP infection can result in either death or temporary disability, the expected cost of an inpatient CAP infection $\text{C}_{\text{x}}^{\text{ }\text{ICAP}}$ equals the sum of the indirect costs of death following inpatient CAP, denoted by ${IC}_{x,D}^{ICAP}$, times the probability of death from an inpatient CAP infection, denoted by $p_{x,D}^{ICAP}$, and the indirect costs of temporary disability from inpatient CAP, denoted by ${IC}_{x,TD}^{ICAP}$, times the probability of temporary disability from an inpatient CAP infection, denoted by $p_{x,TD}^{ICAP}$. These manifestation-specific expected costs vary with both $\text{x}$ and $\text{t}$, though our notation does not explicitly index by $\text{t.}$

$\text{EYC}_{\text{x}}^{\text{ }\text{t}}$ is the expected cost of infection across all manifestations and equals the sum across manifestations of the probability of infection with that manifestation (represented by its incidence rate) times the expected cost of an infection with that manifestation.

The cycle utility $\text{CU}_{\text{x}}^{\text{ }\text{t}}$ multiplies $\text{EYC}_{\text{x}}^{\text{ }\text{t}}$ by the probability of not dying from non-PD-related causes, which is equal to $\text{1-}\text{ }\text{q}_{\text{x}}^{\text{ }\text{t0,nPD}}\text{/2}$ (see Equation A5.1).

## **A5.2. Cumulative utility**

A cohort entering the Markov model faces a “cumulative utility” equal to the expected present discounted value of the sum of cycle utilities over that cohort’s lifetime, where the expectation is taken with respect to the likelihood of surviving to a particular cycle. In this subsection, we derive the cumulative utility for the vaccinated and non-vaccinated cohorts.

### **A5.2.1. Non-vaccinated cohort**

To derive an expression for cumulative utility, we again depart from the discrete time framework of the Markov model and adopt a continuous time model. We model each cycle as a year-long interval in continuous time and treat expected cycle costs as accruing at the first moment of that interval. We then discount from the initial moment of some future cycle back to the initial moment of the initial cycle with continuous-time discounting between the moments. This formulation yields a cumulative utility expressed in Equation A5.7:

$$\begin{aligned} {EC}_{x0}=\frac{1}{r-g}\left( 1-e^{(g-r)*1} \right)\sum_{x=x0}^{85} e^{(g-r)*t}{CU}_{x}^{t}l_{x}^{t}\#\left( A5.7 \right) \end{aligned}$$

where $\text{CU}_{\text{x}}^{\text{ }\text{t}}$ is cycle utility and $\text{l}_{\text{x}}^{\text{ }\text{t}}$ is the survival function.

### **A5.2.2. Vaccinated cohort**

For a vaccinated cohort, we use the same formulas as for the unvaccinated cohort, except that we use post-vaccination incidence rates instead of pre-vaccination rates. The relevant altered formulas are:

$$\begin{aligned} {Mv}_{x}^{t}=M_{x}^{t0,nPD}+\left( {iv}_{x}^{t,ICAP}{cfr}_{x}^{ICAP}+ {iv}_{x}^{t,B}{cfr}_{x}^{B}+ {iv}_{x}^{t,M}{cfr}_{x}^{M} \right)\#\left( A5.8 \right) \end{aligned}$$

$$\begin{aligned} {qv}_{x}=\frac{{Mv}_{x}^{t}}{1+\frac{{Mv}_{x}^{t}}{2}}\#\left( A5.9 \right) \end{aligned}$$

$$\begin{aligned} {lv}_{50}=1\#\left( A5.10 \right) \end{aligned}$$

$$\begin{aligned} {lv}_{x}={lv}_{x-1}*\left( 1-{qv}_{x-1} \right), 50<x\leq85\#\left( A5.11 \right) \end{aligned}$$

$$\begin{aligned} {EYCv}_{x}^{t}=\left( {iv}_{x}^{t,ICAP}C_{x}^{ICAP}+{iv}_{x}^{t,OCAP}C_{x}^{OCAP}+{iv}_{x}^{t,B}C_{x}^{B}+{iv}_{x}^{t,M}C_{x}^{M} \right)\#\left( A5.12 \right) \end{aligned}$$

$$\begin{aligned} {CUv}_{x}^{t}=\left( 1-q_{x}^{t0,nPD}/2 \right)*{EYCv}_{x}^{t} \#\left( A5.13 \right) \end{aligned}$$

$$\begin{aligned} {ECv}_{x0}=\frac{1}{r-g}\left( 1-e^{(g-r)*1} \right)\sum_{x=x0}^{85} e^{(g-r)*t}{CUv}_{x}^{t}{lv}_{x}^{t}\#\left( A4.14 \right) \end{aligned}$$

## **A5.3. Formula for rates of return**

Given these expressions for cumulative utility with and without vaccination, Equation A5.15 gives the benefit to an individual of age $x$ of getting vaccinated at time $\text{t}\text{ }\text{=}\text{ }\text{0}$:

$$\begin{aligned} B_{x0}={EC}_{x0}-{ECv}_{x0}>0\#\left( A5.15 \right) \end{aligned}$$

We compute the average benefit across all ages in the entire Turkish PCV13 Adult program as a population-weighted average of benefits using weights that are proportional to population size. To construct these weights, we use population values come from the World Population Prospects 2015.^^[[94]](#footnote-94)^^ We construct weights for ages in the comorbid subpopulation by first scaling down these population values to reflect the portion that represents the comorbid population. That is, for an individual age $\text{x}$ in the comorbid subpopulation, we estimate the population as:

$$\begin{aligned} {pop}_{x}={pop}_{x}^{data}*{prevalence}_{x}\#(A5.16) \end{aligned}$$

where ${pop}_{x}^{data}$ is the raw population value from the World Population Prospects 2015 for age $\text{x}$, and ${prevalence}_{x}$ is the age-specific prevalence of one or more policy-relevant comorbidity that are derived in Section A3.1.4 and presented in Table 1 of the article. The resulting set of population weights sum to one across the entire cohort aged 18-85 year.

With population values thus defined, we compute the average benefit across all ages 18-85 years in the Turkish PCV13 Adult program using Equation A5.17:

$$\begin{aligned} B_{\bar{x}0}=\frac{\sum_{x0} {pop}_{x0}*B_{x0}}{\sum_{x0} {pop}_{x0}}\#\left( A5.17 \right) \end{aligned}$$

Here, $\text{pop}_{\text{x0}}$ is equal to the number of individuals who are age $\text{x}$ in time $\text{t}\text{ }\text{=}\text{ }\text{0}$ using the methods described above. Given this population-age-weighted average benefit, Equation A5.18 gives our rate of return (RoR) to the entire Turkish PCV13 Adult program:

$$\begin{aligned} r=\frac{B_{\bar{x}}}{c_{v}}-1\#\left( A5.18 \right) \end{aligned}$$

where $\text{c}_{\text{v}}$ is the cost of vaccination. Since vaccination costs in Turkey are not in the public domain, we present hypothetical RoRs for a range of vaccination costs between $10 and $150.

# A6. Other topics

In this subsection, we describe our sensitivity analysis, our method for summarizing indirect costs, and our method for converting indirect costs and benefits from the local currency to 2017 U.S. dollars.

## **A6.1. Sensitivity analyses**

Our first scenario analysis replaces our baseline vaccine efficacies with age-invariant baseline vaccine efficacies. We discuss this scenario analysis in Section A3.2.4. In this subsection, we describe our analyses that assesses the sensitivity of vaccine benefits to the magnitude of herd effects from PCV13 Pediatric and vaccine coverage rates.

Our second sensitivity analysis adjusts the magnitude of herd effects in our model to varying the levels of herd effects. As we discuss in Section A3.2.2, in our base case analysis with herd effects, the post-herd-effects steady state equals ${IRR}_{total}^{PCV13}=0.12$ times the value of the pre-herd-effects incidence rate. As the magnitude of the herd effects goes to zero, the value of ${IRR}_{total}^{PCV13}$ goes to 1.00. In this sensitivity analysis, we run our model for intermediate values of ${IRR}_{total}^{PCV13}$ between 0.12 and 1.00 at intervals of 0.04—that is, for values of 0.12, 0.16, 0.20, …, 0.92, 0.96, and 1.00. To implement this sensitivity analysis, an adjustment must be made to our base case vaccine-type incidence rates that are derived in Section A3.2 and presented in Table 1 of the article. As we also discuss in Section A3.2.2, in our base case analysis we assume that half of the herd effect period, and therefore the herd effect itself, has already materialized. Thus, to implement our base case analysis with herd effects, we multiply our baseline vaccine-type incidence rates presented in Table S6 by $\sqrt{0.12}$ to capture the herd effects that happened *before* 2015. In our sensitivity analysis that examines counterfactual magnitudes of herd effects, we must undo any actual pre-2015 herd effects reflected in our incidence rates before we add on the counterfactual pre- and post-2015 herd effects. We do so by dividing the vaccine-type incidence rates in Table 1 of the article by $\sqrt{0.12}$.

In our base-case analysis, we use the PCV13 vaccine coverage rate of 68% found in Oksuz and Gurler (2017). We perform a third sensitivity analysis wherein we use alternative values of 85.5% and 56.6% based on Ceyhan et al*.* (2016) estimates of PCV13 coverage of invasive isolates for Turkey and Hungary, respectively.^[[95]](#footnote-95)^ This study provides country-specific estimates of PCV13 coverage for the following Central and Eastern Europe countries: Croatia, Czech Republic, Hungary, Poland, Romania, Russia, Slovakia, and Turkey. We select the Hungary estimates for our lower bound because it is the lowest value relative to the other country-specific estimates that do not include non-typed serotypes.

## **A6.2. Averaging indirect costs across ages**

In Table 2 and Table 3 of the article, we present the average indirect costs per treatment episode and the average indirect costs per treatment episode by PD manifestation and disease outcome, respectively. In both tables, we present these averages separately for the comorbid and elderly subpopulations. In this section, we present our methodology for calculating the averages that we report in these tables.

Denoting the average indirect costs resulting from infection with manifestation $m$ by $\text{IC}_{\bar{\text{x}}}^{\text{ }\text{m}}$, for the elderly subpopulation aged 65-85 years, each age is weighted by the product of the age- and manifestation-specific incidence rate $i_{x}^{m}$ and that age’s population $\text{pop}_{\text{x}}$ from the World Population Prospects 2015.^^[[96]](#footnote-96)^^ The population data are available in five-year age groups from ages 0–89 years. We assume that the populations are uniform across the ages within an age group; thus, we divided the five-year population values evenly among the ages within a group. Given some age-specific indirect costs per treatment episode $\text{IC}_{\text{x}}^{\text{ }\text{m}}$, we compute the average indirect costs associated with manifestation $m$ across all ages using Equation A6.1:

$$\begin{aligned} {IC}_{\bar{x}}^{m}=\frac{\sum_{x} i_{x}^{m}{*pop}_{x}* {IC}_{x}^{m}}{\sum_{x} i_{x}^{m}{*pop}_{x}}\#\left( A6.1 \right) \end{aligned}$$

To calculate the average indirect costs across ages within the comorbid subpopulation, we use the same formula except that we scale down the raw population values to reflect the portion that represents the comorbid population. That is, for the comorbid subpopulation, $\text{pop}_{\text{x}}$ in Equation A6.1 is equal to ${pop}_{x}^{data}*{prevalence}_{x}$ as defined in Section A5.3 and by Equation A5.15.

## **A6.3. Expected indirect costs**

We estimate the expected indirect costs of inpatient CAP, meningitis and bacteremia as equal to the manifestation- age-specific indirect costs $IC_{x}^{m}$ multiplied by our base year 2015 manifestation- age-specific vaccine-type incidence rate for the unvaccinated $i_{x}^{2015,m}$ summed across all ages from 18-85:

$$\begin{aligned} E{IC}_{\bar{x}}^{m}=\sum_{x} i_{x}^{2015,m}IC_{x}^{m} \#\left( A6.2 \right) \end{aligned}$$

## **A6.4. Currency conversion**

Our average indirect costs and vaccination benefit calculations are denominated in 2017 U.S. dollars. To implement this conversion, our first step is to convert 2014 euros to 2017 euros. We do this by multiplying 2014 euro values by the ratio of Turkey’s 2017 GDP deflator to its 2014 value, which equals 1.29.^^[[97]](#footnote-97)^^ The second step is to convert 2017 euros to 2017 U.S. dollars. We do this using the average monthly exchange rate from January to December 2017 from the U.S. Federal Reserve Economic Data, which equals 1.13.^^[[98]](#footnote-98)^^ The final conversion factor by which we multiply 2014 euro values to arrive at 2017 U.S. dollar values is 1.29 * 1.13 = 1.46.

# References

Akin L, Kaya M, Altinel S, Durand L. Cost of pneumococcal infections and cost-effectiveness analysis of pneumococcal vaccination at risk adults and elderly in Turkey. Hum Vaccin. 2011;7(4):441–450. doi:10.4161/hv.7.4.14188

Akyil FT, Yalcinsoy MA, Hazar A, Cilli B, Celenk O, Kilic A, Sayiner N, Kokturk A, Sakar CA, Filiz E, Cakir E. Prognosis of hospitalized patients with community-acquired pneumonia. Pulmonology. 2018;24(3):164–169. ISSN 2531-0437. https://doi.org/10.1016/j.rppnen.2017.07.010.

American Diabetes Association. Economic costs of diabetes in the US in 2012. Diabetes Care. 2013;36(6):1033–1046.

Ateş K. Türkiye’de diyabet ve kronik böbrek hastalığı: CREDIT çalışması. Presentation by Türk Nefrologı Derneğı; see slides 18, 21, 48. <http://www.nefroloji.org.tr/folders/file/hekimlik/salon2/Kenan_Ates.pdf>.

Balia S, Brau R. A country for old men? Long-term home care utilization in Europe. Health Econ. 2014;23(10):1185–1212.

Bonten M, Huijts SM, Bolkenbaas M, Webber C, Patterson S, Gault S, van Werkhoven CH, van Deursen AMM, Sanders EAM, Verheij TJM, et al. Polysaccharide conjugate vaccine against pneumococcal pneumonia in adults. N Engl J Med. 2015;372(12):1114–1125.

Centers for Disease Control and Prevention, Vaccine Information for Adults. Lung disease including asthma and adult vaccination. Washington (DC): U.S. Department of Health & Human Services; 2016 May 2 [accessed on 2019 Sep 29]. <https://www.cdc.gov/vaccines/adults/rec-vac/health-conditions/lung-disease.html>.

Ceyhan M, Dagan R, Sayiner A, Chernyshova L, Dinleyici EC, Hryniewicz W, Kulcsar A, Mad’arova L, Pazdiora P, Sidorenko S, et al. Surveillance of pneumococcal diseases in Central and Eastern Europe. Hum Vaccin Immunother. 2016;12(8):2121-2134. <http://dx.doi.org/10.1080/21645515.2016.1159363>.

Değertekin M, Erol C, Ergene O, Tokgözoğlu L, Aksoy M, Erol MK, Eren M, Şahin M, Eroğlu E, et al. Türkite’deki kalp yetersizliği prevalansı ve öngördürücüleri: HAPPY çalışması. Türk Kardiyol Dern Arş. 2012;40(4):298–308.

DeLong JB. The theory of economic growth. In: DeLong JB, editor. Macroeconomics*.* New York: McGraw-Hill; 2002. p. 87–118.

Drummond MF, Sculpher MJ, Claxton K, Stoddart GL, Torrance GW, editors. Methods for the economic evaluation of health care programmes. 4^th^ ed. Oxford: Oxford University Press; 2015.

European Centre for Disease Prevention and Control. Surveillance Atlas of Infectious Diseases; 2016. <https://atlas.ecdc.europa.eu/public/index.aspx>.

European Commission, Eurostat. Average number of usual weekly hours of work in main job, by sex, professional status, full-time/part-time and economic activity (from 2008 onwards, NACE Rev. 2) – hours (lfsa_ewhun2) [dataset]. Database: The European Union Labour Force Survey (EU-LFS); 2015. <http://ec.europa.eu/eurostat/data/database>.

European Commission, Eurostat. Employment rates by sex, age and citizenship (%) (lfsa_ergan) [dataset]. Database: The European Union Labour Force Survey (EU-LFS); 2015. <http://ec.europa.eu/eurostat/data/database>.

European Commission, Eurostat. Full-time and part-time employment by sex, age, and economic activity – NACE A10 (from 2008 onwards, NACE Rev. 2) – 1000 (lfsa_epgan2) [dataset]. Database: The European Union Labour Force Survey (EU-LFS); 2015. <http://ec.europa.eu/eurostat/data/database>.

European Commission, Eurostat. Life table (demo_mlifetable) [dataset]. Database: Mortality; 2014. <http://ec.europa.eu/eurostat/data/database>.

European Commission, Eurostat. Mean annual earnings by sex, age, and occupation – NACE Rev. 2, B-S excluding O (earn_ses14_28) [dataset]. Database: The Structure of Earnings Survey; 2014. <http://ec.europa.eu/eurostat/data/database>.

European Commission, Eurostat. Mean hourly earnings by sex, age, and occupation – NACE Rev. 2, B-S excluding O (earn_ses14_14) [dataset]. Database: The Structure of Earnings Survey; 2014. <http://ec.europa.eu/eurostat/data/database>.

European Commission, Eurostat. Structure of earnings survey: annual earnings (earn_ses_annual) [dataset]. Database: The Structure of Earnings Survey; 2014. <http://ec.europa.eu/eurostat/data/database>.

Federal Reserve Bank of St. Louis, Economic Research Division. U.S. / Euro Foreign Exchange Rate, U.S. Dollars to One Euro, Monthly, Not Seasonally Adjusted [dataset]. Database: Federal Reserve Economic Data; 2017. <https://fred.stlouisfed.org/>

GBD 2016 LRI Collaborators. Estimates of the global, regional, and national morbidity, mortality, and aetiologies of lower respiratory infections in 195 countries, 1990–2016: a systematic analysis for the Global Burden of Disease Study 2016. The Lancet. 2018;18(11): 1193–1194.

Global Burden of Disease. IHME; 2017. <http://ghdx.healthdata.org/gbd-2017>.

Global Burden of Disease Study 2015. Global Burden of Disease Study 2015 (GBD 2015) Socio-Demographic Index (SDI) 1980–2015. Seattle, United States: Institute for Health Metrics and Evaluation (IHME), 2016.

Harboe ZB, Dalby T, Weinberger DM, Benfield T, Mølbak K, Slotved HC, Suppli CH, Konradsen HB, Valentiner-Branth P. Impact of 13-valent pneumococcal conjugate vaccination in invasive pneumococcal disease incidence and mortality. Clin Infect Dis. 2014;59(8):1066–1073.

IDF Diabetes Atlas Group. Update of mortality attributable to diabetes for the IDF Diabetes Atlas: estimates for the year 2013. Diabetes Research and Clinical Practice. 2015;109(3):461–465.

International Standard Classification of Occupations. ISCO-08 part 3: Definitions of major groups, sub-major groups, minor groups and unit groups. 2008. p. 49, 545. <http://www.ilo.org/public/english/bureau/stat/isco/docs/groupdefn08.pdf>.

Jiang Y, Gauthier A, Annemans L, van der Linden M, Nicolas-Spony L, Bresse X. Cost-effectiveness of vaccinating adults with the 23-valent pneumococcal polysaccharide vaccine (PPV23) in Germany. Expert Rev Pharmacoecon Outcomes Res. 2012;12(5):645–660.

Klugman KP, Madhi SA, Huebner RE, Kohberger R, Mbelle N, Pierce N. A trial of a 9-valent pneumococcal conjugate vaccine in children with and those without HIV infection. N Engl J Med. 2003;349(14):1341–1348.

Kocabaş A. Kronik obstrüktif akciğer hastaliği epidemiyolojisi ve risk faktörleri. TTD Toraks Cerrahisi Bülteni. 2010;1(2):105–113.

Krol M, Brouwer W. How to estimate productivity costs in economic evaluations. Pharmacoeconomics. 2014;32(4):335–344.

Larson BA. Calculating disability-adjusted-life-years lost (DALYs) in discrete-time. Cost Effectiveness and Resource Allocation. 2013;11(8). https://resource-allocation.biomedcentral.com/articles/10.1186/1478-7547-11-18.

Mangen MJ, Rozenbaum MH, Huijts SM, van Werkhoven CH, Postma DF, Atwood M, Van Deursen AMM, van der Ende A, Grobbee DE, Sanders EAM, et al. Cost-effectiveness of adult pneumococcal conjugate vaccination in the Netherlands. Eur Respir J. 2015;46(5):1407–1416.

Ministry of Health, Turkey Public Health Agency. Circular on Risk Group Vaccination. No. 21001706. [Cited 2018 Dec 20]. [http://asirehberi.saglik.gov.tr/genelgeler/risk-grubu-genelgesi#](http://asirehberi.saglik.gov.tr/genelgeler/risk-grubu-genelgesi).

Multi-Society Task Force on PVS. Medical aspects of the persistent vegetative state. N Engl J Med. 1994;330(21):1499–1508.

National Foundation for Infectious Diseases. Important information about asthma and pneumococcal disease: talk to your patients about protecting themselves from this dangerous infection (2014). <http://www.adultvaccination.org/professional-resources/pneumo/asthma.pdf>.

National Transfer Accounts, Understanding the Generational Economy, 2.2.3: Labor income. Labor Income of the Self-Employed. <http://www.ntaccounts.org/web/nta/show/Methodology/2.2.3%20Labor%20Income>.

Oksuz L, Gurler N. Serotype Distribution and Antibiotic Resistance of *Streptococcus pnemoniae* Strains Isolated from the Adult Patients in a Turkish University Hospital. Mikroboyil Bul. 2017;195–208.

Organisation for Economic Cooperation and Development (OECD). Time use across the world. Database: Gender Data Portal; 2011. <https://www.oecd.org/gender/data/OECD_1564_TUSupdatePortal.xls>.

Rejas J, Cedillo S, Cifuentes I, Lwoff N. Cost-effectiveness of a pneumococcal sequential immunization with 13-valent pneumococcal conjugate vaccine in addition to 23-valent pneumococcal polysaccharide vaccine to immunocompetent older adults in Spain. Poster presented at:10^th^ International Symposium on Pneumococci and Pneumococcal Diseases (ISPPD); 2016 June 24-30; Glasgow, United Kingdom.

Roed C, Engsig FN, Omland LH, Skinhoj P, Obel N. Long-term mortality in patients diagnosed with pneumococcal meningitis: A Danish nationwide cohort study. Am J Epidemiol. 2010; 172(3):309–317.

Satman I, Omer B, Tutuncu Y, Kalaca S, Gedik S, Dinccag N, Karsidag K, Genc S, Telci A, Canbaz B, et al. Twelve-year trends in the prevalence and risk factors of diabetes and prediabetes in Turkish adults. European Journal of Epidemiology. 2013;28(2):169–180. https://www.ncbi.nlm.nih.gov/pmc/articles/PMC3604592/.

Schmand B, de Bruin E, de Gans J, van de Beek D. Cognitive functioning and quality of life nine years after bacterial meningitis. J Infect. 2010;61(4):330–334.

Søgaard M, Nielsen RB, Schønheyder HC, Nørgaard M, Thomsen RW. Nationwide trends in pneumonia hospitalization rates and mortality, Denmark 1997–2011. Respir Med. 2014;108(8):1214–1222.

Shiri T, Datta S, Madan J, Tsertsvadze A, Royle P, Keeling MJ, McCarthy ND, Petrou S. Indirect effects of childhood pneumococcal conjugate vaccination on invasive pneumococcal disease: a systematic review and meta-analysis. Lancet Global Health. 2017;5(1):e51–59.

Sortsø C, Green A, Jensen PB, Emneus M. Societal costs of diabetes mellitus in Denmark. Diabetic Medicine. 2016;33(7):877-885.

Torres A, Blasi F, Dartois N, Akova M. Which individuals are at increased risk of pneumococcal disease and why? Impact of COPD, asthma, smoking, diabetes, and/or chronic heart disease on community-acquired pneumonia and invasive pneumococcal disease. Thorax. 2015;70(10):984–989.

Türk Turaks Derneği. Bölüm 1.1: Astim tanim ve epıdemıyolojısı. In Türk Turaks Derneği, editor. Türk Turaks Derneği Astim Tani ve Tedavı Rehberı. Ankara: Türk Turaks Derneği; 2009. p. 1–4.

Turkish Statistical Institute (TurkStat). Average activity time per person by type of activity, sex, and age group [dataset]. Database: TurkStat, Time Use Survey; 2014-2015. <http://www.turkstat.gov.tr/PreTablo.do?alt_id=1009>.

Turkish Statistical Institute. Employment status by years [dataset]. 2014. <http://www.turkstat.gov.tr>.

Turkish Statistical Institute. Mean annual income at main job by employment status, 2006-2017 [dataset]. Database: Income and Living Conditions Survey. <http://www.turkstat.gov.tr>.

Turkish Statistical Institute. Mean annual income at main job by employment status, 2006-2017 [dataset]. Database: Income and Living Conditions Survey. <http://www.turkstat.gov.tr>.

United Nations. World population prospects: the 2015 revision. Population by age groups – both sexes [dataset]. New York: United Nations, Department of Economic and Social Affairs, Population Division; 2015. <https://esa.un.org/unpd/wpp/Download/SpecialAggregates/Ecological/>.

United Nations. World population prospects: the 2015 revision. Population by age groups – female [dataset]. New York: United Nations, Department of Economic and Social Affairs, Population Division; 2015. <https://esa.un.org/unpd/wpp/Download/SpecialAggregates/Ecological/>.

United Nations. World population prospects: the 2015 revision. Population by age groups – male [dataset]. New York: United Nations, Department of Economic and Social Affairs, Population Division; 2015. <https://esa.un.org/unpd/wpp/Download/SpecialAggregates/Ecological/>.

van de Beek D, de Gans J, Spanjaard L, Weisfelt M, Reitsma JB, Vermeulen M. Clinical features and prognostic factors in adults with bacterial meningitis. N Engl J Med. 2004;351(18):1849–1859.

van Hoek AJ, Miller E. Cost-effectiveness of vaccinating immunocompetent ≥65 year-olds with the 13-valent pneumococcal conjugate vaccine in England. PLoS ONE. 2016;11(2):e0149540.

van Werkhoven CH, Huijts SM, Bolkenbaas M, Webber C, Schmoele-Thoma B, Patterson SD, Gruber W, Grobbee DE, Bonten M. 13-valent pneumococcal conjugate vaccine efficacy is declining with old age: results from an exploratory analysis of the CAPITA trial. Poster presented at ID Week, Philadelphia, PA. 2014 Oct 10. <https://idsa.confex.com/idsa/2014/webprogram/Paper47255.html>.

Waight PA, Andrews NJ, Ladhani SN, Sheppard CL, Slack MPE, Miller E. Effect of the 13-valent pneumococcal conjugate vaccination on invasive pneumococcal disease in England and Wales years after its introduction: An observational cohort study. The Lancet. 2015;15(5):535–543. <https://www.thelancet.com/action/showPdf?pii=S1473-3099%2815%2970044-7>.

Weisfelt M, van de Beek D, Hoogman M, Hardeman C, de Gans J, Schmand B. Cognitive outcome in adults with moderate disability after pneumococcal meningitis. J Infect. 2006;52(6):433–439.

Weycker D, Farkouh RA, Strutton DR, Edelsberg J, Shea KM, Pelton SI. Rates and costs of invasive pneumococcal disease and pneumonia in persons with underlying medical conditions. BMC Health Services Research. 2016;16(1):182.

World Development Indicators, The World Bank. <https://databank.worldbank.org/reports.aspx?source=world-development-indicators>

World Health Organization International Agency for Research on Cancer. Turkey: Summary statistics (2018). The Global Cancer Observatory: 2018 May. <http://gco.iarc.fr/today/data/factsheets/populations/792-turkey-fact-sheets.pdf>

Wyrwich KW, Yu H, Sato R, Powers JH. Observational longitudinal study of symptom burden and time for recovery from community acquired pneumonia reported by older adults surveyed nationwide using the CAP burden of illness questionnaire. Patient Relat Outcome Meas. 2015;6:215–223.

1. Ministry of Health, Turkey Public Health Agency. Circular on Risk Group Vaccination. No. 21001706. [Cited 2018 Dec 20]. http://asirehberi.saglik.gov.tr/genelgeler/risk-grubu-genelgesi#. [↑](#footnote-ref-1)
2. Kocabaş. Kronik obstrüktif akciğer hastaliği epidemiyolojisi ve risk faktörleri. TTD Toraks Cerrahisi Bülteni. 2010;1(2):105–113. [↑](#footnote-ref-2)
3. Türk Turaks Derneği. Bölüm 1.1: Astim tanim ve epıdemıyolojısı. In Türk Turaks Derneği, editor. Türk Turaks Derneği Astim Tani ve Tedavı Rehberı*.* Ankara: Türk Turaks Derneği; 2009. p. 1–4. [↑](#footnote-ref-3)
4. Satman I, Omer B, Tutuncu Y, Kalaca S, Gedik S, Dinccag N, Karsidag K, Genc S, Telci A, Canbaz B, et al*.* Twelve-year trends in the prevalence and risk factors of diabetes and prediabetes in Turkish adults. European Journal of Epidemiology. 2013;28(2):169-180. https://www.ncbi.nlm.nih.gov/pmc/articles/PMC3604592/. [↑](#footnote-ref-4)
5. Değertekin M, Erol C, Ergene O, Tokgözoğlu L, Aksoy M, Erol MK, Eren M, Şahin M, Eroğlu E, et al. Türkite’deki kalp yetersizliği prevalansı ve öngördürücüleri: HAPPY çalışması. Türk Kardiyol Dern Arş. 2012;40(4):298–308. [↑](#footnote-ref-5)
6. World Health Organization International Agency for Research on Cancer. Turkey: Summary statistics (2018). The Global Cancer Observatory: 2018 May. [↑](#footnote-ref-6)
7. Ateş. Türkiye’de diyabet ve kronik böbrek hastalığı: CREDIT çalışması. Presentation by Türk Nefrologı Derneğı; see slides 18, 21, 48. http://www.nefroloji.org.tr/folders/file/hekimlik/salon2/Kenan_Ates.pdf. [↑](#footnote-ref-7)
8. Mangen MJ, Rozenbaum MH, Huijts SM, van Werkhoven CH, Postma DF, Atwood M, Van Deursen AMM, van der Ende A, Grobbee DE, Sanders EAM, et al. Cost-effectiveness of adult pneumococcal conjugate vaccination in the Netherlands. Eur Respir J. 2015;46(5):1407–1416. doi:10.1183/13993003.00325-2015. [↑](#footnote-ref-8)
9. Centers for Disease Control and Prevention. Lung disease including asthma and adult vaccination. Washington (DC): U.S. Department of Health and Human Services; 2016 May 2 [accessed on 2019 Sep 29]. https://www.cdc.gov/vaccines/adults/rec-vac/health-conditions/lung-disease.html. [↑](#footnote-ref-9)
10. Torres A, Blasi F, Dartois N, Akova M. Which individuals are at increased risk of pneumococcal disease and why? Impact of COPD, asthma, smoking, diabetes, and/or chronic heart disease on community-acquired pneumonia and invasive pneumococcal disease. Thorax. 2015;70(10): 984–989. [↑](#footnote-ref-10)
11. National Foundation for Infectious Diseases. Important information about asthma and pneumococcal disease: talk to your patients about protecting themselves from this dangerous infection (2014). http://www.adultvaccination.org/professional-resources/pneumo/asthma.pdf. [↑](#footnote-ref-11)
12. The values presented in this table do not always sum to one within a row because of rounding. [↑](#footnote-ref-12)
13. Global Burden of Disease. IHME (2017). <http://ghdx.healthdata.org/gbd-2017> [↑](#footnote-ref-13)
14. GBD 2016 LRI Collaborators. Estimates of the global, regional, and national morbidity, mortality, and aetiologies of lower respiratory infections in 195 countries, 1990–2016: a systematic analysis for the Global Burden of Disease Study 2016. The Lancet. 2018;18(11). Table 2: Global episodes due to each LRI aetiology. [↑](#footnote-ref-14)
15. GBD 2016 LRI Collaborators, 2018. [↑](#footnote-ref-15)
16. Akin L, Kaya M, Altinel, S, Durand L. Cost of pneumococcal infections and cost-effectiveness analysis of pneumococcal vaccination at risk adults and elderly in Turkey. Human vaccines. 2011;7(4),441–450. doi:10.4161/hv.7.4.14188. [↑](#footnote-ref-16)
17. Oksuz L, Gurler N. Serotype Distribution and Antibiotic Resistance of Streptococcus pnemoniae Strains Isolated from the Adult Patients in a Turkish University Hospital. Mikroboyil Bul 2017;195-208. doi 10.5578/mb.48638. [↑](#footnote-ref-17)
18. Ceyhan M, Dagan R, Sayiner A, Chernyshova L, Dinleyici EC, Hryniewicz W, Kulcsar A, Mad’arova L, Pazdiora P, Sidorenko S, et al. Surveillance of pneumococcal diseases in Central and Eastern Europe. Hum Vaccin Immunother, 2016;12(8)2124–2134. <http://dx.doi.org/10.1080/21645515.2016.1159363>. [↑](#footnote-ref-18)
19. Global Burden of Disease. IHME, 2017. [↑](#footnote-ref-19)
20. European Centre for Disease Prevention and Control. Surveillance Atlas of Infectious Diseases; 2016. Ameri<https://atlas.ecdc.europa.eu/public/index.aspx>. [↑](#footnote-ref-20)
21. The Socio-Demographic Index is a summary measure of a geography's socio-demographic development. It is based on average income per person, educational attainment, and total fertility. Global Burden of Disease Study 2015. Global Burden of Disease Study 2015 (GBD 2015) Socio-Demographic Index (SDI) 1980–2015. Seattle, United States: Institute for Health Metrics and Evaluation (IHME), 2016. [↑](#footnote-ref-21)
22. GBD 2016 LRI Collaborators, 2018. [↑](#footnote-ref-22)
23. Shiri T, Datta S, Madan T, Tsertsvadza A, Royle P, Keeling MJ, McCarthy ND, Petrou S. Indirect effects of childhood pneumococcal conjugate vaccination on invasive pneumococcal disease: a systematic review and meta-analysis. Lancet Global Health. 2017;5(1):e51-59. [↑](#footnote-ref-23)
24. Waight PA, Andrews NJ, Ladhani SN, Sheppard CL, Slack MPE, Miller E. Effect of the 13-valent pneumococcal conjugate vaccination on invasive pneumococcal disease in England and Wales years after its introduction: An observational cohort study. The Lancet. 2015;15(5):535–543. <https://www.thelancet.com/action/showPdf?pii=S1473-3099%2815%2970044-7>. [↑](#footnote-ref-24)
25. Harboe ZB, Dalby T, Weinberger DM, Benfield T, Mølbak K, Slotved HC, Suppli CH, Konradsen HB, Valentiner-Branth P. Impact of 13-valent pneumococcal conjugate vaccination in invasive pneumococcal disease incidence and mortality. Clin Infect Dis. 2014;59(8):1066–1073. [↑](#footnote-ref-25)
26. Rejas J, Cedillo S, Cifuentes I, Lwoff N. Cost-effectiveness of a pneumococcal sequential immunization with 13-valent pneumococcal conjugate vaccine in addition to 23-valent pneumococcal polysaccharide vaccine to immunocompetent older adults in Spain. Poster presented at the 10^th^ International Symposium on Pneumococci and Pneumococcal Diseases (ISPPD); 2016 June 24-30; Glasgow, United Kingdom. [↑](#footnote-ref-26)
27. van Hoek AJ, Miller E. Cost-effectiveness of vaccinating immunocompetent ≥65 year olds with the 13-valent pneumococcal conjugate vaccine in England. PLoS ONE. 2016;11(2):e0149540. [↑](#footnote-ref-27)
28. Mangen et al. (2015). Supplementary material, Table S.5. [↑](#footnote-ref-28)
29. van Werkhoven CH, Huijts SM, Bolkenbaas M, Webber C, Schmoele-Thoma B, Patterson SD, Gruber W, Grobbee DE, Bonten M. 13-valent PCV efficacy is declining with old age: results from an exploratory analysis of the CAPITA trial. Poster presented at ID Week, Philadelphia, PA. 2014 Oct 10. https://idsa.confex.com/idsa/2014/webprogram/Paper47255.html. [↑](#footnote-ref-29)
30. Bonten M, Huijts SM, Bolkenbaas M, Webber C, Patterson S, Gault S, van Werkhoven CH, van Duersen AMM, Sanders EAM, Verheij TJM, et al. Polysaccharide conjugate vaccine against pneumococcal pneumonia in adults. N Engl J Med. 2015;372(12):1114–1125. [↑](#footnote-ref-30)
31. Mangen et al. (2015), Supplementary material, Note to Figure S.2. [↑](#footnote-ref-31)
32. Bonten et al. (2015). Table 2. [↑](#footnote-ref-32)
33. Mangen et al. (2015). p. 3. [↑](#footnote-ref-33)
34. Mangen et al. (2015) derive this relative difference from the differential vaccine efficacy observed in children with and without HIV from a trial of a 9-valent pneumococcal conjugate vaccine. [↑](#footnote-ref-34)
35. Akyil FT, Yalcinsoy MA, Hazar A, Cilli B, Celenk O, Kilic A, Sayiner N, Kokturk A, Sakar CA, Filiz E, Cakir E. Prognosis of hospitalized patients with community-acquired pneumonia. Pulmonology. 2018;24(3):164-169. ISSN 2531–0437. https://doi.org/10.1016/j.rppnen.2017.07.010. [↑](#footnote-ref-35)
36. van de Beek D, de Gans J, Spanjaard L, Weisfelt M, Reitsma JB, Vermeulen M. Clinical features and prognostic factors in adults with bacterial meningitis. N Engl J Med. 2004;351(18):1849–59. [↑](#footnote-ref-36)
37. European Commission, Eurostat. Life table (demo_mlifetable) [dataset]. Database: Mortality; 2014. http://ec.europa.eu/eurostat/data/database. [↑](#footnote-ref-37)
38. IDF Diabetes Atlas Group. Update of mortality attributable to diabetes for the IDF Diabetes Atlas: estimates for the year 2013. Diabetes Research and Clinical Practice. 2015;109(3):461–65. See Table 1. [↑](#footnote-ref-38)
39. United Nations. World population prospects: the 2015 revision. Population by age groups – female [dataset]. New York: United Nations, Department of Economic and Social Affairs, Population Division; 2015. https://esa.un.org/unpd/wpp/Download/SpecialAggregates/Ecological/. [↑](#footnote-ref-39)
40. United Nations. World population prospects: the 2015 revision. Population by age groups – male [dataset]. New York: United Nations, Department of Economic and Social Affairs, Population Division; 2015. https://esa.un.org/unpd/wpp/Download/SpecialAggregates/Ecological/. [↑](#footnote-ref-40)
41. Krol M, Brouwer W. How to estimate productivity costs in economic evaluations. Pharmacoeconomics. 2014;32(4):335–344. [↑](#footnote-ref-41)
42. Multi-Society Task Force on PVS. Medical aspects of the persistent vegetative state. N Engl J Med. 1994;330(21):1499-1508. [↑](#footnote-ref-42)
43. van de Beek et al*.* (2004). [↑](#footnote-ref-43)
44. International Standard Classification of Occupations. ISCO-08 part 3: Definitions of major groups, sub-major groups, minor groups and unit groups. 2008. p. 49. http://www.ilo.org/public/english/bureau/stat/isco/docs/groupdefn08.pdf. [↑](#footnote-ref-44)
45. International Standard Classification of Occupations. ISCO-08 part 3, 2008. [↑](#footnote-ref-45)
46. European Commission, Eurostat. Mean hourly earnings by sex, age, and occupation – NACE Rev. 2, B-S excluding O (earn_ses14_14) [dataset]. Database: The Structure of Earnings Survey; 2014. http://ec.europa.eu/eurostat/data/database. [↑](#footnote-ref-46)
47. Weisfelt M, van de Beek D, Hoogman M, Hardeman C, de Gans J, Schmand B. Cognitive outcome in adults with moderate disability after pneumococcal meningitis. J Infect. 2006;52(6):433–9. Table 3. See Table 2 for the duration. [↑](#footnote-ref-47)
48. Schmand B, de Bruin E, de Gans J, van de Beek D. Cognitive functioning and quality of life nine years after bacterial meningitis. J Infect. 2010;61(4):330–334. [↑](#footnote-ref-48)
49. van de Beek et al*.* (2004). [↑](#footnote-ref-49)
50. European Commission, Eurostat. Employment rates by sex, age and citizenship (%) (lfsa_ergan) [dataset]. Database: The European Union Labour Force Survey (EU-LFS); 2015. http://ec.europa.eu/eurostat/data/database. [↑](#footnote-ref-50)
51. European Commission, Eurostat. Mean annual earnings by sex, age, and occupation – NACE Rev. 2, B-S excluding O (earn_ses14_28) [dataset]. Database: The Structure of Earnings Survey; 2014. http://ec.europa.eu/eurostat/data/database. [↑](#footnote-ref-51)
52. European Commission, Eurostat. Mean hourly earnings by sex, age, and occupation, 2014. http://ec.europa.eu/eurostat > Data > Database > Database by themes > Population and social conditions > Labour Market > Earnings > Structure of Earnings Survey 2014 > Hourly Earnings > Mean Hourly Earnings by Sex, Age, and Occupation-NACE Rev. 2, B-S excluding O > earn_ses14_14. [↑](#footnote-ref-52)
53. World Development Indicators, The World Bank. <https://databank.worldbank.org/reports.aspx?source=world-development-indicators> [↑](#footnote-ref-53)
54. European Commission, Eurostat. Mean annual earnings by sex, age, and occupation, 2014. [↑](#footnote-ref-54)
55. Turkish Statistical Institute (TurkStat). Mean annual income at main job by employment status, 2006-2017 [dataset]. Database: Income and Living Conditions Survey. http://www.turkstat.gov.tr > Statistics by Theme > Income, Living, Consumption and Poverty > Income Distribution and Living Conditions Statistics > Statistical Tables and Dynamic Search > Income Distribution Statistics > Income and Living Conditions Survey > Turkey > Individual Income > Mean annual income at main job by employment status, 2006–2015. [↑](#footnote-ref-55)
56. Turkish Statistical Institute (TurkStat). Employment status [dataset]. 2014. http://www.turkstat.gov.tr > Main Statistics > Employment, Unemployment and Wages > Employment Status. [↑](#footnote-ref-56)
57. Turkish Statistical Institute (TurkStat). Mean annual income at main job by employment status. [↑](#footnote-ref-57)
58. National Transfer Accounts, Understanding the Generational Economy, 2.2.3: Labor income. Labor Income of the Self-Employed. <http://www.ntaccounts.org/web/nta/show/Methodology/2.2.3%20Labor%20Income>. [↑](#footnote-ref-58)
59. International Standard Classification of Occupations, ISCO-08 part 3. Definitions of major groups, sub-major groups, minor groups and unit groups. 2008. p. 545. <http://www.ilo.org/public/english/bureau/stat/isco/docs/groupdefn08.pdf>. [↑](#footnote-ref-59)
60. International Standard Classification of Occupations, ISCO-08 part 3. Definitions of major groups, sub-major groups, minor groups and unit groups. 2008. p. 49. <http://www.ilo.org/public/english/bureau/stat/isco/docs/groupdefn08.pdf>. [↑](#footnote-ref-60)
61. We computed these probabilities to equal the ratio of full-time employed to the total number of employed individuals in 2015 from this table whose numbers (in thousands) are excerpted from Eurostat:

    | **Age** | **Source** | **Total number of employed individuals, in thousands** | **Total number of fully employed individuals, in thousands** |
    | --- | --- | --- | --- |
    | 50–59 | Eurostat | 3,402.7 | 2,870.8 |
    | 50–64 | Eurostat | 4,198.5 | 3,479.6 |
    | 60–64 | Inferred from previous two rows | 795.8 | 608.8 |
    | 65+ | Eurostat | 733.0 | 519.0 |
    | 65–74 | Inferred from rows immediately previous and following | 614.6 | 440.5 |
    | 75+ | Eurostat | 118.4 78.5 | |

    Source: European Commission, Eurostat. Full-time and part-time employment by sex, age, and economic activity – NACE A10 (from 2008 onwards, NACE Rev. 2) – 1000 (lfsa_epgan2) [dataset]. The European Union Labour Force Survey (EU-LFS); 2015. http://ec.europa.eu/eurostat/data/database. [↑](#footnote-ref-61)
62. European Commission, Eurostat. Average number of usual weekly hours of work in main job, by sex, professional status, full-time/part-time and economic activity (from 2008 onwards, NACE Rev. 2) – hours (lfsa_ewhun2) [dataset]. Database: The European Union Labour Force Survey (EU-LFS); 2015. http://ec.europa.eu/eurostat/data/database. Note that this column represents the number of hours actually worked by each population of interest, not the number of hours required to be considered a full-time or part-time employee. [↑](#footnote-ref-62)
63. European Commission, Eurostat. Structure of earnings survey: annual earnings (earn_ses_annual) [dataset]. Database: The Structure of Earnings Survey; 2014. http://ec.europa.eu/eurostat/data/database. [↑](#footnote-ref-63)
64. Sortsø C, Green A, Jensen PB, Emneus M. Societal costs of diabetes mellitus in Denmark. Diabetic Medicine. 2016;33(7):877. [↑](#footnote-ref-64)
65. Sortsø et al. (2016). [↑](#footnote-ref-65)
66. For example, Sortsø et al. (2016) do not provide these measures. [↑](#footnote-ref-66)
67. The American Diabetes Association’s approach is to estimate the amount of non-market product lost to diabetes using the amount of absenteeism in the working-age diabetic population. American Diabetes Association. Economic costs of diabetes in the US in 2012. Diabetes Care. 2013;36(6):1033–1046. [↑](#footnote-ref-67)
68. European Commission, Eurostat. Average number of usual weekly hours of work in main job, by sex, professional status, full-time/part-time and economic activity. [↑](#footnote-ref-68)
69. Turkish Statistical Institute (TurkStat). Average activity per person by type of activity, sex, and age group [dataset]. Database: TurkStat, Time Use Survey; 2014-2015. http://www.turkstat.gov.tr/PreTablo.do?alt_id=1009 > Statistical Tables and Dynamic Search > Use of Time > Average activity time per person by type of activity, sex, and age group. [↑](#footnote-ref-69)
70. Economic Cooperation and Development. Time use across the world [dataset]. Database: Gender Data Portal; 2011. https://www.oecd.org/gender/data/OECD_1564_TUSupdatePortal.xls. [↑](#footnote-ref-70)
71. See the “Turkey Activity Categories” column in the “Activity Categories” worksheet of https://www.oecd.org/gender/data/OECD_1564_TUSupdatePortal.xls. [↑](#footnote-ref-71)
72. See equation (1) in Larson BA. Calculating disability-adjusted-life-years lost (DALYs) in discrete-time. Cost Effectiveness and Resource Allocation. 2013;11(8). https://resource-allocation.biomedcentral.com/articles/10.1186/1478-7547-11-18. [↑](#footnote-ref-72)
73. See, for example, DeLong JB. The theory of economic growth. In: DeLong JB, editor. Macroeconomics*.* New York: McGraw-Hill; 2002. p. 87–118. [↑](#footnote-ref-73)
74. World Development Indicators, The World Bank. http://databank.worldbank.org/data/home.aspx > World Development Indicators > Country = all > Series = GDP per capita, PPP (constant 2011 international $) > Time = 1995–2015. [↑](#footnote-ref-74)
75. United Nations. World population prospects: the 2015 revision. Population by age groups – both sexes [dataset]. New York: United Nations, Department of Economic and Social Affairs, Population Division; 2015. https://esa.un.org/unpd/wpp/Download/SpecialAggregates/Ecological/. [↑](#footnote-ref-75)
76. Multi-Society Task Force on PVS. Medical aspects of the persistent vegetative state. N Engl J Med. 1994;330(21):1499-1508. [↑](#footnote-ref-76)
77. van de Beek et al. (2004). [↑](#footnote-ref-77)
78. Balia S, Brau R. A country for old men? Long-term home care utilization in Europe. Health Econ. 2014;23(10):1185–212. See the “LTC = 1” column of Table 1, which we use in our measurement of the long-term care burden of a persistent vegetative state following PD because the GOS defines “severe disability” as inability to live independently. [↑](#footnote-ref-78)
79. European Commission, Eurostat. Mean hourly earnings by sex, age, and occupation. The mean hourly rate from Eurostat is €3.84 based on establishments with at least 10 employees. Our adjusted hourly rate is €3.10 (see Section A4.1.2.i). [↑](#footnote-ref-79)
80. European Commission, Eurostat. Mean hourly earnings by sex, age, and occupation. The mean hourly rate from Eurostat is €2.41 based on establishments with at least 10 employees. Our adjusted hourly rate is €2.01 (see Section A4.1.2.i). [↑](#footnote-ref-80)
81. European Commission, Eurostat. Mean hourly earnings by sex, age, and. The mean hourly rate from Eurostat is €8.15 based on establishments with at least 10 employees. Our adjusted hourly rate is €6.64 (see Section A4.1.2.i). [↑](#footnote-ref-81)
82. European Commission, Eurostat employment rates by age, sex, and citizenship. [↑](#footnote-ref-82)
83. Roed C, Engsig FN, Omland LH, Skinhoj P, Obel N. Long-term mortality in patients diagnosed with pneumococcal meningitis: A Danish nationwide cohort study. Am J Epidemiol. 2010;172(3):309–317. See Figure 2. [↑](#footnote-ref-83)
84. For ages 18-19 years, we use the same MRR that we assume for ages 20-29 years. The reason is that since 18 and 19 year-olds are in the upper bound of the 0-19 age group, and the MRR for this age group is approximately twice that of the MRR for ages 20-29, it seems more likely that the MRR for ages 18 and 19 is closer to 4 than 8. [↑](#footnote-ref-84)
85. Eurostat Life Table. http://ec.europa.eu/eurostat > Data > Database > Database by themes > Population and social conditions > Demography and Migration > Mortality > Life Table > demo_mlifetable. [↑](#footnote-ref-85)
86. Wyrwich KW, Yu H, Sato R, Powers JH. Observational longitudinal study of symptom burden and time for recovery from community acquired pneumonia reported by older adults surveyed nationwide using the CAP burden of illness questionnaire. Patient Relat Outcome Meas. 2015;6:215–223. [↑](#footnote-ref-86)
87. See Table 3 of Wyrwich et al. (2015) for the numbers in this formula. [↑](#footnote-ref-87)
88. Jiang Y, Gauthier A, Annemans L, van der Linden M, Nicolas-Spony L, Bresse X. Cost-effectiveness of vaccinating adults with the 23-valent pneumococcal polysaccharide vaccine (PPV23) in Germany. Expert Rev Pharmacoecon Outcomes Res. 2012;12(5):645–60. See Table 3. [↑](#footnote-ref-88)
89. Wyrwich et al. (2015). [↑](#footnote-ref-89)
90. For example, we assume that changes in LOS result in proportional changes in the number of days until full productivity is reached and the number of days until an individual returns to work. [↑](#footnote-ref-90)
91. Weycker D, Farkouh RA, Strtton DR, Edelsberg J, Shea KM, Pelton SI. Rates and costs of IPD and pneumonia in persons with underlying medical conditions. BMC Health Services Research*.* 2016;16(1):182. [↑](#footnote-ref-91)
92. World population prospects: the 2015 revision. Population By Age Groups – Both Sexes. [↑](#footnote-ref-92)
93. For example, for adults 65 and older, the mean LOS for the healthy group was 9.7 days, which rose to 10.7 for the at-risk group, but fell back to 9.8 days in the high-risk group. The 18- to 64-year-old age group showed the opposite pattern, with the corresponding risk group-specific mean LOS being 12.2 days, 11.1 days, and 12.5 days, respectively. The corresponding values for the under-18 age group were 11.0 days, 17.3 days, and 8.6 days, respectively. Weycker et al. (2016), Table 2. [↑](#footnote-ref-93)
94. World population prospects: The 2015 revision. Population by age groups – both sexes. [↑](#footnote-ref-94)
95. See Ceyhan et al. (2016) Table 5. [↑](#footnote-ref-95)
96. World population prospects: The 2015 revision. Population by age groups – both sexes. [↑](#footnote-ref-96)
97. Data are from OECD. Stat Economic Outlook No 101 – June 2017. http://stats.oecd.org/ > Data by theme > Economic Projections > OECD Economic Outlooks > OECD Economic Outlook Latest edition > Economic Outlook No 101 – June 2017 > EO By Subject (GDP, Unemployment…) > country = Turkey; variable = “Gross domestic product, deflator, market prices;” time = 2014,2017. [↑](#footnote-ref-97)
98. Federal Reserve Economic Data U.S. / Euro Foreign Exchange Rate. https://fred.stlouisfed.org/ > Browse data by Category > Money, Banking, & Finance > Exchange Rates > By Country > Euro > U.S. / Euro Foreign Exchange Rate > Monthly. [↑](#footnote-ref-98)
